# Transcriptomic Responses of Zebrafish Embryos to Environmentally Relevant, Low-Dose (2-Ethylhexyl) Phthalate Exposure at 96–120 hpf

**DOI:** 10.3390/genes17030257

**Published:** 2026-02-25

**Authors:** Mariagiovanna Pais, Kate McCafferty, Guillermo Lopez Campos, Gary Hardiman

**Affiliations:** 1 Faculty of Medicine, Health and Life Sciences, School of Biological Sciences, Institute for Global Food Security, 19 Chlorine Gardens, Belfast BT9 5DL, UK; mpais01@qub.ac.uk (M.P.);; 2 Wellcome-Wolfson Institute for Experimental Medicine, Queen’s University, 97 Lisburn Rd, Belfast BT9 7BL, UK; g.lopezcampos@qub.ac.uk; 3 Department of Medicine, University of California San Diego, La Jolla, CA 92093, USA

**Keywords:** zebrafish, DEHP, endocrine disruptors, developmental exposure, RNA-seq, transcriptomics, pathway enrichment, protein–protein interaction networks, estrogen signaling, androgen signaling

## Abstract

Background: Di(2-ethylhexyl) phthalate (DEHP) is a high-production-volume plasticizer and ubiquitous environ-mental contaminant with established endocrine-disrupting potential. While zebrafish transcriptomic studies have typically used high concentrations and long exposure windows, less is known about genome-wide responses during late embryogenesis/early larval maturation under environmentally relevant exposures. Here we profiled whole-organism transcriptomic responses to a short DEHP exposure during a developmentally sensitive transition (96–120) hours post-fertilization, hpf) and interpreted responses using differential expression, enrichment analyses, and endocrine-focused protein–protein interaction (PPI) network modeling. Methods: Wild-type AB zebrafish lar-vae (96 hpf) were exposed to DEHP at [10^−9^ M] or solvent control for 24 h. Larvae were pooled per replicate (25 lar-vae/pool) and processed for poly(A)-selected RNA-seq. Reads were quality-controlled, aligned to the Danio rerio reference genome, and quantified at gene- level. Differential expression was performed using DESeq2. Functional enrichment used KEGG over-representation analysis (ORA) and gene set enrichment analysis (GSEA). Zebrafish genes were mapped to human orthologs for GO/KEGG and STRING-based endocrine subnetworks, which were visualized and interrogated using STRINGdb and visNetwork. Results: Low-dose, short-term exposure does not produce large gene-level effects but induces coordinated, pathway-level transcriptional remodeling. KEGG ORA showed significant enrichment of MAPK signaling and regulation of actin cytoskeleton with additional enrichment of axon guidance and neuroactive ligand–receptor interaction. GSEA detected coordinated downregulation of KEGG neurodegeneration collections with negative normalized enrichment scores reflecting shared gene sets re-lated to mitochondrial function, proteostasis, cytoskeletal organization, and stress-response pathways. Endo-crine-focused STRING subnetworks indicated consistent downregulation of CYP19A1 within estrogen metabo-lism/biosynthesis modules and downregulation of upstream androgen biosynthetic enzymes HSD3B2 and CYP17A1, alongside upregulation of HSD17B3 and proteostasis-associated factors including DNAJA1. Endocrine network to-pology highlighted regulatory and cofactor nodes affecting receptor-linked transcription, consistent with indirect endocrine modulation rather than large receptor-transcript changes. Conclusions: In summary, this study demon-strates that exposure to low-dose DEHP during a critical period of zebrafish embryonic development is associated with modest but coordinated transcriptomic changes across multiple biological pathways. Pathway enrichment and network-based analyses highlight estrogen- and androgen-associated processes, along with broader signaling, met-abolic, and structural pathways, as transcriptionally responsive during this window. Importantly, these findings reflect molecular-level associations rather than direct evidence of functional or physiological endocrine disruption. Instead, they identify candidate pathways and regulatory networks that may be sensitive to low-level environmen-tal exposure and warrant further investigation. Collectively, this work underscores the value of systems-level tran-scriptomic approaches for detecting subtle, pathway-wide responses to environmentally relevant exposures during development.

## 1. Introduction

Phthalates, esters of phthalic acid, are high-production-volume chemicals widely used as plasticizers in polyvinyl chloride (PVC) and numerous consumer products, including cosmetics, medical devices, and packaging materials [1,2]. Because they are not covalently bound to polymer matrices, phthalates can leach into the environment and have become ubiquitous contaminants of air, water, soil, and food [3]. Human biomonitoring consistently detects phthalate metabolites in urine, confirming widespread population exposure [4,5]. Among the most prevalent compounds are di-2-ethylhexyl phthalate (DEHP), its monoester metabolite mono(2-ethylhexyl) phthalate (MEHP), di-n-butyl phthalate (DnBP), and butyl benzyl phthalate (BBzP) [6].

Experimental animal studies demonstrate that several phthalates disrupt reproductive and developmental processes, largely through anti-androgenic or oxidative stress-mediated mechanisms [7,8,9]. In rodents, prenatal exposure to DEHP or DBP produces phenotypes consistent with human testicular dysgenesis syndrome, including cryptorchidism, hypospadias, and reduced sperm output [10,11]. Despite these findings, molecular-level data in non-mammalian systems, especially during early development, remain limited. Zebrafish embryos are a valuable vertebrate model for assessing developmental toxicity and genome-wide responses to endocrine disruptors [12]. However, transcriptomic investigations of DEHP and MEHP exposure during zebrafish embryogenesis are sparse. Across zebrafish developmental studies, exposure to the phthalate monoester MEHP has been consistently shown to disrupt transcriptional programs critical for organogenesis and metabolic homeostasis. In an early genome-wide RNA-seq analysis, Jacobs et al. (2018) exposed embryos from 3 to 168 hpf and profiled transcriptomes at ~100 hpf, identifying pronounced downregulation of pancreas lineage genes (including *insa*, *sst2*, and *ptf1a*) alongside altered redox and glutathione pathway genes (*gstp1* and *gsr*), changes that were accompanied by pancreatic hypoplasia [13]. Follow-up work using a similar exposure window (6–120 hpf; 200 µg/L) and phenotypic plus targeted qPCR approaches demonstrated persistent metabolic consequences, notably larval steatosis observed up to 15 dpf, consistent with oxidative stress pathway engagement previously highlighted by RNA-seq [14]. More recently, Liu et al. compared MEHP and its parent compound DEHP up to 120 hpf using RNA-seq, reporting enrichment of pathways related to nervous system development, visual perception, synaptic signaling, oxidative stress, and apoptosis, which correlated with measurable locomotor deficits [15]. Complementing these genome-wide studies, Naïja and colleagues showed that embryonic DEHP exposure (0–96 hpf) perturbs the expression of key cardiac developmental markers (*gata4/5/6* and *tbx5*) in whole embryos, highlighting cardiovascular sensitivity even in the absence of transcriptome-wide profiling [16]. Together, these studies indicate that early-life exposure to DEHP metabolites elicits coordinated, tissue-relevant transcriptional disruptions with lasting functional outcomes in zebrafish [16]. DEHP was selected as the parent compound of environmental relevance due to its widespread use and occurrence in environmental matrices. Although human internal exposure is often dominated by its monoester metabolite, MEHP, the present study was designed as a hypothesis-generating assessment of whether low-dose DEHP exposure during a sensitive developmental window elicits coordinated transcriptomic responses.

The exposure concentrations used in these experimental studies (10^−4^–10^−6^ M DEHP and 10^−5^–10^−6^ M MEHP) substantially exceed typical environmental and physiological levels encountered by humans. Under real-world conditions, DEHP exposure is generally low, and the parent compound is rarely detected in blood due to rapid metabolic conversion to MEHP. Human biomonitoring studies typically report MEHP concentrations in the nanomolar range (~1–100 nM; 10^−9^–10^−7^ M), while environmental concentrations of DEHP in air, water, and soil are usually in the microgram- to nanogram-per-liter range, corresponding to low nanomolar or sub-nanomolar levels (≤10^−8^ M). Consequently, while such doses are valuable for identifying susceptible developmental pathways and potential modes of toxicity, they should not be interpreted as directly reflective of typical environmental or physiological exposure scenarios [13,15,16]. The exposure concentration used in this study (10^−9^ M) was selected to model environmentally and physiologically relevant phthalate exposure levels and to evaluate whether such low-dose exposure elicits detectable molecular responses during early development. This concentration overlaps with DEHP and MEHP levels reported in environmental matrices, including drinking water and surface waters, and is consistent with nanomolar MEHP concentrations measured in human biomonitoring studies. By employing this exposure level, the present study aims to assess subtle transcriptomic perturbations that may occur at real-world exposure concentrations rather than overt toxicological effects. This study targets embryo-stage exposure during the 96–120 hpf window, a critical developmental period in zebrafish when organ systems transition from structural formation to functional maturation. Because this interval encompasses rapid metabolic, endocrine, and neurobehavioral development, perturbations during this stage are particularly informative for detecting subtle functional and transcriptomic effects of chemical exposure [17].

Zebrafish toxicology studies have traditionally focused on early embryonic stages that emphasize morphogenesis and gross structural outcomes. While these early windows are well suited for detecting overt developmental abnormalities, they may be less sensitive to subtle molecular perturbations arising from environmentally relevant, low-dose exposures. In the present study, we therefore focused on the 96–120 h post-fertilization (hpf) interval, which represents a transitional phase from organogenesis to functional maturation. During this period, major organ systems are structurally established but continue to undergo rapid physiological integration, metabolic activation, and regulatory refinement. We reasoned that transcriptomic profiling during this functional maturation window would increase sensitivity to coordinated pathway- and network-level responses to low-dose DEHP exposure, enabling detection of modest but biologically organized transcriptional changes that may not be evident during earlier morphogenetic stages.

## 2. Methods

### 2.1. Experimental Design and Animal Care

All experiments were conducted in compliance with institutional animal welfare guidelines, using zebrafish larvae to minimize potential distress and avoid nociceptor activation. Wild-type AB strain zebrafish were bred under controlled laboratory conditions (28 ± 0.5 °C; 14 h light/10 h dark photoperiod). Eight parental pairs were used to minimize genetic variability while maintaining adequate biological replication. Zebrafish embryos and larvae were used prior to 120 h post-fertilization (hpf), a developmental stage that is widely recognized in regulatory frameworks as an early-life phase with reduced welfare concerns. The experimental design adhered to the principles of the 3Rs (Replacement, Reduction, and Refinement), with efforts made to minimize handling, exposure duration, and animal use while maintaining scientific validity.

### 2.2. Exposure Setup

Petri dishes (25 mL per dish) were prepared for the different experimental groups following a previously published experimental design [18]. One dish contained water supplemented with 5.8 nM/5.8 × 10^−9^ M di(2-ethylhexyl) phthalate (DEHP), a second dish contained water with 0.65 nM 17-α-ethinylestradiol/6.5 × 10^−10^ M (EE2; positive control), and a third dish contained water with ethanol only, serving as the negative exposure control. The DEHP concentration used is considered environmentally relevant. All chemicals were dissolved in ethanol to generate stock solutions, from which final working concentrations were prepared. Experimental groups consisted of 50 larvae precisely staged at 96 h post-fertilization (hpf). Larvae were exposed for 24 h to the following treatments: negative control (ethanol at concentrations equivalent to those used in treatment dilutions), DEHP, or EE2 (positive control). Each group was maintained in 25 mL of exposure medium within sterile Petri dishes and incubated at 28 °C under dark conditions for 24 h. Four independent biological replicates were conducted per treatment to enhance statistical robustness and minimize experimental variability. Due to the short exposure duration, no media renewal was performed. This design minimized handling-related stress and developmental disturbance during a sensitive late embryonic window. Exposure vessels and handling procedures were identical across all treatments to ensure comparability.

### 2.3. RNA Extraction and Sequencing

Total RNA was isolated from zebrafish embryos samples using TRIzol reagent (Invitrogen/Thermo Fisher Scientific, (Waltham, MA, USA)), followed by further purification with the RNeasy Mini Kit (Qiagen, Valencia, CA, USA). During purification, all samples underwent on-column DNase digestion to eliminate genomic DNA contamination. RNA concentration was determined by measuring absorbance at 260 nm using an ND-1000 spectrophotometer (NanoDrop, Wilmington, DE, USA). RNA integrity was assessed using the Agilent 6000 Nano LabChip assay (Agilent Technologies, Santa Clara, CA, USA). Only samples with RNA integrity numbers (RINs) greater than 7.0 were used for downstream genomic analyses. A total of 18 samples were processed for RNA extraction, including six EE2-exposed, six DEHP-exposed, and six control embryos. To prepare RNA-seq libraries using the TruSeq RNA Sample Prep Kit (Illumina, San Diego, CA, USA), 100–200 ng of total RNA was used following the protocol described by the manufacturer. High-throughput sequencing (HTS) was performed using an Illumina GAIIX with each sample sequenced to a minimum depth of ~2 million reads. A single-end 50 cycle sequencing strategy was employed. Data were subjected to Illumina quality control (QC) procedures (>80% of the data yielded a Phred score of 30). RNA-seq data have been submitted to the NCBI Gene Expression Omnibus, accession number GSE100367. Secondary analysis was carried out as described previously [18,19,20]. An automated RNA-seq workflow was used to process the data [21,22], including (1) data validation and quality control, (2) read alignment to the zebrafish genome (GRCz10) using TopHat2 [23], which revealed >73% mapping, (3) generation of gene-level count data with HTSeq [24], and (4) differential expression analysis with DEseq2 [20,25]. Transcript count data from DESeq2 analysis of the samples were sorted according to their adjusted *p*-value (or q-value), which is the smallest false discovery rate (FDR) at which a transcript is called significant. FDR is the expected fraction of false positive tests among significant tests and was calculated using the Benjamini and Hochberg multiple testing adjustment procedure [25,26,27]. Functional enrichment was conducted with clusterProfiler [28] (v4.6.2) together with org.Hs.eg.db (v3.17.0) [29], and the resulting GO terms were further refined through semantic similarity and redundancy reduction using GOSemSim (v2.24.0) [28] and rrvgo (v1.10.0) [30].

### 2.4. Pathway Enrichment Analysis and Gene Set Enrichment Analysis (GSEA)

Kyoto Encyclopedia of Genes and Genomes (KEGG) pathway analyses were performed to identify biological pathways associated with transcriptional changes following exposure. Two complementary approaches were applied, over-representation analysis (ORA) and gene set enrichment analysis (GSEA), implemented in R, to capture both discrete and graded pathway-level responses. For ORA, differentially expressed genes (DEGs) were first identified based on predefined statistical thresholds (adjusted *p*-value and fold-change criteria, as described above). Enrichment analysis was then conducted by testing whether KEGG pathways were statistically over-represented among the DEG list relative to the background of all expressed genes, using a hypergeometric framework as implemented in established R-based enrichment workflows, using the clusterProfiler package [28].

In parallel, GSEA was performed using a ranked list of all expressed genes, ordered by a continuous metric of differential expression (e.g., log_2_ fold change or test statistic), without imposing an arbitrary significance cutoff. This approach evaluates whether genes belonging to predefined KEGG pathways are non-randomly distributed toward the top or bottom of the ranked gene list, allowing detection of coordinated but modest expression changes across functionally related gene sets. GSEA was conducted in R according to established methods with pathway significance assessed using permutation-based testing and false discovery rate (FDR) correction [31]. Results from ORA and GSEA were interpreted in a complementary manner. ORA was used to highlight pathways driven by strongly differentially expressed genes, whereas GSEA provided sensitivity to more subtle, coordinated transcriptional shifts across entire pathways. KEGG pathways meeting the specified significance thresholds in either analysis were considered for downstream interpretation and biological contextualization.

### 2.5. Protein–Protein Interaction (PPI) Analysis

PPI analysis was carried out using STRINGdb (v2.10.5) [32], which provides programmatic access to the STRING human interactome (taxon 9606). Human protein–protein interaction networks were used because of their greater annotation depth and connectivity relative to available zebrafish databases. Although many core regulatory interactions are evolutionarily conserved, species- and developmental stage-specific differences may not be fully captured, and the resulting network modules should therefore be interpreted as putative conserved interaction frameworks rather than definitive zebrafish-specific interaction maps. To focus specifically on endocrine pathways, genes annotated to estrogen- and androgen-related biological processes were selected based on Gene Ontology terms, including estrogen metabolic and biosynthetic processes, cellular responses to estrogen stimulus, estrogen receptor signaling and response element binding, as well as androgen metabolic and biosynthetic processes, androgen receptor signaling, nuclear receptor binding and upstream regulatory components. These endocrine-associated gene subsets were then used to generate focused PPI networks, which were visualized interactively using visNetwork (v2.1.2) [33]. Each PPI subnetwork was generated by filtering the STRING interactome using a specific GO biological process as the entry point (e.g., estrogen metabolic process Nodes were colored according to their Gene Ontology (GO) biological process annotations using the RColorBrewer package (v1.1-3); nodes sharing the same GO term were displayed in the same color. As proteins may be associated with multiple GO terms, node coloring is intended as a visual aid and does not imply exclusive functional assignment. Although each subnetwork is centered on a focal GO term, it also includes proteins annotated to other GO categories that are functionally connected through high-confidence interactions. A STRING combined-score cutoff of 0.4 (“medium confidence”) was chosen to balance biological coverage with network interpretability. In endocrine signaling, functionally relevant interactions frequently occur through transient, context-dependent, and literature-supported regulatory links (e.g., receptor–cofactor complexes, transcriptional regulators, and pathway crosstalk) that may be lost under a stringent ≥0.7 (“high confidence”) threshold, especially when restricting analyses to GO-filtered gene subsets. The 0.4 cutoff threshold was selected *a priori* to balance interaction reliability with network connectivity, particularly in the context of endocrine signaling pathways, where biologically relevant interactions often involve transient, context-dependent, or literature-supported regulatory associations that may not meet higher confidence thresholds. More stringent cutoffs (e.g., ≥0.7) were evaluated during preliminary analyses but resulted in excessive network sparsity when applied to Gene Ontology-filtered gene sets, limiting the ability to identify coherent modules and pathway bridges. The 0.4 cutoff was therefore chosen to preserve a biologically interpretable network structure while excluding low-support interactions, facilitating hypothesis generation without overinflating connectivity. For transparency and robustness assessment, representative subnetworks generated using a higher STRING confidence threshold (≥0.7) are provided in the Supplementary Material. These higher-confidence networks retain the same core nodes identified at the 0.4 threshold but show reduced connectivity due to the loss of intermediate linking interactions.

## 3. Results

### 3.1. Differential Expression Overview

**Figure 1:**
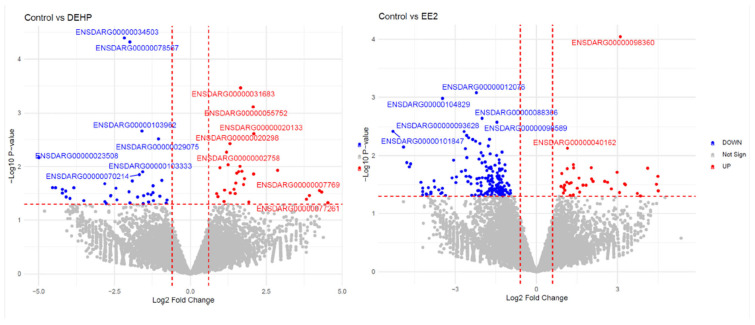
Differential gene expression in zebrafish embryos following DEHP or EE2 exposure. Volcano plots show transcriptomic responses in zebrafish embryos exposed from 96 to −120 h post-fertilization (hpf) to di(2-ethylhexyl) phthalate (DEHP; left) or 17-α-ethinylestradiol (EE2; right), relative to ethanol-treated controls. The x-axis represents log2 fold change, and the y-axis shows −log10-adjusted *p*-values. Red points indicate significantly up-regulated genes, and blue points indicate significantly down-regulated genes, based on thresholds of |log2 fold change| ≥ 0.3 and FDR ≤ 0.1 (indicated by dashed vertical and horizontal lines). Gray points represent genes not meeting the significance criteria. Selected differentially expressed genes are labeled by Ensembl gene ID. These plots illustrate a modest but detectable transcriptional response to both exposures, with a greater number of differentially expressed genes observed following EE2 compared to DEHP exposure.

Initial analysis of embryonic transcriptomes revealed treatment-associated changes in gene expression; however, the overall magnitude of differential expression was modest. To balance sensitivity to subtle regulatory responses during embryonic development with statistical rigor, differential expression analysis was performed using a false discovery rate (FDR) threshold of ≤ 0.1. This relatively permissive threshold was selected to capture biologically meaningful transcriptional changes during embryogenesis, a developmental window characterized by tightly regulated and often moderate shifts in gene expression that can nonetheless have lasting functional consequences. Using these criteria, exposure to EE2 resulted in 575 differentially expressed genes, whereas DEHP exposure was associated with 267 differentially expressed genes (Figure 1). The smaller number of DEGs observed following DEHP exposure compared to EE2 suggests a weaker or more targeted transcriptional response in embryos under the exposure conditions tested. Overall, these results indicate that DEHP exposure induces detectable but limited alterations in the embryonic transcriptome, consistent with subtle modulation rather than broad transcriptional reprogramming during early development.

The selected thresholds were chosen to maximize sensitivity in an exploratory, low-dose DEHP context where large fold changes are not expected; however, while this approach increases sensitivity to low-amplitude changes, it reduces stringency at the individual gene level and increases the likelihood of false positives, meaning the results are best interpreted as hypothesis-generating and are more robustly evaluated at the convergent pathway- and network-level rather than as definitive isolated gene effects. Given the limited number of genes exceeding gene-level significance thresholds and the modest effect sizes observed, we thus next employed pathway-level and network-based analyses to determine whether coordinated transcriptional shifts were present across biologically related gene sets. Such approaches are well suited to detecting subtle but functionally coherent responses that may not be apparent from differential expression analysis alone, particularly under environmentally relevant exposure conditions where large-amplitude transcriptional changes are not expected.

### 3.2. KEGG Pathway Enrichment Analysis

**Figure 2:**
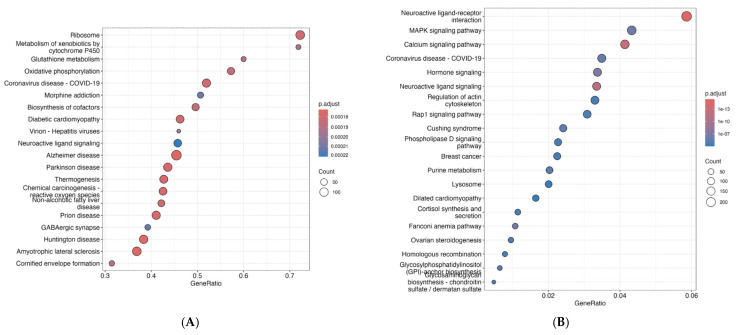
KEGG pathway enrichment highlights coordinated signaling, cytoskeletal, and neurobiological responses to exposure. Dot plots showing KEGG pathway enrichment results derived from differential gene expression analysis. (**A**) Over-representation analysis (ORA) of differentially expressed genes identifies significant enrichment of pathways involved in signal transduction and cellular processes, including MAPK signaling, regulation of the actin cytoskeleton, neuroactive ligand–receptor interaction, and pathways associated with neurodegeneration. (**B**) Gene set enrichment analysis (GSEA) reveals coordinated enrichment of pathways related to neurodegenerative disease, intracellular signaling, and metabolism, reflecting shared molecular processes such as mitochondrial function, proteostasis, oxidative stress response, and cytoskeletal integrity rather than disease-specific mechanisms. In both panels, the x-axis represents the gene ratio (proportion of pathway genes represented), dot size corresponds to the number of genes contributing to each pathway, and dot color indicates the adjusted *p*-value (FDR). Together, these analyses demonstrate broad, pathway-level transcriptional remodeling characterized by integrated effects on signaling cascades, cellular structure, and neurodevelopmentally relevant processes rather than isolated gene-level perturbations.

KEGG pathway enrichment analysis revealed broad and highly significant perturbations across signaling, cellular, and organismal systems, indicating coordinated transcriptional responses rather than isolated gene-level effects. Over-representation analysis (ORA) identified strong enrichment of pathways involved in signal transduction and cellular dynamics, with the MAPK signaling pathway emerging as one of the most significantly enriched pathways (adjusted *p* < 10^−14^), as shown in Figure 2A and Supplementary Table S1. This pathway encompassed a large proportion of differentially expressed genes and included multiple kinases and transcriptional regulators implicated in stress responses, proliferation, and developmental signaling.

Pathways associated with cytoskeletal organization and cell motility, particularly regulation of the actin cytoskeleton, were also prominently enriched, suggesting alterations in cellular architecture and morphogenetic processes. Consistent with these findings, pathways related to axon guidance and neuroactive ligand–receptor interaction were significantly over-represented, indicating potential impacts on neurodevelopmental patterning, synaptic signaling, and neural connectivity. These results align with enrichment observed in broader nervous system-related pathways, including “pathways of neurodegeneration–multiple diseases,” reflecting convergence on mitochondrial, proteostatic, and stress-response mechanisms shared across neurological disorders. Enrichment of neurodegeneration-associated KEGG pathways reflects shared cellular stress, proteostasis, and metabolic gene networks rather than neurodegenerative processes per se and is therefore best interpreted as an indicator of convergent stress-related transcriptional responses.

At a higher level of biological organization, enriched KEGG categories spanned environmental information processing, cellular processes, and organismal systems, highlighting integrated effects on signaling cascades, intracellular structure, and developmental programs. Many enriched pathways exhibited moderate fold enrichment but high gene counts and strong statistical support, consistent with coordinated, pathway-wide transcriptional shifts rather than reliance on a small number of highly dysregulated genes.

### 3.3. Gene Set Enrichment Analysis (GSEA)

Among the most strongly enriched pathways were those associated with neurodegenerative diseases, all of which exhibited significant negative normalized enrichment scores (NES ≈ −1.7 to −2.1; FDR *q* < 0.01). Although annotated as disease pathways, these gene sets are enriched for shared molecular processes rather than disease-specific mechanisms, reflecting common disruptions in mitochondrial function, proteostasis, oxidative stress responses, and cytoskeletal integrity (Figure 2B, Supplemental Table S1).

Core enrichment genes contributing to these pathways included multiple proteasome subunits, mitochondrial respiratory chain components, cytoskeletal proteins, and stress-response regulators, indicating coordinated downregulation of pathways involved in cellular energy metabolism, protein turnover, and redox homeostasis. The consistent enrichment patterns across multiple neurodegeneration-related pathways suggest convergence on conserved cellular stress mechanisms rather than activation of discrete pathological programs. Beyond neurodegeneration-associated pathways, GSEA also identified enrichment of pathways related to cellular metabolism, intracellular transport, and signaling, further supporting broad remodeling of fundamental cellular processes. Importantly, these pathways were detected by GSEA despite not necessarily being driven by a small subset of highly differentially expressed genes, underscoring the value of rank-based enrichment approaches for identifying subtle but biologically coherent transcriptional responses.

The leading-edge subsets of enriched pathways accounted for a substantial proportion of pathway genes and were distributed throughout the ranked gene list, consistent with coordinated, pathway-wide expression shifts rather than isolated gene effects. These findings complement the over-representation analysis results and reinforce the conclusion that exposure induces integrated alterations in signaling, metabolic, and neurobiological pathways. Collectively, the GSEA results demonstrate that the transcriptional response is characterized by systematic downregulation of pathways governing mitochondrial function, proteostasis, and neuronal integrity. These pathway-level changes provide mechanistic insight into how molecular perturbations may translate into higher-order functional outcomes and support the interpretation of the observed transcriptional profile as reflective of coordinated cellular stress and neurodevelopmentally relevant responses.

### 3.4. Estrogen Metabolic Process Subnetwork

**Figure 3:**
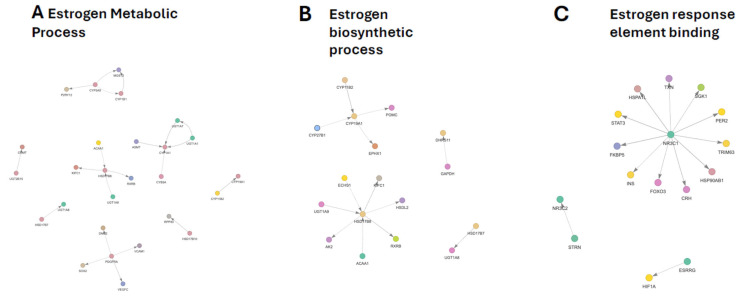
Estrogen-associated protein–protein interaction subnetworks. (**A**) Estrogen metabolism-associated PPI subnetwork generated using the Gene Ontology (GO) term estrogen metabolic process as the entry point. (**B**) Estrogen biosynthetic process subnetwork. (**C**) Estrogen response element binding subnetwork. Edges represent STRING-derived interactions retained at a combined-score cutoff of 0.4 (medium confidence) to preserve biologically meaningful connectivity while limiting network sparsity. Nodes are colored according to GO biological process annotations; shared colors indicate common GO terms, and node coloring is intended as a visual aid rather than an exclusive functional assignment.

The GO-filtered PPI subnetwork associated with the estrogen metabolic process revealed coordinated modulation of pathways involved in estrogen biosynthesis, interconversion, and detoxification (Figure 3A). In the main network, enzymes central to steroidogenesis and estrogen turnover formed a tightly connected module, indicating concerted regulation rather than isolated gene effects.

At the pathway level, the network suggested a potential reduction in local estrogen synthesis during early embryogenesis, driven by altered regulation of key steroidogenic enzymes, alongside compensatory shifts in downstream interconversion and clearance mechanisms. The 17β-hydroxysteroid dehydrogenase module showed mixed regulation, consistent with changes in the balance between active and inactive estrogens and androgens. These enzymes were embedded within a broader metabolic context that included lipid metabolism, redox regulation, and mitochondrial function, highlighting integration between steroid metabolism and cellular energy states.

Estrogen-metabolizing cytochrome P450 enzymes and associated redox partners exhibited heterogeneous responses, indicating selective remodeling of oxidative estrogen metabolism rather than uniform activation or suppression. This cluster also intersected glutathione-dependent detoxification and indole-related metabolic pathways, suggesting crosstalk between estrogen metabolism, redox homeostasis, and ancillary metabolic processes.

Conjugation pathways represented another prominent feature of the subnetwork. UDP-glucuronosyltransferases displayed divergent regulation, consistent with reorganization of estrogen conjugation and clearance capacity. These enzymes were linked within the PPI network to signaling-associated nodes, reflecting functional proximity captured by STRING rather than direct enzymatic interactions.

Additional interacting nodes not directly annotated to estrogen metabolism—including growth factor, vascular, adhesion, and cytoskeletal regulators—were positioned at the periphery of the network. Their presence suggests broader regulatory coupling between metabolic state and developmental signaling pathways. Finally, catechol estrogen methylation was represented through linkage between methyltransferase and glucuronidation modules, reinforcing the coordinated regulation of estrogen detoxification routes. Comprehensive gene-level fold changes and full network annotations for estrogen- and androgen-associated pathways are provided in Supplementary Files S1 and S2 respectively.

### 3.5. Estrogen Biosynthetic Process Subnetwork

The GO-filtered PPI subnetwork associated with the *estrogen biosynthetic process* was dominated by core steroidogenic enzymes and their functionally linked interactors (Figure 3B). This network partially overlapped with the estrogen metabolic process, reflecting shared regulation of upstream biosynthesis and downstream estrogen handling.

At the pathway level, the network was centrally organized around aromatase, indicating that modulation of this rate-limiting step represents a major regulatory feature of the biosynthetic program. Reduced aromatase expression suggests an overall attenuation of estrogen production capacity during early embryogenesis, with additional steroidogenic enzymes positioned as secondary contributors within the same module.

Hydroxysteroid dehydrogenases within the network showed modest and bidirectional changes, consistent with fine-tuning rather than wholesale activation or suppression of estrogen biosynthesis. These enzymes formed connections with mitochondrial and peroxisomal metabolic nodes, linking estrogen production to lipid β-oxidation, redox balance, and cellular energy homeostasis.

Conjugation and nuclear receptor-associated interactors were embedded within the biosynthetic module, reinforcing coordination between estrogen synthesis, intracellular signaling, and hormone turnover. The presence of retinoid receptor components and glucuronidation enzymes further suggests integration of estrogen biosynthesis with transcriptional control and detoxification pathways.

Peripheral associations with glycolytic and mitochondrial energy-related proteins point to broader metabolic coupling, in which estrogen biosynthesis is aligned with cellular energetic state rather than operating as an isolated pathway. Together, these features indicate that estrogen biosynthesis is regulated as part of an integrated metabolic network; detailed gene-level alterations and full network annotations can be found in Supplementary Files S1 and S2 respectively.

### 3.6. Estrogen Response Element Binding Subnetwork

The GO-filtered PPI subnetwork associated with estrogen response element binding comprised nuclear receptors and scaffolding proteins central to hormone-responsive transcription, embedded within a broader network of signaling, stress-response, and metabolic regulators (Figure 3C). Annotated core nodes included steroid and estrogen-related nuclear receptors, while additional interacting proteins reflected functional integration with diverse cellular regulatory pathways.

At the network level, glucocorticoid and mineralocorticoid receptor signaling emerged as dominant features of this subnetwork. Receptor-associated chaperones and co-regulators formed a cohesive module, consistent with coordinated regulation of receptor maturation, sensitivity, and transcriptional activity. Downstream effectors linked this module to metabolic adaptation and stress-responsive signaling, indicating crosstalk between hormone-dependent transcription and cellular homeostasis.

Transcription factors positioned downstream of steroid receptors connected estrogen response element binding to inflammatory, stress, and developmental pathways, reinforcing the role of this network as a hub for hormonal signal integration rather than a strictly estrogen-specific module. Circadian and neuroendocrine regulators were also embedded within the subnetwork, suggesting temporal and systemic modulation of hormone-responsive gene expression.

Metabolic and redox-associated interactors further linked estrogen response element binding to cellular energetic and oxidative states, consistent with known modulation of nuclear receptor activity by metabolic cues. In parallel, a mineralocorticoid receptor-centered node, together with its scaffolding components, indicated coordinated regulation across corticosteroid receptor families.

Finally, peripheral interactions between estrogen-related nuclear receptors and hypoxia-associated regulators connected estrogen-responsive transcription to metabolic and oxygen-sensing pathways. Collectively, these features indicate that estrogen response element binding is organized as part of an integrated signaling network that coordinates hormone responsiveness with stress, metabolic, and circadian regulation. Full network composition and differential expression data are provided in Supplementary Files S1 and S2 respectively.

### 3.7. Estrogen Receptor Signaling Pathway Subnetwork

**Figure 4:**
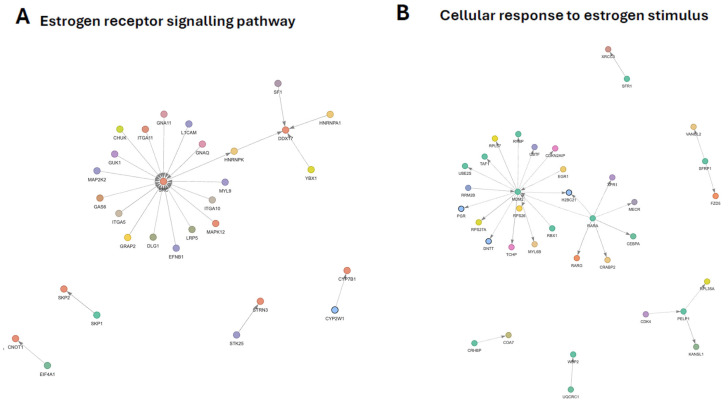
Estrogen signaling-associated protein–protein interaction subnetworks. (**A**) Estrogen receptor signaling pathway subnetwork. (**B**) Cellular response to estrogen stimulus subnetwork. Protein–protein interaction networks were generated using the corresponding Gene Ontology (GO) biological process terms as entry points. Edges indicate STRING interactions (combined score ≥ 0.4), and node colors denote GO biological process annotations.

The GO-filtered PPI subnetwork associated with the estrogen receptor signaling pathway comprised a core set of proteins directly annotated to this process, embedded within a broader interaction landscape spanning kinase signaling, cell adhesion and migration, RNA processing, chromatin regulation, protein turnover, and metabolic pathways (Figure 4A). This organization reflects the integration of estrogen receptor (ER) signaling into multiple cellular regulatory systems.

At the network level, the subnetwork was dominated by a kinase-centered module organized around SRC, consistent with its established role in coordinating both genomic and non-genomic ER signaling. Although SRC transcript levels were unchanged, its position as the primary interaction hub underscores the dependence of ER activity on kinase signaling, adhesion complexes, and associated scaffolding networks rather than on transcriptional regulation of SRC itself. This module was strongly enriched for adhesion- and migration-related components and upstream activators, highlighting a signaling environment characteristic of SRC-driven, membrane-associated ER activation.

Kinase cascades associated with MAPK and G-protein signaling formed an interconnected layer within this SRC-centered module, indicating engagement of phosphorylation-dependent pathways that amplify or modulate ER signaling output. Components of the STRIPAK complex were also present, suggesting parallel regulation of cytoskeletal organization and cellular polarity in conjunction with ER-linked kinase activity.

A second prominent module was organized around the ER co-regulator DDX17 and consisted primarily of RNA-binding proteins and chromatin-associated factors. This cluster represents post-transcriptional and chromatin-level mechanisms that fine-tune ER-dependent gene expression, linking estrogen signaling to mRNA processing, translation, and transcriptional control. Protein turnover pathways were also embedded within this module, consistent with dynamic cycling of ER cofactors and transcriptional regulators.

Peripheral branches of the network included enzymes involved in steroid and xenobiotic metabolism, suggesting potential indirect modulation of ER signaling through alternative ligand processing routes. Together, these features indicate that estrogen receptor signaling is structured as a multi-layered network integrating kinase-driven signaling, transcriptional and post-transcriptional regulation, cytoskeletal dynamics, and metabolic context. Detailed gene-level changes and full network annotations are provided in Supplementary Files S1 and S2 respectively.

### 3.8. Cellular Response to Estrogen Stimulus Subnetwork

The GO-filtered PPI subnetwork associated with the cellular response to estrogen stimulus comprised a set of estrogen-responsive regulators embedded within broader networks controlling transcription, protein turnover, chromatin organization, mitochondrial metabolism, cell-cycle progression, and developmental signaling (Figure 4B). Core annotated nodes were distributed across two dominant regulatory modules, indicating that estrogen responsiveness is organized through parallel but interconnected pathways.

At the network level, one major module was organized around MDM2, which emerged as the primary interaction hub. This module linked estrogen-responsive signaling to ubiquitin–proteasome activity, ribosome biogenesis, DNA damage responses, and p53-associated regulatory circuits. The dense connectivity of this cluster suggests that estrogen signaling intersects with cellular stress surveillance, protein quality control, and transcriptional reprogramming rather than acting solely through canonical receptor-mediated transcription.

Chromatin-associated and transcriptional regulators were embedded within the MDM2-centered module, positioning estrogen responsiveness within early transcriptional activation and chromatin remodeling processes. Connections to steroid-responsive transcription factors further situate this module within a broader hormone-regulated transcriptional cycle.

A second major module was anchored by the retinoic acid receptor RARA and associated co-regulatory proteins. This cluster linked estrogen-responsive signaling to retinoid-dependent chromatin remodeling, metabolic regulation, mitochondrial function, and cell-cycle control. Interactions involving estrogen receptor coactivators provided a mechanistic bridge between retinoid and estrogen signaling pathways, reinforcing coordinated regulation across nuclear receptor systems.

Additional peripheral branches of the network included mitochondrial respiratory components and developmental signaling pathways. Notably, a Wnt/planar cell polarity-associated module showed coordinated downregulation, consistent with modulation of developmental patterning pathways in response to estrogenic cues. DNA repair-associated interactors were also present, suggesting that genome stability mechanisms are integrated into the cellular response to estrogen stimulus.

Together, these features indicate that the cellular response to estrogen stimulus is structured as a multi-hub network integrating hormone-dependent transcription, proteostasis, metabolic state, cell-cycle regulation, and developmental signaling. Detailed gene-level changes and full network annotations are provided in Supplementary Files S1 and S2 respectively.

### 3.9. Androgen Metabolic Process Subnetwork

**Figure 5:**
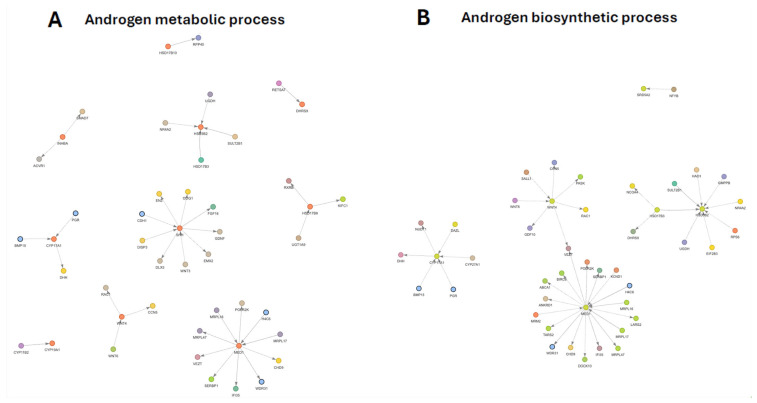
Androgen-associated protein–protein interaction subnetworks. (**A**) Androgen metabolic process subnetwork. (**B**) Androgen biosynthetic process subnetwork. Protein–protein interaction networks were generated using the corresponding Gene Ontology (GO) biological process terms as entry points. Edges represent STRING-derived interactions (combined score ≥ 0.4), and node colors indicate GO biological process annotations.

The GO-filtered PPI subnetwork associated with the androgen metabolic process comprised enzymes directly involved in androgen biosynthesis and modification, together with regulatory and developmental interactors that embed androgen metabolism within broader transcriptional, mitochondrial, and signaling contexts (Figure 5A). Core annotated nodes formed a cohesive metabolic module, while additional high-confidence interactors reflect functional integration with hormone signaling and developmental pathways.

At the pathway level, the subnetwork revealed coordinated modulation of short-chain dehydrogenases and cytochrome P450 enzymes that collectively define androgen biosynthetic flux. Differential regulation within this module suggests a redistribution of precursor supply, reductive capacity, and downstream steroid interconversion rather than uniform up- or down-regulation of androgen production. Phase II conjugation enzymes were embedded within the same interaction neighborhood, indicating coupling between androgen biosynthesis and metabolic clearance.

Cytochrome P450-centered interactions positioned androgen metabolism in direct continuity with estrogen and mineralocorticoid pathways, highlighting shared enzymatic nodes that mediate androgen–estrogen interconversion and broader steroidogenic balance. Retinol- and steroid-associated oxidoreductases formed an auxiliary module adjacent to the core steroidogenic enzymes, further linking androgen metabolism to redox and vitamin A-related processes.

Beyond metabolic enzymes, the subnetwork incorporated a prominent set of developmental regulators, including Wnt and Hedgehog signaling components, which formed an interconnected module associated with morphogenetic patterning and reproductive tract development. The integration of these signaling pathways suggests that changes in androgen metabolism are closely aligned with developmental signaling programs rather than acting in isolation.

A distinct regulatory cluster centered on nuclear receptor coactivation connected androgen metabolism to transcriptional control, chromatin regulation, and mitochondrial ribosomal components. This organization indicates coordination between androgen-responsive transcription and mitochondrial translational capacity, reinforcing the integration of steroid signaling with cellular energy and biosynthetic states.

Together, these features indicate that androgen metabolism is organized as a multi-module network linking steroidogenic flux, conjugation and clearance; nuclear receptor coactivation; and developmental signaling pathways. Detailed gene-level changes and complete network annotations are provided in Supplementary Files S1 and S2 respectively.

### 3.10. Androgen Biosynthetic Process Subnetwork

The GO-filtered PPI subnetwork associated with the androgen biosynthetic process was organized around a core set of steroidogenic enzymes, nuclear co-regulators, and developmental signaling components (Figure 5B). Core annotated nodes were embedded within a broader interaction landscape linking androgen biosynthesis to transcriptional control, mitochondrial function, and morphogenetic signaling.

At the pathway level, the network revealed coordinated downregulation of key enzymes responsible for early androgen precursor formation, consistent with attenuation of androgen biosynthetic capacity. Central steroidogenic steps formed the backbone of the subnetwork, with downstream enzymes positioned in a manner suggesting partial compensatory regulation rather than uniform suppression of androgen production. Phase II modification and redox-related enzymes were integrated within this core module, indicating coupling between androgen biosynthesis, metabolic buffering, and hormone availability.

A second prominent cluster linked androgen biosynthetic enzymes to mitochondrial and germ cell-associated regulators, highlighting coordination between steroidogenesis, mitochondrial metabolism, and gametogenic processes. The presence of oxidative stress-protective components within this module further suggests that androgen biosynthesis is embedded within broader mitochondrial quality-control and redox-regulatory programs.

Regulatory organization of the subnetwork was further shaped by a nuclear receptor coactivator-centered module that connected androgen biosynthesis to chromatin regulation, basal transcriptional machinery, and mitochondrial translation. This architecture indicates that modulation of androgen output is accompanied by reprogramming of the transcriptional and metabolic environment supporting steroid-responsive gene expression, even in the absence of large changes in coactivator transcript levels.

In parallel, a distinct developmental signaling module dominated by Wnt pathway components was strongly represented. The integration of Wnt signaling with core steroidogenic enzymes places androgen biosynthesis within a developmental framework associated with gonadal differentiation and patterning. This organization suggests that perturbation of androgen biosynthetic capacity is closely linked to alterations in the developmental context in which androgens exert their effects.

Collectively, these features indicate that androgen biosynthesis is regulated as a multi-module network integrating steroidogenic flux, mitochondrial and transcriptional capacity, and developmental signaling pathways. Detailed gene-level changes and full network annotations are provided in Supplementary Files S1 and S2 respectively.

### 3.11. Androgen Receptor Signaling Pathway Subnetwork

**Figure 6:**
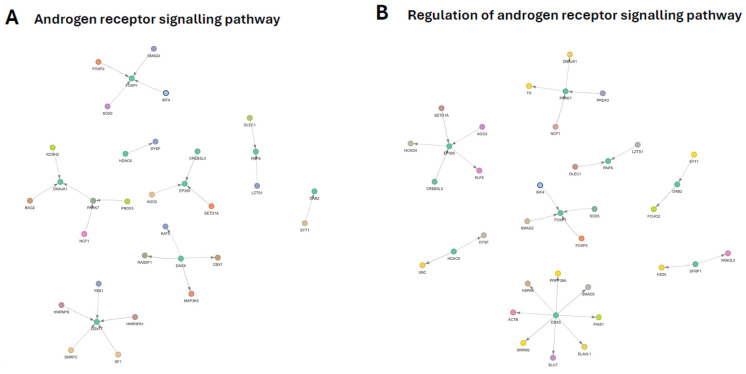
Androgen signaling-associated protein–protein interaction subnetworks. (**A**) Androgen receptor signaling pathway subnetwork. (**B**) Regulation of androgen receptor signaling pathway subnetwork. Protein–protein interaction networks were generated using the corresponding Gene Ontology (GO) biological process terms as entry points. Edges correspond to STRING interactions retained at a combined-score threshold of ≥0.4, while node colors reflect GO biological process annotations.

The GO-filtered PPI subnetwork associated with regulation of androgen receptor (AR) signaling comprised a core set of AR-associated transcriptional regulators embedded within a broader interaction landscape involving RNA processing, chromatin modification, proteostasis, vesicle trafficking, and stress-response pathways (Figure 6A). This organization highlights the integration of AR regulation within multiple layers of cellular control.

At the network level, coordinated reorganization of transcriptional co-regulators and RNA-processing machinery emerged as a dominant feature. A central module linked AR regulation to RNA splicing, transcript stability, and coactivator function, suggesting that post-transcriptional control represents a key regulatory layer influencing AR transcriptional output. These interactions indicate reduced efficiency or altered composition of the splicing and coactivation environment supporting AR-responsive gene expression.

A second major module was centered on chromatin and enhancer-associated regulators, implicating acetylation- and methylation-dependent mechanisms in reshaping AR-driven transcription. Integration of transcription factors and RNA silencing components within this cluster further supports reprogramming of chromatin accessibility and transcript fine-tuning as part of AR signaling regulation.

Differential modulation of transcription factors with known roles in steroid receptor signaling suggests a shift in nuclear regulatory balance rather than uniform activation or repression. These changes are consistent with altered responsiveness of AR target genes to hormonal and developmental cues.

In parallel, a prominent proteostasis-oriented module linked AR regulation to chaperone activity, protein folding, and oxidative stress management. This cluster indicates enhanced engagement of cytoplasmic quality-control mechanisms that influence AR stability, maturation, and intracellular trafficking. Connections to redox-associated proteins further suggest that metabolic and oxidative states contribute to AR modulation.

Finally, the subnetwork incorporated a downregulated Wnt/planar cell polarity-associated module and endocytic regulators, reinforcing known crosstalk between Wnt signaling, membrane trafficking, and androgen receptor activity. Together, these features indicate that regulation of AR signaling is organized as a multi-module network integrating transcriptional, post-transcriptional, proteostatic, and signaling processes. Detailed gene-level changes and complete network annotations are provided in Supplementary Files S1 and S2 respectively.

### 3.12. Regulation of Androgen Receptor Signaling Pathway Subnetwork

The GO-filtered PPI subnetwork associated with regulation of androgen receptor (AR) signaling comprised core AR-associated regulators embedded within a broader interaction landscape involving RNA processing, chromatin modification, proteostasis, vesicle trafficking, and stress-response pathways (Figure 6B). This organization underscores the multi-layered regulation of AR signaling beyond direct receptor–ligand interactions.

At the network level, a coordinated reorganization of transcriptional co-regulators and RNA-processing machinery emerged as a dominant feature. A central module linked AR regulation to splicing factors, RNA-binding proteins, and coactivator-associated components, indicating that post-transcriptional control and coactivator availability represent key regulatory layers influencing AR transcriptional output. The structure of this module suggests reduced efficiency or altered composition of the RNA-processing environment supporting androgen-responsive gene expression.

A second major module was organized around chromatin- and enhancer-associated regulators, implicating acetylation- and methylation-dependent mechanisms in reshaping AR-driven transcription. Integration of transcription factors and RNA silencing components within this cluster supports reprogramming of chromatin accessibility and enhancer activity as part of AR signaling regulation.

Differential modulation of transcription factors with established roles in steroid receptor biology further suggests a shift in nuclear regulatory balance rather than uniform activation or repression of AR signaling. These changes are consistent with altered sensitivity of AR target genes to hormonal and developmental cues.

In parallel, a prominent proteostasis-oriented module linked AR regulation to chaperone activity, protein folding, and oxidative stress management. This cluster indicates enhanced engagement of cytoplasmic quality-control mechanisms that influence AR stability, maturation, and intracellular trafficking. Connections to redox-associated regulators further suggest that metabolic and oxidative states contribute to AR modulation.

Finally, the subnetwork incorporated a downregulated Wnt/planar cell polarity-associated module together with endocytic regulators, reinforcing known crosstalk between Wnt signaling, membrane trafficking, and androgen receptor activity. Collectively, these features indicate that regulation of AR signaling is organized as an integrated, multi-module network coordinating transcriptional, post-transcriptional, proteostatic, and signaling processes. Full network annotations and gene-level changes are provided in Supplementary Files S1 and S2 respectively.

### 3.13. Nuclear Androgen Receptor (AR) Binding Subnetwork

**Figure 7:**
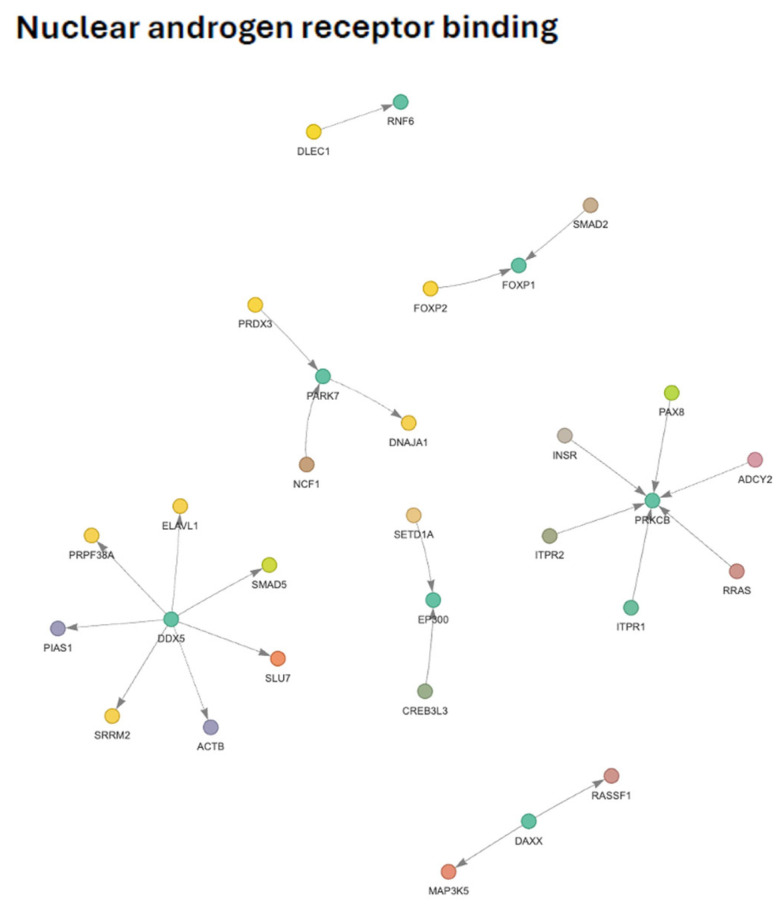
Nuclear androgen receptor binding-associated protein–protein interaction subnetwork. Protein–protein interaction network generated using the Gene Ontology (GO) term nuclear androgen receptor binding as the entry point. Edges denote STRING interactions (≥ 0.4), and node colors denote GO biological process annotations.

The GO-filtered PPI subnetwork associated with *nuclear androgen receptor (AR) binding* comprised AR-associated co-regulators embedded within a broader network of transcriptional, chromatin-remodeling, RNA-processing, and nuclear proteostasis factors (Figure 7). This subnetwork partially overlapped with the regulation of androgen receptor signaling pathway, reflecting continuity between upstream regulatory mechanisms and nuclear AR engagement.

At the network level, two principal regulatory modules were evident, representing distinct mechanistic layers of AR nuclear function. One module was organized around RNA-processing and coactivator-associated factors and linked AR binding to splicing regulation, transcript stability, and nuclear architecture. The structure of this module suggests attenuation or reorganization of AR-associated RNA-processing capacity, a process essential for efficient AR-dependent transcriptional output.

The second major module was centered on chromatin remodeling and histone modification. This cluster integrated histone acetylation and methylation activities with transcription factor networks, indicating reshaping of chromatin accessibility and coactivator balance at AR-responsive loci. The configuration of this module supports altered enhancer and promoter engagement rather than uniform suppression of AR chromatin binding.

Proteostasis and redox-support mechanisms formed an additional cohesive layer within the subnetwork. Chaperone and redox-associated components linked AR nuclear binding to protein folding, oxidative stress management, and mitochondrial redox balance, consistent with known requirements for AR stability and nuclear retention under stress conditions.

In contrast, a signaling-oriented module associated with kinase- and calcium-dependent pathways showed coordinated downregulation. This organization suggests impaired upstream signaling inputs that normally facilitate AR phosphorylation, nuclear localization, and activation cycles. The integration of metabolic and growth factor signaling components within this module further indicates that AR nuclear binding is sensitive to broader cellular signaling states.

Finally, differential modulation of forkhead transcription factors within the network suggests a shift in nuclear transcriptional programs governing AR specificity and output. Together, these features indicate that nuclear AR binding is regulated as a multi-layered network integrating RNA processing, chromatin remodeling, proteostasis, and signal-dependent activation. Full network annotations and gene-level changes are provided in Supplementary Files S1 and S2 respectively.

## 4. Discussion

Prior work strongly supports the use of zebrafish as an integrated model for identifying endocrine-disrupting chemical (EDC) activity and elucidating underlying mechanisms, particularly when combined with genome-wide approaches such as RNA-seq [17]. Transcriptomic profiling in zebrafish enables sensitive, whole-organism detection of endocrine perturbations and facilitates pathway-level inference that extends beyond traditional targeted assays [18,34]. These molecular strengths are complemented by practical advantages of the model, including optical transparency, rapid *ex utero* development, and suitability for high-throughput experimentation, which together allow direct linkage of transcriptional responses with organismal phenotypes [35,36,37,38,39,40].

We note that RNA-seq analyses in this study were performed on pooled whole larvae, and therefore observed transcriptional changes cannot be attributed to specific organs, endocrine glands, or cell types. Accordingly, enrichment of endocrine-related pathways reflects integrated, organism-level transcriptional signatures rather than tissue- or cell-type-specific activity.

Interpretation of androgen- and estrogen-associated pathway enrichment must be considered in the context of the developmental stage examined. The 96–120 hpf interval represents a transitional phase in zebrafish development in which endocrine-related pathways are being established rather than operating as fully functional adult endocrine axes. During this window, components of steroidogenesis, nuclear receptor co-regulation, chromatin remodeling, and hormone-responsive transcriptional machinery are actively developing and integrating with broader metabolic and stress-responsive systems. Consequently, enrichment of estrogen- and androgen-associated pathways observed here should not be interpreted as evidence of endocrine axis disruption or altered hormonal function. Instead, these findings are best viewed as transcriptomic and network-level signatures reflecting modulation of developing steroid metabolic and receptor-linked regulatory frameworks, as well as broader coupling with cellular metabolism and stress-response pathways. Importantly, the present analyses do not directly assess hormone levels, receptor activation, or physiological endocrine outputs and therefore remain limited to developmental transcriptional associations.

Mechanistic relevance is further supported by in vitro evidence demonstrating that plasticizers such as DEHP can disrupt estrogen and thyroid hormone signaling, including antagonism of estradiol-induced estrogen receptor (ER) transactivation and complex, mixture-dependent effects on thyroid-related endpoints [41]. In vivo, hormone-responsive transgenic zebrafish lines, such as estrogen-responsive ERE-GFP reporters, have provided spatially resolved visualization of ER-mediated transcription, establishing zebrafish larvae as sensitive sentinels for ER-active compounds [34,42].

Consistent with these endocrine-disruptive properties, developmental DEHP exposure has been shown to impair larval locomotor behavior and alter dopaminergic signaling, accompanied by increased apoptosis and oxidative stress, linking molecular stress pathways with functional neurobehavioral outcomes [43]. At later life stages, environmentally relevant DEHP exposures disrupt hepatic lipid and energy metabolism in adult males, affecting fatty-acid and ketone body pathways and key metabolic transcriptional networks, with transcriptional signatures that partially overlap yet remain distinct from those induced by potent estrogens [18]. In addition, alterations in thyroid hormone levels and hypothalamic–pituitary–thyroid axis-related gene expression in DEHP-exposed larvae further support thyroid endocrine toxicity as a relevant mode of action [44].

Collectively, these studies provide a strong contextual framework for interpreting transcriptomic and phenotypic effects of phthalates in zebrafish, highlighting the model’s utility for capturing endocrine, metabolic, and neurodevelopmental consequences across levels of biological organization. Notably, however, several published zebrafish transcriptomic studies have employed DEHP concentrations that extend into the high-dose range, potentially limiting physiological relevance [15,16]. In contrast, the exposure concentration used in the present study was selected to reflect environmentally and physiologically relevant conditions, strengthening the interpretability of observed endocrine-associated transcriptional signatures during early embryogenesis.

The present study focuses exclusively on embryo-stage exposure in the 96–120 h post-fertilization (hpf) window, which is a critical developmental transition in zebrafish because it marks the shift from organ patterning to functional maturation and integration of organ systems. By ~96 hpf (≈4 dpf), most major organs, including the pancreas, liver, heart, intestine, and nervous system, are structurally formed, while between 96 and 120 hpf, these organs undergo rapid differentiation, metabolic activation, and establishment of physiological function. This period also coincides with yolk depletion and the onset of exogenous feeding, making larvae increasingly reliant on endogenous metabolic and endocrine regulation. Neurobehavioral circuits mature substantially during this window, enabling robust locomotor activity and sensory processing, which is why functional phenotypes (e.g., swimming behavior and visual responses) become readily quantifiable [35]. Consequently, perturbations during 96–120 hpf can reveal latent or functional toxic effects, such as altered metabolism, neurodevelopmental deficits, or endocrine-associated transcriptional responses, that may not be evident during earlier morphogenetic stages, making this interval especially informative for developmental toxicology and transcriptomic studies [45].

In this study, 17α-ethinylestradiol (EE2) was included as a positive control to benchmark the sensitivity of the experimental design and transcriptomic analyses to estrogen-responsive gene regulation. EE2 was included as a direct estrogen receptor agonist to benchmark transcriptional sensitivity, whereas DEHP was expected to act through indirect and multi-pathway mechanisms, and comparisons between their responses are therefore qualitative and contextual rather than indicative of relative potency or strength. As expected, EE2 elicited a more pronounced transcriptional response than DEHP, reflecting its role as a potent estrogen receptor agonist rather than a directly comparable environmental exposure. Accordingly, the EE2-associated expression changes serve primarily to validate assay responsiveness and analytical sensitivity, and differences in response magnitude between EE2 and DEHP should be interpreted in light of this experimental context rather than as evidence of relative biological potency.

Although the gene-level expression changes observed in this study are modest in magnitude, such patterns are consistent with low-dose, environmentally relevant exposures during late embryonic development. At this stage, many regulatory pathways are highly constrained, and small shifts in the expression of pathway components may reflect subtle modulation rather than overt disruption. The enrichment of multiple related pathways and the organization of differentially expressed genes within coherent protein–protein interaction networks suggest that these transcriptional changes are structured and non-random. Importantly, pathway- and network-based analyses integrate distributed expression signals across functionally related genes, providing a framework for interpreting coordinated transcriptional responses that may not be apparent from individual fold changes alone. While the present data do not establish functional or phenotypic consequences, they identify molecular pathways that may be sensitive to low-level exposure during a critical developmental window and warrant further investigation in future studies. It is important to note that the present findings are based on transcriptomic and network-level analyses and should be interpreted as hypothesis-generating rather than as evidence of direct functional or physiological endocrine disruption.

Within this system-level, computational analysis, a limited number of endocrine-associated network modules emerge as priority molecular targets based on their consistent involvement across differential expression, pathway enrichment, and GO-filtered protein–protein interaction subnetworks. Specifically, the highest-priority subnetworks include: (i) the estrogen metabolic/biosynthetic process and androgen metabolic/biosynthetic process subnetworks, which converge on a steroidogenic enzyme module centered on *CYP19A1* together with *HSD3B2*, *CYP17A1*, *SRD5A2*, and members of the *HSD17B* family; (ii) the androgen receptor signaling, regulation of androgen receptor signaling, and nuclear androgen receptor binding subnetworks, which highlight a nuclear receptor regulatory/proteostasis module involving *DNAJA1*, *HDAC6*, *PARK7* and RNA/chromatin co-regulators such as *DDX5/DDX17* and *EP300*; and (iii) pathway-level enrichment of MAPK signaling and regulation of the actin cytoskeleton, which provides a recurrent signaling backbone linking stress-responsive pathways with endocrine-associated network remodeling. Future studies should prioritize targeted validation of key transcriptomic nodes using RT-qPCR, paired with LC–MS/MS quantification of steroid hormones (e.g., testosterone, estradiol and precursors) and DEHP/MEHP in exposure media and larvae, as well as pathway-level reporter assays such as estrogen-responsive transgenic lines to directly assess transcriptional activity. Causal relationships could be further explored using perturb-and-rescue approaches (e.g., CRISPR/Cas9, transient knockdown of steroidogenic or chaperone-related genes, and hormone supplementation), together with phenotypic anchoring endpoints appropriate for the 96–120 hpf window, including locomotor behavior, early metabolic indicators, and developmental morphology or organ function.

Across estrogen-related GO-filtered PPI subnetworks, the dominant signal was likely a bottleneck in estrogen production, driven by consistent *CYP19A1* (aromatase) downregulation (−0.88), alongside modest remodeling of 17β-HSD interconversion (*HSD17B7* and *HSD17B10* mildly increased; *HSD17B8* decreased). These steroidogenic shifts were tightly coupled to mitochondrial/peroxisomal metabolism and redox handling, including mixed CYP responses, and a pronounced drop in the electron donor *CYB5A*, suggesting constrained oxidative metabolism capacity. In parallel, Phase II clearance pathways were rebalanced rather than uniformly induced, with increased *UGT1A1/UGT1A8* but decreased *UGT1A7/UGT1A9/UGT2B15*, consistent with isoform switching that could redirect the fate of estrogen derivatives. Estrogen-response subnetworks further indicated that steroid signaling is being routed through broader stress and co-regulator systems: an NR3C1-centered chaperone/stress axis (with *FKBP5* and *PER2* reduced) and an SRC-linked module enriched for integrins and MAPK components, consistent with prominent non-genomic crosstalk and adhesion/migration signaling even without major SRC transcriptional change.

Androgen-related subnetworks similarly supported attenuated upstream androgen-associated transcriptional signatures, with strong downregulation of *HSD3B2* and *CYP17A1*, plus reduced *SRD5A2*, implying diminished precursor formation and downstream activation. A potential compensatory feature was the robust increase in *HSD17B3*, suggesting maintenance of terminal testosterone-forming capacity despite limited substrate supply. Notably, androgen metabolism/biosynthesis networks were interwoven with developmental morphogen programs, including strong induction of *WNT6* and increased *WNT4* and a broad SHH-linked patterning cluster, indicating that steroidogenic pathway modulation occurs within a rewired developmental signaling context. Consistent with this, AR-associated subnetworks emphasized regulatory machinery rather than AR itself, with repeated hubs in RNA processing and chromatin control (*DDX5/DDX17* and *EP300/SETD1A*) and prominent proteostasis adaptations (*DNAJA1/HDAC6/PARK7* with *BAG2* reduced), pointing to a shift in how androgen-responsive transcription is executed under exposure-related stress. Because zebrafish embryos are not sexually differentiated at the 96–120 hpf stage, the androgen-associated patterns identified here should not be interpreted as sex-specific or reproductive outcomes. Rather, the coordinated modulation of steroidogenic enzymes and androgen-related regulatory machinery observed in this study reflects transcriptional signatures associated with developing endocrine and metabolic frameworks. These findings are therefore best interpreted as alterations in early-life endocrine-associated transcriptional programming, rather than evidence of functional androgen disruption or reproductive impairment.

A key conceptual advance of the present work is the integration of estrogen- and androgen-related processes into a unified sex-steroid signaling network, rather than treating these axes as independent or hierarchically ordered pathways. While phthalates such as DEHP and its active metabolite MEHP are classically characterized as anti-androgenic compounds, the network analysis highlights coordinated perturbation of steroid biosynthesis, metabolic processing, receptor binding, and receptor-mediated transcription across both estrogenic and androgenic pathways upon exposure to environmental levels of DEHP. This system-level framing moves beyond single-receptor or single-hormone models and better reflects the transcriptomic responses observed in whole-embryo zebrafish, where multiple endocrine pathways are concurrently active and developmentally interdependent.

Notably, the network distinguishes between hormone receptor signaling and regulation of receptor function, including nodes related to nuclear receptor binding and modulation of androgen receptor signaling. This distinction is rarely emphasized in zebrafish embryo toxicogenomic studies but is particularly relevant for phthalates, which often act through indirect mechanisms such as altered steroid availability, co-regulator activity, or metabolic stress rather than strong receptor agonism or antagonism. By capturing these regulatory layers, the network provides a mechanistic framework that is consistent with transcriptomic enrichment of estrogen- and androgen-responsive genes in the absence of overt gonadal or reproductive phenotypes during early development. Importantly, the absence of gonad- or reproduction-specific processes within the network underscores its relevance to early-life stages, where endocrine transcriptional modulation manifests as altered developmental programming rather than reproductive impairment.

A limitation of the present study is that DEHP concentrations in the exposure media were defined on a nominal basis and were not analytically verified over the 24 h exposure period. Biologically, the observed response likely reflects a combination of parent DEHP exposure and early metabolic conversion to MEHP within larvae. The short exposure duration (24 h) and absence of media renewal were intentional design choices aimed at limiting experimental manipulation during a critical developmental transition (96–120 hpf). However, this design does not account for potential time-dependent losses due to sorption or uptake. Future studies incorporating chemical verification (e.g., LC–MS/MS quantification of DEHP and/or MEHP in exposure media) and/or the use of low-sorption materials (e.g., glass vessels or pre-conditioned plastics) will be important to more precisely define internal and external exposure dynamics. Despite this limitation, the nominal concentration used (10^−8^ M) lies within the range reported for DEHP and its metabolites in environmental matrices and human biomonitoring studies, supporting the relevance of the exposure scenario for hypothesis-generating toxicogenomic analyses. Additionally, the transcriptomic responses observed likely reflect a combination of parent compound exposure and early metabolic conversion to MEHP within larvae, consistent with known phthalate biotransformation pathways. As the transcriptomic responses were assessed at a single developmental time point, this study cannot determine whether the observed changes are transient or persistent, and longitudinal analyses will be required to evaluate their potential role in developmental programming.

The present study is limited to transcriptomic and network-based analyses and does not include phenotypic, hormonal, or biochemical measurements. Consequently, the results should be interpreted as descriptive of molecular-level associations with exposure rather than as evidence of functional endocrine effects. The observed changes may reflect subtle regulatory modulation during a sensitive developmental period, but their physiological relevance cannot be determined from the current data. Additional studies incorporating functional endpoints, such as hormone measurements or developmental and behavioral assessments, will be necessary to evaluate the biological significance of these transcriptomic patterns. Protein–protein interaction analyses in this study were constructed using the human STRING interactome, which may not fully capture species-specific interactions or the precise developmental context of zebrafish embryos. This approach was adopted because core components of endocrine signaling and nuclear receptor pathways are highly conserved across vertebrates, allowing human interaction data to serve as a reasonable reference framework for exploratory network analysis.

This study focuses on a restricted developmental window (96–120 hpf), which may limit generalization to earlier morphogenetic stages or later life periods. This interval was selected intentionally to capture a phase of functional maturation and physiological integration, rather than early organ patterning, when subtle regulatory perturbations may become transcriptionally apparent. While the present findings are therefore specific to this developmental stage, they provide insight into molecular responses during a period of heightened functional sensitivity. Future studies examining additional developmental windows will be needed to determine the temporal specificity and persistence of these transcriptomic patterns. A further limitation of this study is the use of a single DEHP concentration, which precludes assessment of dose–response relationships. This exposure level was selected to represent environmentally relevant, low-dose conditions and to avoid redundancy with prior studies that have already characterized transcriptomic and toxicological effects at higher concentrations. Accordingly, the study was designed to complement the existing high-dose literature by focusing on molecular responses at a realistic exposure level rather than to define dose-dependent thresholds. Future studies incorporating multiple concentrations will be necessary to determine how these transcriptomic patterns relate to established high-dose effects and to evaluate potential non-linear responses across exposure ranges.

In conclusion, this study aligns with emerging evidence from zebrafish embryo and larval RNA-seq studies showing that DEHP and MEHP disrupt neurodevelopmental, metabolic, and cardiac pathways through hormone-sensitive signaling cascades. As such, the proposed estrogen–androgen network may serve as a useful conceptual bridge between molecular initiating events involving phthalates and downstream developmental outcomes, supporting its application in adverse outcome pathway (AOP) development and comparative toxicogenomics.

## Data Availability

RNA-seq data have been submitted to the NCBI Gene Expression Omnibus, accession number GSE100367.

## Supplementary Materials

The following supporting information can be downloaded at https://www.mdpi.com/article/10.3390/genes17030257/s1, Supplementary File S1: Gene-Level Results; Supplementary File S2: Estrogen- and Androgen-Related Pathway Network; Table S1: KEGG_ORA_results; Table S2: KEGG_GSEA_results.
