# Supplementary material for: Transcriptomic Responses of Zebrafish Embryos to Environmentally Relevant, Low-Dose (2-Ethylhexyl) Phthalate Exposure at 96–120 hpf
Source: Genes (Basel). 2026 Feb 25;17(3):257. doi: 10.3390/genes17030257 (PMC13025252; doi:10.3390/genes17030257)
Supplement: Supplementary file 1 [file genes-17-00257-s001.zip › Supplemental File S1_Gene Level_Results_revision.pdf]

Transcriptomic Responses of Zebrafish Embryos to Environmentally Relevant, Low-Dose (2-Ethylhexyl) Phthalate Exposure at 96–120 hpf

Mariagiovanna Pais[1], Kate McCafferty[1], Guillermo Lopez-Campos[2], Gary Hardiman[1*,3]

[1]Faculty of Medicine, Health and Life Sciences, School of Biological Sciences, and Institute for Global Food Security; [2]Wellcome-Wolfson Institute for Experimental Medicine, Queen's University Belfast, Belfast, Northern Ireland, UK; [3]Department of Medicine, University of California San Diego, La Jolla, CA USA.

Mariagiovanna Pais

ORCID: 0009-0007-5027-9950

Guillermo Lopez-Campos

ORCID: 0000-0003-3011-0940

Gary Hardiman

ORCID: 0000-0003-4558-0400

[*] Gary Hardiman g.hardiman@qub.ac.uk

Supplemental Results

Estrogen Metabolic Process Subnetwork

The GO-filtered PPI subnetwork associated with the estrogen metabolic process revealed a coordinated modulation of enzymes involved in estrogen biosynthesis, interconversion and detoxification. Proteins annotated to the GO term estrogen metabolic process, depicted in Figure 3A and highlighted in purple, include *CYP19A1, CYP11B2, HSD17B7, HSD17B8, HSD17B10, CYP1A1, CYP1B1, CYP3A5, CYB5A, UGT1A1, UGT1A7 and UGT2B15.* Additional interacting nodes in the subnetwork are annotated to related biological processes, including small molecule metabolism, redox regulation, conjugation, transcriptional regulation, and signaling pathways, as defined by Gene Ontology. *CYP19A1* (aromatase), the key enzyme converting androgens to estrogens, was downregulated ($log_2FC = -0.88$), suggesting a potential reduction in local estradiol synthesis during early embryogenesis. In the same module, *CYP11B2* ($log_2FC = +0.23$), a mitochondrial aldosterone synthase that influences overall steroid production, was associated with *CYP19A1*. Within the 17β-hydroxysteroid dehydrogenase module, *HSD17B7* ($log_2FC = +0.12$) and *HSD17B10* ($log_2FC = +0.29$) were modestly upregulated, whereas *HSD17B8* ($log_2FC = -0.23$) was reduced, consistent with altered interconversion between active and inactive estrogens and androgens. This enzymatic block was connected in the PPI network to additional metabolic partners such as *ACAA1* ($log_2FC = +0.05$), reflecting lipid-derived substrate processing, and to transcriptional or transport-associated interactors including *RXRB* ($log_2FC = +0.05$), *KIFC1* ($log_2FC = -0.17$), and the glucuronosyltransferase *UGT1A9* ($log_2FC = -0.61$), indicating broader coupling between steroid metabolism, nuclear receptor signalling, and conjugation pathways. Enzymes involved in estrogen metabolism and associated redox processes showed mixed responses: *CYP1A1* ($log_2FC = +0.12$), *CYP1B1* ($log_2FC = -0.10$), and *CYP3A5* ($log_2FC = -0.08$), together with the electron donor *CYB5A* ($log_2FC = -1.01$). Within this same interaction cluster, the network included *MGST2* ($log_2FC = -0.13$), a glutathione-dependent oxidoreductase, and *ASMT* ($log_2FC = +0.54$), a melatonin-synthesis enzyme intersecting indole and estrogen-related metabolism. *HSD17B10* was further linked to *RPP40* ($log_2FC = -0.03$), a ribonuclease P/MRP subunit that appeared as a connected node in this metabolic–redox region.

Additional retinoid-related oxidoreductases annotated to this GO term, including *RETSAT* and *DHRS9*, reinforce connections between retinoid and steroid/redox metabolism. Conjugation-related enzymes involved in estrogen metabolism also showed clear reorganization. *UGT1A1* ($log_2FC = +0.64$) and *UGT1A8* ($log_2FC = +0.57$) were increased, while *UGT1A7* ($log_2FC = -2.60$), *UGT1A9* ($log_2FC = -0.61$), and *UGT2B15* ($log_2FC = -0.13$) were downregulated. Within this UGT-centred cluster, the PPI network also connected *P2RY12* ($log_2FC = -0.83$) to *CYP3A5*, reflecting STRING-derived proximity between metabolic and signaling nodes rather than direct biochemical coupling.

Additional peripheral nodes interacting with metabolic enzymes included *PDGFRA* ($log_2FC = +0.11$), *VEGFC* ($log_2FC = -1.34$), *VCAM1* ($log_2FC = -0.08$), *DNM2* ($log_2FC = -0.48$), and *SOX2* ($log_2FC = +0.10$). Although not directly involved in estrogen metabolism, their presence reflects regulatory alliances linking growth

factor signaling, vascular pathways, adhesion, and cytoskeletal remodeling to metabolic states. Finally, *COMT* (log_2FC = +0.15), responsible for O-methylation of catechol estrogens, was linked to *UGT2B15*, consistent with the involvement of conjugative enzymes implicated in estrogen metabolism and clearance (Supplementary File 1. Networks of Estrogen- and Androgen-Associated Pathways).

Estrogen Biosynthetic Process Subnetwork

The GO-filtered PPI subnetwork associated with the estrogen biosynthetic process revealed a network dominated by core biosynthetic enzymes and their functionally connected interactors (Figure 3B). Proteins annotated to the GO term estrogen biosynthetic process, highlighted in yellow in Figure 3B, include *CYP19A1, CYP11B2, HSD17B7, HSD17B8* and *DHRS11.* Several of these proteins, including *CYP19A1, CYP11B2* and *HSD17B8*, are also annotated to the estrogen metabolic process. Within this network, interactions were largely centered on CYP19A1 (aromatase), which showed strong downregulation (log_2FC = −0.88). Given the central role of aromatase as the rate-limiting enzyme in estrogen synthesis, this reduction likely reflects a diminished estrogen-production capacity. Additional steroidogenic enzymes interacting with *CYP19A1* included *CYP11B2* (log_2FC = +0.23), a mitochondrial enzyme primarily involved in corticosteroid and mineralocorticoid synthesis, and *CYP27B1*, another mitochondrial hydroxylase connected within this cluster. Two further interactors, *POMC* (log_2FC = −0.25), the precursor of *ACTH* that modulates adrenal steroidogenic output and *EPHX1* (log_2FC = −0.77), implicated in estrogen-related epoxide metabolism, also mapped onto the CYP19A1 module. Members of the hydroxysteroid dehydrogenase family displayed mild transcriptional changes. *HSD17B*7 (log_2FC = +0.12), responsible for estrone-to-estradiol conversion and *HSDL2* (log_2FC = +0.03), a mitochondrial/peroxisomal oxidoreductase showed slight upregulation. In contrast, *HSD17B8* (log_2FC = −0.23), which inactivates estrogens and androgens and *DHRS11* (log_2FC = −0.25), a 17β-HSD-like reductase were downregulated. Within the same module, *HSD17B8* served as the local interaction hub, connecting to *RXRB* (log_2FC = +0.05), a retinoid X receptor subunit influencing steroid-responsive gene expression; *KIFC1* (log_2FC = −0.17), a kinesin-14 motor linked to organelle organization; *UGT1A9* (log_2FC = −0.61), a glucuronosyltransferase metabolizing estrogen derivatives; *UGT1A8* (log_2FC = +0.57), involved in extrahepatic estrogen conjugation; *ECHS1* (log_2FC = −0.34), a β-oxidation enzyme; *ACAA1* (log_2FC = +0.05), a peroxisomal thiolase; and *AK2* (log_2FC = +0.22), a mitochondrial adenylate kinase supporting ATP turnover. These interactions reflect coupling between estrogen metabolism and cellular energy/lipid-processing pathways. Finally, a small peripheral module associated *DHRS11* (log_2FC = −0.25) with *GAPDH* (log_2FC = −0.15), a glycolytic enzyme frequently acting as a metabolic hub, suggesting an additional interface between redox/steroid metabolism and core energetic processes. (Supplementary File 1. Networks of Estrogen- and Androgen-Associated Pathways).

Estrogen Response Element Binding Subnetwork

The GO-filtered PPI subnetwork associated with estrogen response element binding includes *NR3C1, NR3C2, ESRRG and STRN*, highlighted in green in Figure 3C. In addition to these annotated nodes, the subnetwork retains interacting proteins annotated to other Gene Ontology biological processes, reflecting functional connections between estrogen response element binding and broader signalling, transcriptional, metabolic and cellular regulatory pathways. *NR3C1* showed mild upregulation ($log_2FC = +0.19$) and interacted with several canonical glucocorticoid-responsive partners, including *FKBP5* ($log_2FC = -0.50$), a co-chaperone that modulates receptor sensitivity, *HSP90AB1* ($log_2FC = -0.13$), a key chaperone required for steroid receptor activation, and *HSPA1L* ($log_2FC = +0.24$), part of the *HSP70* cycle involved in nuclear receptor quality control. Among downstream signaling mediators, *SGK1* ($log_2FC = +0.25$) appeared as a glucocorticoid-inducible kinase integrating metabolic and stress pathways. The ubiquitin ligase *TRIM63* ($log_2FC = -0.45$), another NR3C1 interactor, is consistent with glucocorticoid-dependent metabolic adaptation. Additional interacting transcription factors included *STAT3* ($log_2FC = +0.91$), a mediator of inflammatory–hormonal crosstalk, and *FOXO3* ($log_2FC = +0.22$), which integrates stress signaling within nuclear receptor networks. The subnetwork also contained regulators of circadian and neuroendocrine cycles, such as *PER2* ($log_2FC = -2.17$), previously linked to estrogen receptor–dependent transcriptional rhythms, and *CRH* ($log_2FC = +0.18$), a central hormone of the HPA axis feeding back on glucocorticoid signaling. Metabolic and redox regulators, including *INS* ($log_2FC = -1.20$) and *TXN* ($log_2FC = -0.18$), also interacted with *NR3C1*, reflecting their roles in modulating steroid receptor activity. A parallel node involved *NR3C2* ($log_2FC = +0.58$), the mineralocorticoid receptor, closely related to *NR3C1* and sharing overlapping pathways, together with reduced expression of its scaffolding modulator *STRN* ($log_2FC = -0.80$), consistent with coordinated modulation of corticosteroid-responsive signaling. Finally, an additional small interaction pair involved the nuclear receptor *ESRRG* (−0.07) and *HIF1A* (−0.15), forming a peripheral component within the estrogen response element binding subnetwork and linking metabolic and hypoxic regulation. (Supplementary File 1. Networks of Estrogen- and Androgen-Associated Pathways).

Estrogen Receptor Signaling Pathway Subnetwork

The GO-filtered PPI subnetwork associated with the estrogen receptor signaling pathway comprises a core set of proteins directly annotated to this GO term, including *SRC, DDX17, SKP2, STRN3, CNOT1 and CYP7B1,* shown in red (Figure 4A). In addition to these ER-signaling components, the subnetwork incorporates multiple interacting proteins annotated to other Gene Ontology biological processes, such as kinase-mediated signal transduction, cell adhesion and migration, RNA processing and chromatin-associated regulation, protein turnover and metabolic pathways, reflecting the integration of estrogen receptor signaling within broader cellular regulatory networks.

This subnetwork revealed a configuration centered around *SRC,* reflecting the well-established role of SRC-dependent kinase activity as a core regulatory module that supports both genomic and non-genomic estrogen receptor (ER) signaling. Although *SRC* itself showed minimal transcriptional change ($log_2FC = +0.01$), it

emerged as the dominant interaction hub because ER activation relies on extensive kinase, adhesion, and co-regulator networks that STRING aggregates within this GO category. A prominent group of *SRC* interactors included several integrins, *ITGA11* (log_2FC = +1.37), *ITGA5* (log_2FC = +0.12), and *ITGA10* (log_2FC = –0.09), together with adhesion-related partners such as *EFNB1* (log_2FC = +0.77) and *L1CAM* (log_2FC = +0.23). These molecules define an adhesion- and migration-associated signaling environment characteristic of SRC-driven non-genomic ER activation. Additional adhesion and migration regulators interacting with *SRC* included *DLG1* (log_2FC = –0.12) and *LRP5* (log_2FC = +0.02), strengthening the involvement of cytoskeletal and membrane-anchored signaling scaffolds in this cluster. *GAS6* (log_2FC = –0.15), a ligand activating AXL/TAM receptors upstream of *SRC*, also appeared within the same module, suggesting additional feed-forward cues promoting kinase activation. Multiple components of MAPK-linked cascades were also present, including *MAPK12* (log_2FC = +1.88), *MAP2K2* (log_2FC = –0.29), and *CHUK* (log_2FC = +0.13). Their coordinated modulation indicates engagement of kinase pathways known to amplify ER signaling through phosphorylation-dependent mechanisms. Elements of G-protein signaling, such as *GNAQ* (log_2FC = +0.13) and *GNA11* (log_2FC = –0.64), further reinforce the involvement of SRC-associated ER crosstalk circuits. Within the same signaling module, *GRAP2* (log_2FC = +0.43), a cytosolic adaptor integrating receptor-derived cues with MAPK pathways, was also detected, indicating an additional layer of SRC-linked signal coordination. Within this kinase-associated group, the presence of *STK25* (log_2FC = –0.58) and *STRN3* (log_2FC = +0.03), components of the STRIPAK regulatory axis, suggests parallel modulation of polarity and cytoskeletal organisation intersecting with SRC activity.

A second cluster was built around *DDX17*, a recognized nuclear ER co-regulator, which interacted with several RNA-binding and chromatin-associated proteins, including *HNRNPA1* (log_2FC = +0.24), *HNRNPK* (log_2FC = +0.12), *SF1* (log_2FC = +0.66), and *YBX1* (log_2FC = –0.25). This group represents post-transcriptional and chromatin-remodeling processes required for fine-tuning ERα DNA binding and transcriptional output. *EIF4A1* (log_2FC = –0.06), a translation-initiation helicase associated with ER-dependent transcriptional programs, also interacted with *CNOT1*, further anchoring this post-transcriptional regulatory cluster. Regulators of protein turnover also appeared within the network. *SKP1* (log_2FC = +0.19) and *SKP2* (log_2FC = +0.87) suggested the engagement of ubiquitin–proteasome pathways that control ER cofactor cycling. Additional elements such as *CNOT1* (log_2FC = +0.20), *GUK1* (log_2FC = –0.59), and *MYL9* (log_2FC = +0.38) pointed to auxiliary contributions from mRNA-decapping machinery, metabolic regulation, and cytoskeletal organisation. The remaining metabolic enzyme, *CYP2W1* (log_2FC NA), and steroid-metabolizing *CYP7B1* (log_2FC = +0.13) formed a small peripheral branch associated with alternative ligand-processing routes that may indirectly interface with SRC-linked ER signaling. (Supplementary File 1. Networks of Estrogen- and Androgen-Associated Pathways).

Cellular Response to Estrogen Stimulus Subnetwork

Proteins annotated to the GO term cellular response to estrogen stimulus, depicted in green in Figure 4B, include *MDM2, RARA, RBX1, RYBP, RPS27A, PELP1, WBP2, UQCRC1, KANSL1, CRHBP, SFRP1* and *SFR1*. In addition to the estrogen-responsive core, the network includes proteins annotated to processes involving ribosomal function, ubiquitin–proteasome activity, chromatin and transcriptional regulation, DNA repair, mitochondrial metabolism, cell-cycle regulation, and Wnt-related signaling. The subnetworks revealed a bipartite architecture dominated by two major regulatory hubs, MDM2 and RARA, each anchoring a distinct signaling module relevant to estrogen-linked transcriptional responses. *MDM2*, which was upregulated (log_2FC = +0.67), formed the densest interaction cluster in the network. Its central role as an E3 ligase regulating p53 turnover is well established. Within this module, *MDM2* was connected to several ribosomal components, including *RPS26* (log_2FC = +0.31), *RPL37* (log_2FC = +0.12) and *RPS27A* (log_2FC = −0.01), which together reflect changes in 40S/60S subunit metabolism and ubiquitin–ribosome coupling. The same cluster comprised *UBE2S* (log_2FC = +0.59) and *RBX1* (log_2FC = +0.22), key ubiquitin–proteasome regulators controlling K11-linked chain elongation and cullin–RING ligase activity, and two p53-associated factors, *RRM2B* (log_2FC = −0.65), a p53-inducible ribonucleotide reductase subunit involved in DNA repair, and *CDKN2AIP* (log_2FC = −0.01), a modulator of p53 stability and checkpoint control. Together, these interactions support a scenario in which *MDM2* not only regulates p53 degradation but also interfaces with ribosome biogenesis, ubiquitin signaling and DNA-damage–responsive effectors. Additional interactors in the *MDM2* hub included *MYL6B* (log_2FC = −0.14), a myosin light-chain isoform associated with cytoskeletal organisation, and *TCHP* (log_2FC = +0.13), a centrosome- and proteostasis-associated factor, suggesting links between estrogen-responsive stress signaling, cytoskeletal remodeling and protein quality control. Chromatin-associated factors enriched around *MDM2* included *TAF1* (log_2FC = +0.49), the catalytically active subunit of *TFIID*, *UBTF* (log_2FC = −0.18), a nucleolar activator of RNA polymerase I transcription, and *RYBP* (log_2FC = +0.27), a Polycomb-associated transcriptional repressor. The connections to the early-response transcription factor *EGR1* (log_2FC = +0.80), which integrates rapid hormonal signals, and to the progesterone receptor *PGR*, further situate this MDM2-centred module within a broader steroid-responsive transcriptional cycle.

The second hub, *RARA*, showed minimal transcriptional modulation (log_2FC = −0.06) but maintained extensive connectivity. As a canonical retinoic-acid receptor, *RARA* interacted with its heterodimer partner *RARG*, which was modestly upregulated (log_2FC = +0.23) and is essential for retinoid-dependent chromatin remodeling (Kashyap et al., 2013), and with *CRABP2* (log_2FC = +0.63), which facilitates ligand delivery to nuclear receptors. Additional partners included *CEBPA* (log_2FC = +0.67), a transcription factor linking hormonal cues to metabolic programs (Lourenço and Coffer, 2017), *XPR1* (log_2FC = +0.04), a phosphate exporter, *MECR* (log_2FC = −0.13), the terminal enzyme of mitochondrial fatty-acid synthesis, and the replication-independent histone variant *H2BC21*, reflecting a convergence between retinoid signaling, chromatin structure and cellular metabolism. A linked node centered on *PELP1*, an ER coactivator involved in genomic and non-genomic estrogen signaling, connected RARA signaling to transcriptional and chromatin regulators such as *KANSL1* (log_2FC = +0.40), a component of the NSL

acetyltransferase complex (Dias et al., 2014), *RPL35A* (log_2FC = +0.08), a 60S ribosomal protein, and *DNTT*, a polymerase involved in V(D)J recombination. Within the same module, *CDK4* (log_2FC = +1.10), a G1/S cell-cycle kinase frequently engaged by hormone-dependent mitogenic signals, interacted with *PELP1*, further linking estrogen coactivator function to cell-cycle control. Additional interactors adjoining this module included *WBP2* (log_2FC = +0.32), a coactivator of estrogen and progesterone receptors, and two mitochondrial components, *UQCRC1* (log_2FC = −0.02) and *COA7* (log_2FC = +0.04), which contribute to respiratory-chain function and mitochondrial organisation. A smaller downregulated module comprised *SFRP1* (log_2FC = −1.06), an extracellular Wnt antagonist, *FZD5* (log_2FC = −1.94), a Frizzled-family Wnt receptor, and *VANGL2* (log_2FC = −0.02), a planar-cell-polarity component. These Wnt/PCP interactors are frequently implicated in developmental patterning and have established crosstalk with estrogen-responsive pathways. Finally, a distinct pair, *SFR1* (log_2FC = −2.44) and *XRCC3* (log_2FC = −0.04), both involved in homologous recombination repair, suggested that genome-stability mechanisms are embedded within the estrogen-related response.

Androgen Metabolic Process Subnetwork

The GO-filtered PPI subnetwork associated with androgen metabolic process comprises a set of enzymes and regulatory factors annotated to this GO term. These proteins, highlighted in orange in Figure 5A, include *HSD3B2, HSD17B3, HSD17B10, HSD17B8, CYP17A1, CYP19A1, CYP11B2, DHRS9, INHBA, SHH, WNT4 and MED1.* The remaining nodes in the subnetwork are annotated to distinct Gene Ontology categories and are retained as high-confidence interactors within the same interaction neighbourhood, indicating integration of androgen metabolism with developmental, transcriptional, mitochondrial and signalling processes. Central to the network was the coordinated modulation of short-chain dehydrogenases and cytochrome P450 enzymes that define androgen biosynthetic flux. *HSD17B3* (log_2FC = +1.00), which catalyzes the final reduction step from androstenedione to testosterone, interacted with the upstream enzyme *HSD3B2* (log_2FC = –0.83), indicating a shift in precursor supply paired with downstream reductive capacity. Additional enzymes in the pathway, including *HSD17B10* (log_2FC = +0.29) and *HSD17B8* (log_2FC = –0.23), contributed to a broader reorganization of dehydrogenase activity linked to mitochondrial and cytosolic steroid metabolism. Consistent with its interaction pattern in the network, *HSD17B8* was additionally connected to *RXRB* (log_2FC = +0.05), a retinoid X receptor subunit influencing nuclear receptor signaling to *KIFC1* (log_2FC = –0.17), a kinesin-family motor protein associated with intracellular transport, and to *UGT1A9* (log_2FC = –0.61), a phase-II glucuronidation enzyme contributing to androgen metabolite clearance. *SULT2B1* (log_2FC = +0.49) also interacted with *HSD3B2* (log_2FC = –0.83), placing phase-II sulfonation within the same local steroidogenic module.

Two cytochrome P450 enzymes also emerged as key nodes. *CYP17A1* (log_2FC = –0.99), responsible for generating DHEA and androstenedione, interacted in the network with *DHH* (log_2FC = +0.77) and with *CYP19A1* (log_2FC = –0.88), situating these enzymes within a connected module influencing androgen–estrogen interconversion. The *CYP17A1* module also incorporated BMP15, a growth factor linked to ovarian follicle development. *RETSAT* (log_2FC = –0.40) and *DHRS9* (log_2FC

= –0.31), both retinol/steroid-related oxidoreductases, formed an additional low-expression pair adjacent to the core steroidogenic cluster. *CYP11B2* (log_2FC = +0.23) connected with *CYP19A1*, integrating mineralocorticoid-associated flux with aromatase-dependent pathways.

In parallel, the subnetwork incorporated a set of developmental regulators with established roles in gonadal and reproductive tract patterning. *WNT6* (log_2FC = +3.48) showed strong upregulation and interacted with *WNT4* (log_2FC = +0.64), forming a canonical Wnt–Wnt signaling pair. *WNT4* was also associated with *CCN5* (log_2FC = +0.53), a matricellular regulator linked to proliferative and morphogenetic processes. *SHH* (log_2FC = +0.16) contributed multiple interactions, including connections to *FGF16* (log_2FC = +1.38), *GDNF* (log_2FC = +0.61), *DLX5* (log_2FC = –0.36), *EMX2* (log_2FC = –0.63), *OLIG1* (log_2FC = +0.39), *EN2* (log_2FC = +0.27), *WNT3* (log_2FC = –1.61), *DISP3* (log_2FC = –1.72), and *CDH1*. This SHH-centered cluster placed morphogen signaling, neuronal patterning factors, and epithelial regulators in continuity with the transcriptional effects induced by altered androgen metabolism.

A regulatory cluster centered on *MED1* (log_2FC = –0.02), a multifunctional coactivator for androgen, estrogen, and PPAR nuclear receptors, connected several transcription- and chromatin-associated proteins, including *IFI35* (log_2FC = +0.14), *CHD9* (log_2FC = –0.07), *POLR2K* (log_2FC = –0.02), *SERBP1* (log_2FC = –0.20), *VEZT* (log_2FC = –0.82), *WDR31*, *H4C6* and MRPL47 (log_2FC = –0.12). Additional mitochondrial ribosomal components, *MRPL16* (log_2FC = +0.10) and *MRPL17* (log_2FC = +0.71) also interacted with *MED1*, linking steroid receptor coactivation with mitochondrial translational capacity. Finally, several peripheral interactions further extended the network architecture, including *UGDH* (log_2FC = +0.19) linked to *HSD3B2* (log_2FC = –0.83), *ACVR1* (log_2FC = +0.27) paired with *INHBA* (log_2FC = –0.28) and *RAC1* (log_2FC = –0.12) positioned adjacent to *WNT4* (log_2FC = +0.64).

Androgen Biosynthetic Process Subnetwork

The GO-filtered PPI subnetwork associated with the androgen biosynthetic process is organised around a set of core biosynthetic components, in light green in the Figure 5B, including *HSD3B2, CYP17A1, MED1, WNT4 and SRD5A2*. Additional interacting nodes are annotated to related Gene Ontology categories and capture developmental signalling, transcriptional co-regulation, mitochondrial function and metabolic processes that are functionally linked to androgen biosynthesis.

The subnetwork revealed a coordinated modulation of key steroidogenic enzymes together with nuclear co-regulators and developmental signals. The network was dominated by two central steroidogenic nodes, *HSD3B2* and *CYP17A1*, both markedly downregulated (*HSD3B2* log_2 FC = −0.83; *CYP17A1* log $_2$FC = −0.99), consistent with an overall attenuation of early androgen precursor formation. *HSD3B2*, which catalyzes the conversion of pregnenolone and 17-hydroxypregnenolone into progesterone and 17-hydroxyprogesterone and thus supplies precursors for downstream androgen and glucocorticoid synthesis, was connected to *HSD17B3* (log_2 FC = +1.00), the terminal reductase converting androstenedione to testosterone. Within the same cluster, *HSD3B2* interacted with *SULT2B1* (log_2FC = +0.49), a sulfotransferase that regulates hydroxysteroid availability, the orphan nuclear receptor *NR4A2* (log_2FC = +0.77), and metabolic

enzymes *UGDH* (log_2FC = +0.19), *HAO1* (log_2FC = −0.32), *RPS6* (log_2FC = −0.37) and *EIF2B3* (log_2FC = −0.37), which contribute to glucuronidation, redox balance and translational control. Additional interactors included *GMPPB* (log_2FC = −0.01), linking steroidogenesis to protein glycosylation and membrane integrity. In parallel, *HSD17B3* formed a subcluster with the co-activator and ferritinophagy mediator *NCOA4* (log_2FC = −0.24), underlining the integration of iron handling and mitochondrial function with testosterone biosynthesis. Collectively, this configuration suggests a bottleneck at the *HSD3B2* step, with compensatory upregulation of downstream testosterone synthesis via *HSD17B3* and parallel adjustments in sulfation, transcriptional regulation and intermediary metabolism.

CYP17A1, which performs the dual 17α-hydroxylase and 17,20-lyase reactions required for the production of DHEA and androstenedione, was embedded in a second cluster that included *DAZL* (log_2FC = −1.20), a germ-cell RNA-binding protein essential for gametogenesis, *CYP27A1* (log_2FC = +0.60), a mitochondrial P450 involved in cholesterol oxidation, *DHH* (+0.77), a Sertoli-cell–derived Hedgehog ligand crucial for fetal Leydig cell differentiation and *NUDT1* (log_2FC = +0.01), which protects against oxidative nucleotide damage. *BMP15* was also present in this module with unavailable expression data, contributing to the broader TGF-β/ovarian signaling context.

The concomitant downregulation of *CYP17A1* and *SRD5A2* (log_2FC = −0.36), the 5α-reductase type 2 responsible for conversion of testosterone to dihydrotestosterone, together with a modest decrease of its interactor *NFYB* (log_2FC = −0.08), a CCAAT-box–binding transcription factor, indicates that both androgen precursor formation and downstream androgen activation may be compromised. A broader regulatory component of the subnetwork was centered on *MED1* (log_2FC = −0.02), a core Mediator subunit and shared co-activator for androgen, estrogen and other nuclear receptors (Jin et al., 2012). *MED1* was linked to several mitochondrial ribosomal proteins, including *MRPL16* (+0.10), *MRPL17* (log_2FC = +0.71) and *MRPL47* (log_2FC = −0.12), as well as to the mitochondrial aminoacyl-tRNA synthetases *LARS2* (log_2FC = −0.95) and *TARS2* (log_2FC = +0.57), highlighting coordination between mitochondrial translation and steroidogenic capacity. It also connected to the cholesterol efflux transporter *ABCA1* (log_2FC = 0.003), chromatin and RNA-regulatory proteins *CHD9* (log_2FC = −0.07) and *SERBP1* (log_2FC = −0.20), the anti-apoptotic ubiquitin ligase *BIRC6* (log_2FC = +0.51), the GEF *DOCK10* (log_2FC = −0.05), the basal transcription subunit *POLR2K* (log_2FC = −0.02) and stress-responsive factors *ANKRD1* (log_2FC = −0.07) and *IFI35* (log_2FC = +0.14). Additional interactors such as the ion channel *KCND1* (log_2FC = −0.54) and the mitochondrial rRNA methyltransferase *MRM2* (log_2FC = −0.26) further support a reorganization of excitability and mitoribosome function; *H4C6* and *WDR31* were present with unavailable log_2 FC values. This pattern suggests a reconfiguration of the transcriptional and metabolic environment in which androgen-responsive transcription takes place, even in the absence of large changes in *MED1* expression itself.

A developmental signaling module was clearly represented by *WNT4* (log_2FC = +0.64) and *WNT6* (log_2FC = +3.48), which showed strong upregulation and were connected to *GDF10* (log_2FC = −0.53), *RAC1* (log_2FC = −0.12), *CCN5* (log_2FC = +0.53) and *SALL1* (log_2FC = +0.45). *WNT4* and *WNT6* are classically associated with gonadal differentiation and pro-ovarian or anti-androgenic developmental

programs. The inclusion of these genes within the same subnetwork as *HSD3B2* and *CYP17A1* indicates that DEHP exposure affects both the enzymatic backbone of androgen biosynthesis and the developmental context in which these hormones act.

Androgen Receptor Signalling Pathway Subnetwork

The GO-filtered PPI subnetwork associated with the androgen receptor signalling pathway comprises a group of GO-annotated proteins, highlighted in turquoise in Figure 6A, including *DAXX, DDX17, EP300, RNF6, DNAJA1, PARK7, FOXP1, HDAC6* and *DAB2*. In addition to this core group, the subnetwork retains multiple interacting proteins annotated to other Gene Ontology categories, including RNA processing and splicing, chromatin organisation, ubiquitin-mediated protein turnover, redox regulation, membrane trafficking and kinase-mediated signalling, reflecting the broader regulatory environment that supports androgen receptor activity. Among the most connected nodes, *DAXX* exhibited a slight upregulation (log2FC = +0.06) and formed high-confidence interactions with *MAP3K5* (log_2FC = +0.46) and *RASSF1* (log_2FC = +0.80), a kinase–tumor suppressor module known to influence nuclear receptor turnover and stress-dependent transcriptional responses. *DAXX* also interacted with *CBX1* (log_2FC = +0.21) and the acetyltransferase *KAT5*, which showed a robust upregulation (log_2FC = +0.80), consistent with the involvement of both proteins in chromatin configuration and histone acetylation dynamics.

A second major regulatory cluster was centered around *DDX17*, a recognized co-regulator of both AR and ER. Although *DDX17* was only modestly upregulated (log_2FC = +0.19), it connected to several RNA- and chromatin-associated factors, including *HNRNPA1* (log_2FC = +0.25), *HNRNPK* (log_2FC = +0.13), *SF1/NR5A1* (log_2FC = +0.66), *YBX1* (log_2FC = –0.26) and *SNRPC* (log_2FC = –0.33), all of which participate in spliceosomal function or RNA processing. The coordinated modulation of these RNA-binding proteins suggests a reorganization of spliceosome activity and post-transcriptional regulation within androgen-responsive pathways. EP300, which displayed a slight decrease (log_2FC = –0.04), remained a central point of interaction through its connections with *SETD1A* (log_2FC = +0.21) and *CREB3L3* (log_2FC = –0.64), as well as *AGO2*, one of the most strongly downregulated genes in the network (log_2FC = –2.44). *EP300* and *SETD1A* are key modulators of chromatin accessibility and transcriptional activation, whereas *CREB3L3* and *AGO2* regulate metabolic and post-transcriptional processes, respectively. Their combined dysregulation indicates that chromatin remodeling and RNA-mediated repression may both be altered in response to DEHP, with potential consequences for AR transcriptional output.

Protein-folding and stability factors formed another distinct module. *DNAJA1*, a co-chaperone required for AR maturation and nuclear transport, exhibited a pronounced upregulation (log_2FC = +0.65), while its partner *PARK7/DJ-1*, known to stabilize AR under oxidative stress, was also modestly upregulated (log_2FC = +0.14). In contrast, *BAG2*, an inhibitor of HSP70-dependent folding, showed the strongest downregulation among protein-folding factors (log_2FC = –1.20). This pattern points toward a shift that may enhance AR protein stability and folding efficiency under chemical stress. Additional interactions involving *PRDX3* (log_2FC = –0.18), *SYT1* (log_2FC = +0.02) with *DAB2* (log_2FC = +0.41), and *KCNH2* (log_2FC = +0.01) with

DNAJA1 further supported a reconfiguration of redox balance, membrane trafficking and stress-linked pathways. Forkhead transcription factors within the network showed contrasting regulation: FOXP2 was significantly upregulated (log_2FC = +0.63), while *FOXP1*, a known negative regulator of AR signaling, was consistently downregulated (log_2FC = –0.56). A mild reduction of *SMAD2* (log_2FC = –0.15), a key mediator of TGF-β signaling and crosstalk with AR, further reflected shifts in transcription factor availability. Other nodes, including *DLEC1* (log_2FC = –0.45) interacting with the E3 ligase *RNF6* (log_2FC = +0.02), *HDAC6* (log_2FC = +0.44) with *DYSF* (log_2FC = –1.26), and *LZTS1* (log_2FC = +0.22) with *RNF6*, highlighted additional layers of regulation involving ubiquitination, chromatin deacetylation and cytoskeletal organization.

Regulation of Androgen Receptor Signalling Pathway Subnetwork

The GO-filtered PPI subnetwork associated with the regulation of androgen receptor signalling pathway consists of GO-annotated proteins shown in green in Figure 6B, including *EP300, PARK7, RNF6, DAB2, FOXP1, DDX5, SFRP1, HDAC6 and CREB3L3.* The remaining nodes are annotated to related Gene Ontology categories and represent interacting factors involved in RNA processing, chromatin regulation, proteostasis, vesicle trafficking and stress-response pathways functionally linked to androgen receptor regulation.

Within this network, a coordinated reorganization of transcriptional co-regulators, chromatin-modifying enzymes, RNA-processing factors and chaperone-mediated proteostasis components was observed. The RNA helicase DDX5, a known nuclear coactivator of steroid receptors, emerged as a central regulatory node. Although modestly downregulated (log_2FC = –0.29), *DDX5* was highly connected to a cluster of RNA-processing factors, including *SRRM2* (log_2FC = –0.21), a key splice-site selection regulator; *SLU7* (log_2FC = +0.02), which ensures proper 3′ splice-site usage; *PRPF38A* (log_2FC = +0.20), required for tri-snRNP dynamics (Xie et al., 1998); *ELAVL1/HuR* (log_2FC = –0.12), an RNA stabilizer influencing hormone-responsive gene expression; *HSPA8* (log_2FC = +0.10), a constitutive HSP70 chaperone; *ACTB* (log_2FC = –0.40), involved in nuclear architecture and transcriptional dynamics; and *SMAD5* (log_2FC = +0.01), a TGF-β pathway effector. Collectively, these changes point to a reduced splicing efficiency and coactivator capacity supporting AR transcription. A second regulatory core was centered on EP300, maintained strong links with transcriptional regulators including the ER-stress transcription factor *CREB3L3* (log_2FC = –0.64); the H3K4 methyltransferase *SETD1A* (log_2FC = +0.21); the developmental regulator *HOXD4* (log_2FC = +0.39); *KLF8* (log_2FC = –0.26), which modulates cell-cycle and EMT-related transcription; and *AGO2* (log_2FC = –2.44), the catalytic component of the RNA-induced silencing complex. This configuration suggests reprogramming of acetylation-dependent enhancer activity and chromatin accessibility—mechanisms crucial for AR-driven transcription.

Transcription factors were also differentially modulated. *FOXP2* was upregulated (log_2FC = +0.63) while *FOXP1* was downregulated (log_2FC = –0.56), consistent with their antagonistic roles in nuclear receptor regulation. Additional regulators, including *SMAD2* (log_2FC = –0.15) and the SUMO E3-ligase *PIAS1*

(log_2FC = +0.67), further suggest altered nuclear signaling equilibria capable of modifying AR transcriptional output. A robust chaperone and proteostasis-oriented module was also detected. *HDAC6*, a cytoplasmic deacetylase that affects AR stability and HSP90 function, was strongly upregulated (log_2FC = +0.44). The HSP40 co-chaperone *DNAJA1* (log_2FC = +0.65), known to promote AR folding and import, and the oxidative-stress regulator *PARK7/DJ-1* (log_2FC = +0.14), which prevents protein damage, were similarly elevated. In contrast, *DYSF* (log_2FC = –1.26), a membrane-repair protein linked to proteostasis, was markedly reduced. The module also connected with redox regulators such as *PRDX3* (log_2FC = –0.18), *NCF1* (log_2FC = +0.02), and the neuroendocrine enzyme *TH* (log_2FC = –1.54), the rate-limiting step in catecholamine synthesis, suggesting convergence of metabolic and oxidative cues on AR modulation. The network additionally encompassed a markedly downregulated Wnt–PCP module, including *SFRP1* (log_2FC = –1.06), a secreted Wnt antagonist; *FZD5* (log_2FC = –1.94), a Wnt receptor; and *VANGL2* (log_2FC = –0.02), a core PCP protein. These alterations align with established reciprocal interactions between Wnt and androgen signaling pathways. Endocytic regulators *DAB2* (log_2FC = +0.41) and *FCHO2* (log_2FC = -0.001) further support changes in receptor trafficking.

Nuclear Androgen Receptor (AR) Binding Subnetwork

Proteins annotated to the GO term *nuclear androgen receptor binding*, in green in Figure 7, include *DDX5, PRKCB, EP300, PARK7, FOXP1, RNF6, ITPR1* and *DAXX*. Several of these nodes (e.g., *DDX5, EP300, PARK7, FOXP1, RNF6 and DAXX*) are also retained in the regulation of androgen receptor signalling pathway subnetwork, indicating overlap between AR nuclear binding and upstream regulatory control. The remaining nodes are annotated to other Gene Ontology categories, mainly transcriptional co-regulators, chromatin remodelers, RNA-processing factors and nuclear chaperones.

Two major hubs emerged: *DDX5* and *PRKCB*, each defining distinct mechanistic layers of AR regulation. *DDX5*, a well-established AR coactivator, was modestly downregulated (log_2FC = –0.29), yet retained extensive connectivity with nuclear RNA-processing partners. These included *SRRM2* (log_2FC = –0.21), a major splicing regulator (Blencowe, 2006), *SLU7* (log_2FC = +0.02), a key 3′ splice-site selection factor, *PRPF38A* (log_2FC = +0.20), essential for U4/U6–U5 tri-snRNP assembly, *ELAVL1/HuR* (log_2FC = –0.12), a stabilizer of hormone-responsive transcripts, and *ACTB* (log_2FC = –0.40), which contributes to nuclear architecture and transcription factor dynamics. The module also included *SMAD5* (log_2FC = +0.01), a *BMP/TGF-β* signaling mediator, and *PIAS1* (log_2FC = +0.67), a SUMO E3 ligase known to regulate nuclear receptor transcription.

An additional regulatory node was represented by *DAXX* (log_2FC = +0.06), a nuclear transcriptional corepressor. In the STRING-derived subnetwork, *DAXX* displayed high-confidence interactions with *MAP3K5* (+0.46), a stress-activated kinase modulating apoptotic and transcriptional signaling (Ichijo et al., 1997), and *RASSF1* (+0.80; score 0.948), a tumor-suppressor scaffold that regulates MAP3K5 activation dynamics. This *DAXX–MAP3K5–RASSF1* mini-cluster forms a stress-responsive nuclear module that may influence chromatin accessibility and indirectly modulate AR transcriptional availability under DEHP exposure.

Together, this coherent DDX5-centered submodule suggests attenuation of AR-associated splicing and mRNA-processing functions, pathways known to be essential for AR transcriptional output.

A second regulatory core was centered on *EP300*, a master histone acetyltransferase required for AR chromatin engagement. Although *EP300* showed minimal change (log_2FC = –0.04), it displayed high-confidence interactions with *CREB3L3* (log_2FC = –0.64), a metabolic stress-responsive transcription factor, *SETD1A* (log_2FC = +0.21), an H3K4 methyltransferase promoting transcriptional activation, *HOXD4* (log_2FC = +0.39), a chromatin-patterning homeobox gene, and *KLF8* (log_2FC = –0.26), a transcriptional regulator implicated in AR-relevant cellular transitions. These interactions define a chromatin-remodeling module in which reduced *CREB3L3* and *KLF8*, combined with stable *EP300* and increased *SETD1A*, indicate a reorganization of AR coactivator balance.

Chaperone and redox-support proteins also formed a cohesive cluster. *DNAJA1*, an HSP40 co-chaperone essential for AR folding and nuclear import, was strongly upregulated (log_2FC = +0.65), while *PARK7/DJ-1*, a redox-active chaperone that stabilizes AR under oxidative conditions, showed moderate induction (log_2FC = +0.14). *PRDX3* (log_2FC = –0.18), a mitochondrial peroxiredoxin involved in redox homeostasis, interacted with *PARK7*, linking mitochondrial stress regulation to AR proteostasis. The network showed a downregulated signaling module centered on PRKCB, a kinase directly involved in AR phosphorylation and nuclear localization, was substantially decreased (log_2FC = –0.63). Its interactions connected it to Ca^{2+}-signaling components *ITPR1* (log_2FC = +0.72) and *ITPR2* (log_2FC = –0.83), both key intracellular Ca^{2+}-release channels; to *RRAS* (log_2FC = –0.34), a regulator of Ras/MAPK and PI3K signaling; to *ADCY2* (log_2FC = +0.27), a generator of cAMP signaling; to *INSR* (log_2FC = +0.28), a central PI3K–AKT regulator; and to *PAX8* (log_2FC = –0.16), involved in endocrine transcriptional programs. The coordinated downregulation of *PRKCB*, *RRAS* and *ITPR2* suggests impaired upstream signaling cascades that normally support AR activation cycles. Finally, forkhead transcription factors appeared as interacting elements shaping nuclear receptor specificity: *FOXP1* (log_2FC = –0.56), an AR-modulating transcription factor, was consistently downregulated, whereas *FOXP2* (log_2FC = +0.63), implicated in transcriptional regulation, was robustly upregulated. Their reciprocal regulation is consistent with a shift in nuclear receptor transcriptional programs in response to DEHP.
