# Supplementary material for: Transcriptomic Responses of Zebrafish Embryos to Environmentally Relevant, Low-Dose (2-Ethylhexyl) Phthalate Exposure at 96–120 hpf
Source: Genes (Basel). 2026 Feb 25;17(3):257. doi: 10.3390/genes17030257 (PMC13025252; doi:10.3390/genes17030257)
Supplement: Supplementary file 1 [file genes-17-00257-s001.zip › Supplementary File S2_Network_Level_results_revision.pdf]

Estrogen and Androgen-Related Pathway Networks

Estrogen metabolic process

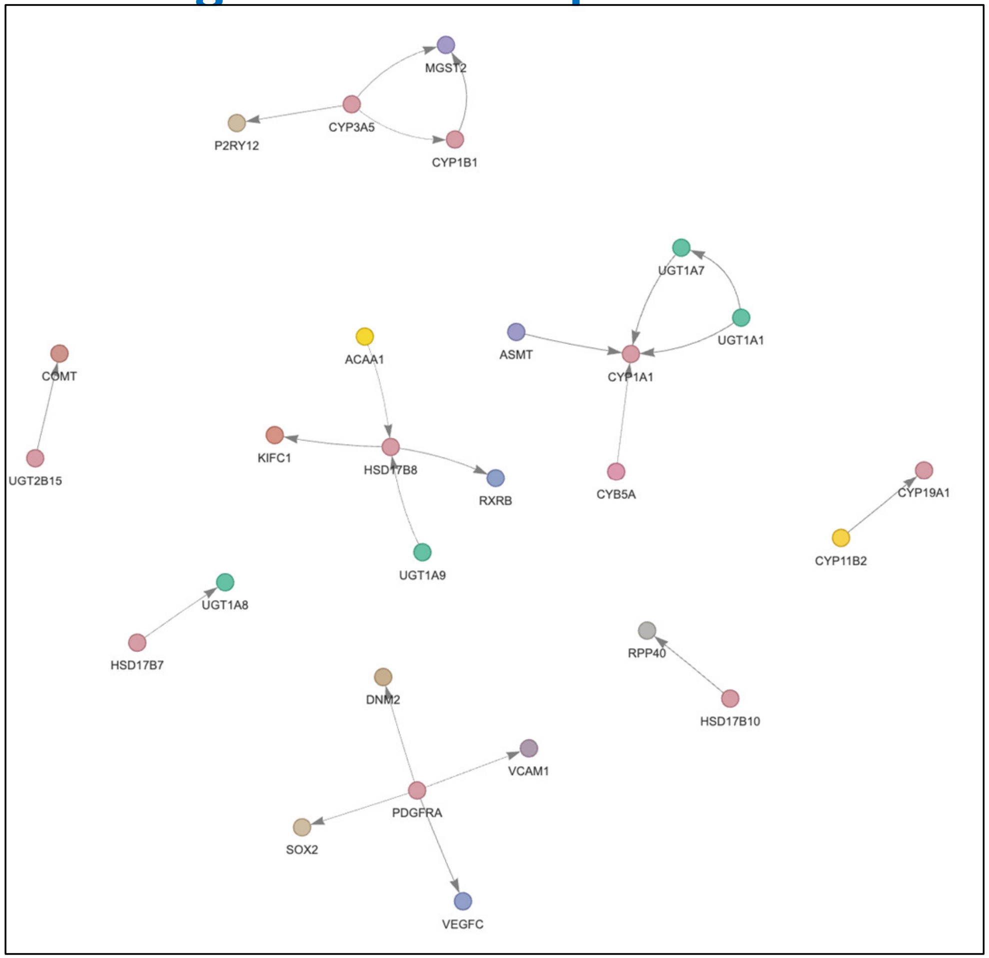

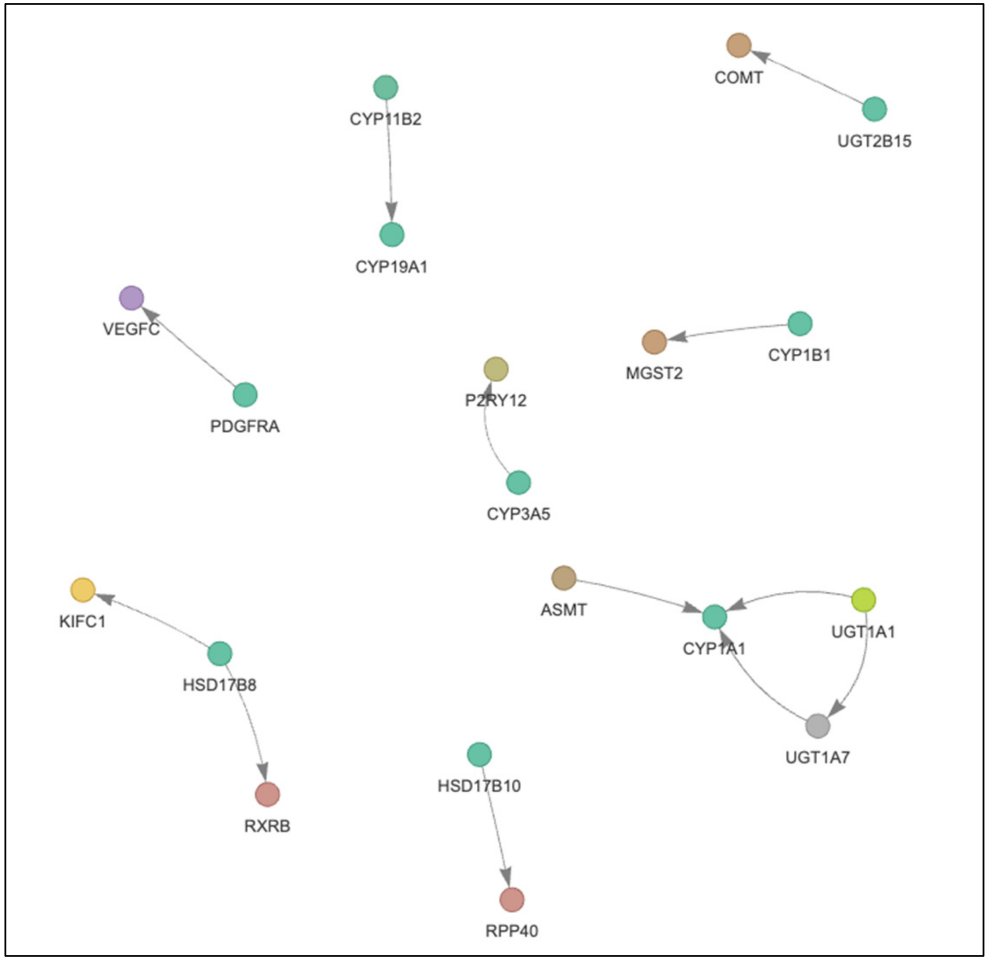

score = 0.4

score = 0.7

Estrogen metabolic process

stringId_A	stringId_B	preferredName_A	ensembl_gene_id_A	log2FoldChange_A	preferredName_B	ensembl_gene_id_B	log2FoldChange_B	score
**9606.ENSP00000304845**	9606.ENSP00000362525	UGT1A1	ENSG00000241635	0.638864207254496	UGT1A7	ENSG00000244122	-2.6030258915668	0.994
**9606.ENSP00000304845**	9606.ENSP00000378488	UGT1A1	ENSG00000241635	0.638864207254496	CYP1A1	ENSG00000140465	0.120453499068244	0.985
**9606.ENSP00000362525**	9606.ENSP00000378488	UGT1A7	ENSG00000244122	-2.6030258915668	CYP1A1	ENSG00000140465	0.120453499068244	0.985
**9606.ENSP00000325822**	9606.ENSP00000379683	CYP11B2	ENSG00000179142	0.228479383556454	CYP19A1	ENSG00000137869	-0.882362025384493	0.947
**9606.ENSP00000370639**	9606.ENSP00000378488	ASMT	ENSG00000196433	0.537937055004421	CYP1A1	ENSG00000140465	0.120453499068244	0.925
**9606.ENSP00000478561**	9606.ENSP00000482639	CYP1B1	ENSG00000138061	-0.104580358025444	MGST2	ENSG00000085871	-0.132064137265605	0.916
**9606.ENSP00000168216**	9606.ENSP00000369391	HSD17B10	ENSG00000072506	0.29147415617691	RPP40	ENSG00000124787	-0.0352293836860066	0.906
**9606.ENSP00000257290**	9606.ENSP00000480043	PDGFRA	ENSG00000134853	0.109697571185514	VEGFC	ENSG00000150630	-1.33976685050209	0.873
**9606.ENSP00000341045**	9606.ENSP00000354511	UGT2B15	ENSG00000196620	-0.12564926084382	COMT	ENSG00000093010	0.154391088748595	0.822
**9606.ENSP00000363794**	9606.ENSP00000393963	HSD17B8	ENSG00000204228	-0.233688080890375	KIFC1	ENSG00000237649	-0.170773448631387	0.82
**9606.ENSP00000363794**	9606.ENSP00000363817	HSD17B8	ENSG00000204228	-0.233688080890375	RXRB	ENSG00000204231	0.0479562150646334	0.798
**9606.ENSP00000222982**	9606.ENSP00000307259	CYP3A5	ENSG00000106258	-0.0791904537983588	P2RY12	ENSG00000169313	-0.833156486123551	0.726
**9606.ENSP00000222982**	9606.ENSP00000482639	CYP3A5	ENSG00000106258	-0.0791904537983588	MGST2	ENSG00000085871	-0.132064137265605	0.69
**9606.ENSP00000254521**	9606.ENSP00000362549	HSD17B7	ENSG00000132196	0.12137210049081	UGT1A8	ENSG00000242366	0.573370146693472	0.677
**9606.ENSP00000257290**	9606.ENSP00000373905	PDGFRA	ENSG00000134853	0.109697571185514	DNM2	ENSG00000079805	-0.480308082897979	0.668
**9606.ENSP00000346768**	9606.ENSP00000363794	UGT1A9	ENSG00000241119	-0.607976524541623	HSD17B8	ENSG00000204228	-0.233688080890375	0.666
**9606.ENSP00000257290**	9606.ENSP00000294728	PDGFRA	ENSG00000134853	0.109697571185514	VCAM1	ENSG00000162692	-0.0772286455679541	0.624
**9606.ENSP00000341625**	9606.ENSP00000378488	CYB5A	ENSG00000166347	-1.0113394828425	CYP1A1	ENSG00000140465	0.120453499068244	0.623
**9606.ENSP00000257290**	9606.ENSP00000323588	PDGFRA	ENSG00000134853	0.109697571185514	SOX2	ENSG00000181449	0.0972660213228138	0.602
**9606.ENSP00000333664**	9606.ENSP00000363794	ACAA1	ENSG00000060971	0.0533315410354744	HSD17B8	ENSG00000204228	-0.233688080890375	0.552
**9606.ENSP00000222982**	9606.ENSP00000478561	CYP3A5	ENSG00000106258	-0.0791904537983588	CYP1B1	ENSG00000138061	-0.104580358025444	0.541

score 0.4

**CYP1A1**	Phase I cytochrome P450 enzyme metabolizing estrogens and xenobiotics; produces catechol-estrogens.	Tydén E, Tjälve H, Larsson P. Gene and protein expression and cellular localisation of cytochrome P450 enzymes of the 1A, 2A, 2C, 2D and 2E subfamilies in equine intestine and liver. Acta Vet Scand. 2014;56(1):69. Published 2014 Oct 8. doi:10.1186/s13028-014-0069-8
**CYP1B1**	Phase I enzyme generating 4-hydroxy-estrogen metabolites; key role in estrogen redox cycling.	Hachey DL, Dawling S, Roodi N, Parl FF. Sequential action of phase I and II enzymes cytochrome p450 1B1 and glutathione S-transferase P1 in mammary estrogen metabolism. Cancer Res. 2003;63(23):8492-8499.
**CYP3A5**	Cytochrome P450 isoform involved in hepatic and extrahepatic metabolism of endogenous steroids including estrogens.	Zhang Y, Wang Z, Wang Y, et al. CYP3A4 and CYP3A5: the crucial roles in clinical drug metabolism and the significant implications of genetic polymorphisms. PeerJ. 2024;12:e18636. Published 2024 Dec 5. doi:10.7717/peerj.18636
**CYB5A**	Electron-donor hemoprotein that enhances activity of multiple CYP enzymes including steroidogenic CYPs.	SMa MY, Deng G, Zhu WZ, et al. Defects in CYB5A and CYB5B impact sterol-C4 oxidation in cholesterol biosynthesis and demonstrate regulatory roles of dimethyl sterols. Cell Rep. 2024;43(11):114912. doi:10.1016/j.celrep.2024.114912
**UGT1A1**	Phase II glucuronosyltransferase conjugating estrogens to inactive, water-soluble metabolites.	Skierka JM, O'Kane DJ. UDP-Glucuronosyltransferase 1A1 and the glucuronidation in oncology applications and hyperbilirubinemia. In: Molecular Diagnostics: Techniques and Applications for the Clinical Laboratory. Academic Press; 2010:409-419.
**UGT1A8**	Extrahepatic UGT isoform catalyzing glucuronidation of estrogens and xenobiotics.	Strassburg CP, Manns MP, Tukey RH. Expression of the UDP-glucuronosyltransferase 1A locus in human colon. Identification and characterization of the novel extrahepatic UGT1A8. J Biol Chem. 1998;273(15):8719-8726. doi:10.1074/jbc.273.15.8719
**UGT1A7**	Extrahepatic UGT isoform active on estrogen catechols and xenobiotics.	Strassburg CP, Oldhafer K, Manns MP, Tukey RH. Differential expression of the UGT1A locus in human liver, biliary, and gastric tissue: identification of UGT1A7 and UGT1A10 transcripts in extrahepatic tissue. Mol Pharmacol. 1997;52(2):212-220. doi:10.1124/mol.52.2.212
**UGT1A9**	Enzyme conjugating a variety of steroids including estradiol metabolites.	Fujiwara R, Yokoi T, Nakajima M. Structure and Protein-Protein Interactions of Human UDP-Glucuronosyltransferases. Front Pharmacol. 2016;7:388. Published 2016 Oct 24. doi:10.3389/fphar.2016.00388
**UGT2B15**	Phase II enzyme that glucuronidates and inactivates steroid hormones (androgens and estrogens).	Harrington WR, Sengupta S, Katzenellenbogen BS. Estrogen regulation of the glucuronidation enzyme UGT2B15 in estrogen receptor-positive breast cancer cells. Endocrinology. 2006;147(8):3843-3850. doi:10.1210/en.2006-0358
**COMT**	Catalyzes O-methylation of catechol-estrogens, reducing oxidative stress and estrogen-mediated DNA damage.	Yager JD. Catechol-O-methyltransferase: characteristics, polymorphisms and role in breast cancer. Drug Discov Today Dis Mech. 2012;9(1-2):e41-e46. doi:10.1016/j.ddmec.2012.10.002
**CYP11B2**	Mitochondrial steroidogenic enzyme producing aldosterone; influences overall steroidogenic flux.	Reddish MJ, Guengerich FP. Human cytochrome P450 11B2 produces aldosterone by a processive mechanism due to the lactol form of the intermediate 18-hydroxycorticosterone. J Biol Chem. 2019;294(35):12975-12991. doi:10.1074/jbc.RA119.009830
**RETSAT**	Retinol saturase involved in retinoid metabolism; linked to redox processes that interact with steroid metabolism.	Pang XY, Wang S, Jurczak MJ, Shulman GI, Moise AR. Retinol saturase modulates lipid metabolism and the production of reactive oxygen species. Arch Biochem Biophys. 2017;633:93-102. doi:10.1016/j.abb.2017.09.009
**DHRS9**	Short-chain dehydrogenase involved in retinoid/steroid oxidation.	Riquelme P, Amodio G, Macedo C, et al. DHRS9 Is a Stable Marker of Human Regulatory Macrophages. Transplantation. 2017;101(11):2731-2738. doi:10.1097/TP.0000000000001814
**RXRB**	Retinoid X receptor beta; forms heterodimers with nuclear receptors, influencing transcriptional programs including those intersecting with steroid metabolism.	Mukherjee R et al. "Retinoid X receptors: biology and therapeutic applications." *Curr Opin Chem Biol.* 2007;11:314–320. PMID: 17574844
**ACAA1**	Peroxisomal thiolase contributing to β-oxidation and lipid substrate processing relevant for steroid production.	Latruffe N, Nicolas-Frances V, Dasari VK, Osumi T. Studies on regulation of the peroxisomal beta-oxidation at the 3-ketothiolase step. Dissection of the rat liver thiolase B gene promoter. Adv Exp Med Biol. 1999;466:253-259. doi:10.1007/0-306-46818-2_30
**PDGFRA**	Receptor tyrosine kinase influencing developmental signalling intersecting with endocrine pathways.	Guérit E, Arts F, Dachy G, Boulouadnine B, Demoulin JB. PDGF receptor mutations in human diseases. Cell Mol Life Sci. 2021;78(8):3867-3881. doi:10.1007/s00018-020-03753-y
**VEGFC**	Growth factor involved in angiogenesis and tissue morphogenesis.	Lee, C., Kim, MJ., Kumar, A. et al. Vascular endothelial growth factor signaling in health and disease: from molecular mechanisms to therapeutic perspectives. Sig Transduct Target Ther 10, 170 (2025). https://doi.org/10.1038/s41392-025-02249-0
**DNM2**	Dynamin-2; regulates membrane trafficking processes relevant to receptor signalling.	Willinger T, Staron M, Ferguson SM, De Camilli P, Flavell RA. Dynamin 2-dependent endocytosis sustains T-cell receptor signaling and drives metabolic reprogramming in T lymphocytes. Proc Natl Acad Sci U S A. 2015;112(14):4423-4428. doi:10.1073/pnas.1504279112
**VCAM1**	Adhesion molecule involved in cell–cell interaction and inflammatory microenvironments.	Wong D, Dorovini-Zis K. Expression of vascular cell adhesion molecule-1 (VCAM-1) by human brain microvessel endothelial cells in primary culture. Microvasc Res. 1995;49(3):325-339. doi:10.1006/mvre.1995.1028
**SOX2**	Transcription factor maintaining progenitor cell programs; interacts with pathways modulating hormone signalling.	Zhou C, Yang X, Sun Y, Yu H, Zhang Y, Jin Y. Comprehensive profiling reveals mechanisms of SOX2-mediated cell fate specification in human ESCs and NPCs. Cell Res. 2016;26(2):171-189. doi:10.1038/cr.2016.15
**P2RY12**	Purinergic receptor involved in GPCR signalling with indirect metabolic roles.	Zhuang W, Liu X, Liu G, et al. Purinergic receptor P2Y12 boosts autoimmune hepatitis through hexokinase 2-dependent glycolysis in T cells. Int J Biol Sci. 2023;19(11):3576-3594. Published 2023 Jul 9. doi:10.7150/ijbs.85133
**MGST2**	Microsomal glutathione S-transferase involved in leukotriene and oxidative metabolism.	Thulasingam M, Orellana L, Nji E, Ahmad S, Rinaldo-Matthis A, Haeggström JZ. Crystal structures of human MGST2 reveal synchronized conformational changes regulating catalysis. Nat Commun. 2021;12(1):1728. Published 2021 Mar 19. doi:10.1038/s41467-021-21924-8

System-level meaning	Proteins
Reduced estrogen-production capacity	CYP19A1 (down), CYP11B2 (up), CYP27B1
Imbalance of active vs inactive estrogens/androgens	HSD17B7 (up), HSD17B10 (up), HSD17B8 (down)
Altered redox-dependent estrogen metabolism	CYP1A1 (up), CYP1B1 (down), CYP3A5 (down), CYB5A (down)
Reprogrammed detoxification and estrogen elimination	UGT1A1 (up), UGT1A8 (up), UGT1A7 (down), UGT1A9 (down), UGT2B15 (down), COMT (up)
Shifted energetic/lipid support for steroidogenesis	ACAA1 (up), ECHS1 (down), AK2 (up), HSDL2 (up)
Perturbed redox environment affecting hormone metabolism	MGST2 (down), ASMT (up), RPP40 (slightly down)
Modified retinoid & hormone-receptor signalling balance	RXRB (up), RETSAT (down), DHRS9 (down)
Disrupted signalling context regulating metabolism	PDGFRA (up), VEGFC (down), VCAM1 (down), DNM2 (down), SOX2 (up), P2RY12 (down)

Estrogen metabolic process

stringId_A	stringId_B	preferredName_A	ensembl_gene_id_A	log2FoldChange_A	preferredName_B	ensembl_gene_id_B	log2FoldChange_B	score
**9606.ENSP00000168216**	9606.ENSP00000369391	HSD17B10	ENSG00000072506	0.29147415617691	RPP40	ENSG00000124787	-0.0352293836860066	0.906
**9606.ENSP00000222982**	9606.ENSP00000307259	CYP3A5	ENSG00000106258	-0.0791904537983588	P2RY12	ENSG00000169313	-0.833156486123551	0.726
**9606.ENSP00000257290**	9606.ENSP00000480043	PDGFRA	ENSG00000134853	0.109697571185514	VEGFC	ENSG00000150630	-1.33976685050209	0.873
**9606.ENSP00000304845**	9606.ENSP00000362525	UGT1A1	ENSG00000241635	0.638864207254496	UGT1A7	ENSG00000244122	-2.6030258915668	0.994
**9606.ENSP00000304845**	9606.ENSP00000378488	UGT1A1	ENSG00000241635	0.638864207254496	CYP1A1	ENSG00000140465	0.120453499068244	0.985
**9606.ENSP00000325822**	9606.ENSP00000379683	CYP11B2	ENSG00000179142	0.228479383556454	CYP19A1	ENSG00000137869	-0.882362025384493	0.947
**9606.ENSP00000341045**	9606.ENSP00000354511	UGT2B15	ENSG00000196620	-0.12564926084382	COMT	ENSG00000093010	0.154391088748595	0.822
**9606.ENSP00000362525**	9606.ENSP00000378488	UGT1A7	ENSG00000244122	-2.6030258915668	CYP1A1	ENSG00000140465	0.120453499068244	0.985
**9606.ENSP00000363794**	9606.ENSP00000363817	HSD17B8	ENSG00000204228	-0.233688080890375	RXRB	ENSG00000204231	0.0479562150646334	0.798
**9606.ENSP00000363794**	9606.ENSP00000393963	HSD17B8	ENSG00000204228	-0.233688080890375	KIFC1	ENSG00000237649	-0.170773448631387	0.82
**9606.ENSP00000370639**	9606.ENSP00000378488	ASMT	ENSG00000196433	0.537937055004421	CYP1A1	ENSG00000140465	0.120453499068244	0.925
**9606.ENSP00000478561**	9606.ENSP00000482639	CYP1B1	ENSG00000138061	-0.104580358025444	MGST2	ENSG00000085871	-0.132064137265605	0.916

score = 0.7

Estrogen biosynthetic process

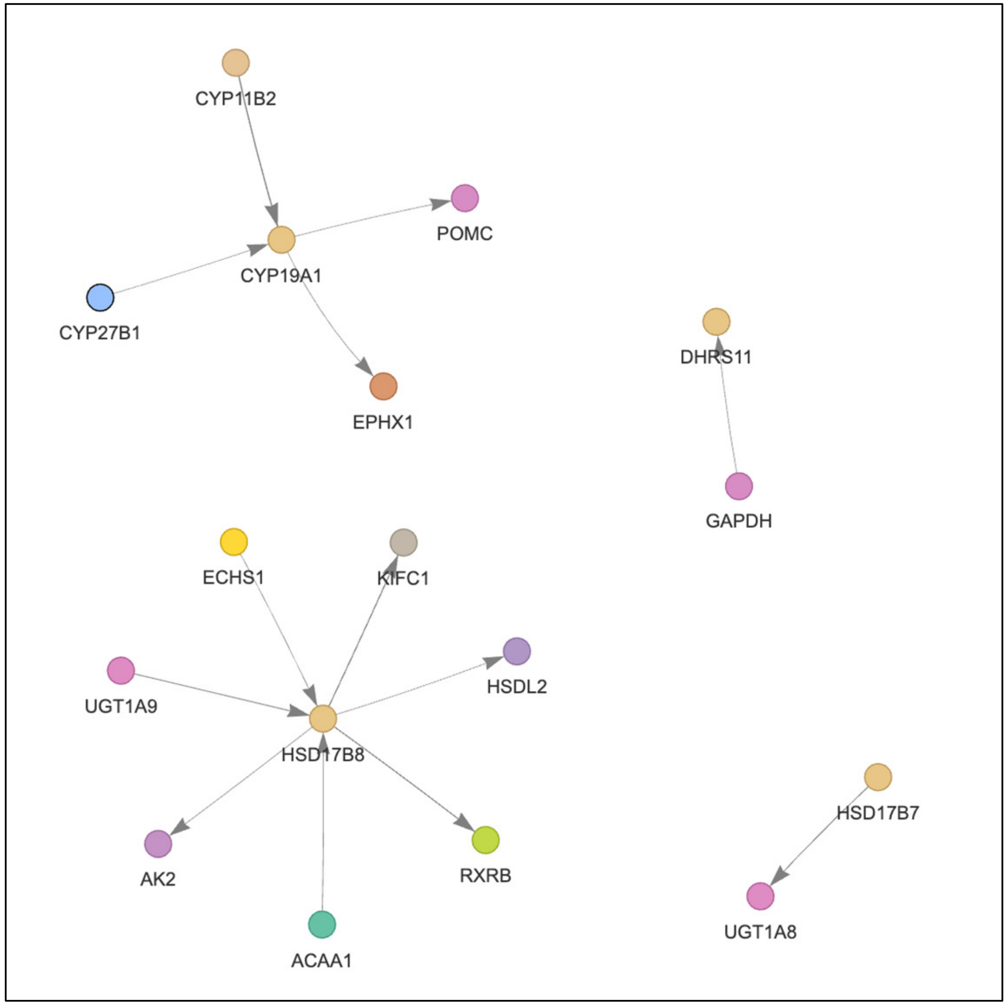

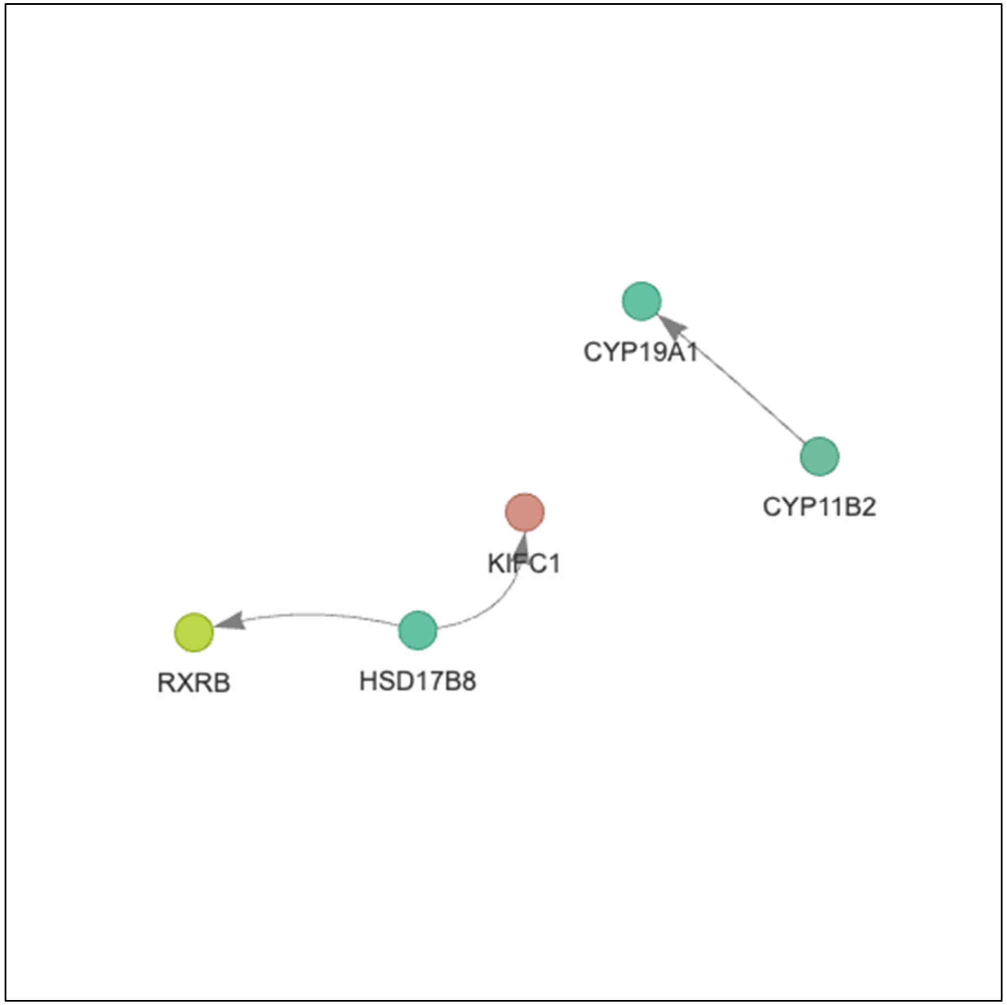

score = 0.4

score = 0.7

Estrogen biosynthetic process

stringId_A	stringId_B	preferredName_A	ensembl_gene_id_A	log2FoldChange_A	preferredName_B	ensembl_gene_id_B	log2FoldChange_B	score
**9606.ENSP00000333664**	9606.ENSP00000363794	ACAA1	ENSG00000060971	0.0533315410354744	HSD17B8	ENSG00000204228	-0.233688080890375	0.552
**9606.ENSP00000346768**	9606.ENSP00000363794	UGT1A9	ENSG00000241119	-0.607976524541623	HSD17B8	ENSG00000204228	-0.233688080890375	0.666
**9606.ENSP00000357535**	9606.ENSP00000363794	ECHS1	ENSG00000127884	-0.343502084653616	HSD17B8	ENSG00000204228	-0.233688080890375	0.477
**9606.ENSP00000363794**	9606.ENSP00000381785	HSD17B8	ENSG00000204228	-0.233688080890375	HSDL2	ENSG00000119471	0.0386167450714514	0.451
**9606.ENSP00000363794**	9606.ENSP00000499935	HSD17B8	ENSG00000204228	-0.233688080890375	AK2	ENSG00000004455	0.225117748162802	0.496
**9606.ENSP00000363794**	9606.ENSP00000363817	HSD17B8	ENSG00000204228	-0.233688080890375	RXRB	ENSG00000204231	0.0479562150646334	0.798
**9606.ENSP00000363794**	9606.ENSP00000393963	HSD17B8	ENSG00000204228	-0.233688080890375	KIFC1	ENSG00000237649	-0.170773448631387	0.82
**9606.ENSP00000228606**	9606.ENSP00000379683	CYP27B1	ENSG00000111012	NA	CYP19A1	ENSG00000137869	-0.882362025384493	0.421
**9606.ENSP00000325822**	9606.ENSP00000379683	CYP11B2	ENSG00000179142	0.228479383556454	CYP19A1	ENSG00000137869	-0.882362025384493	0.947
**9606.ENSP00000379683**	9606.ENSP00000384092	CYP19A1	ENSG00000137869	-0.882362025384493	POMC	ENSG00000115138	-0.247559572823198	0.453
**9606.ENSP00000379683**	9606.ENSP00000480004	CYP19A1	ENSG00000137869	-0.882362025384493	EPHX1	ENSG00000143819	-0.76885137564158	0.477
**9606.ENSP00000380070**	9606.ENSP00000482704	GAPDH	ENSG00000111640	-0.151359811963256	DHRS11	ENSG00000278535	-0.249782697526092	0.464
**9606.ENSP00000254521**	9606.ENSP00000362549	HSD17B7	ENSG00000132196	0.12137210049081	UGT1A8	ENSG00000242366	0.573370146693472	0.677

score = 0.4

Protein	Functional definition	Reference
**CYP19A1**	Aromatase; converts androgens to estrogens, rate-limiting step in estrogen biosynthesis.	Simpson ER, Davis SR. Minireview: aromatase and the regulation of estrogen biosynthesis--some new perspectives. Endocrinology. 2001 Nov;142(11):4589-94. doi: 10.1210/endo.142.11.8547. PMID: 11606422.
**CYP11B2**	Mitochondrial steroidogenic enzyme producing aldosterone; influences overall steroidogenic flux.	Reddish MJ, Guengerich FP. Human cytochrome P450 11B2 produces aldosterone by a processive mechanism due to the lactol form of the intermediate 18-hydroxycorticosterone. J Biol Chem. 2019;294(35):12975-12991. doi:10.1074/jbc.RA119.009830
**HSD17B7**	17β-HSD isoform responsible for reducing estrone to estradiol and participating in androgen/estrogen interconversion.	Thériault JF, Lin SX. The dual sex hormone specificity for human reductive 17β-hydroxysteroid dehydrogenase type 7: Synergistic function in estrogen and androgen control. J Steroid Biochem Mol Biol. 2019;186:61-65. doi:10.1016/j.jsbmb.2018.09.012
**HSDL2**	Oxidoreductase involved in lipid and steroid metabolism, localized to mitochondria/peroxisomes.	Samson N, Bosoi CR, Roy C, et al. HSDL2 links nutritional cues to bile acid and cholesterol homeostasis. Sci Adv. 2024;10(22):eadk9681. doi:10.1126/sciadv.adk9681
**HSD17B8**	Oxidizes active estrogens and androgens to less active forms.	Rotinen M, Celay J, Alonso MM, Arrazola A, Encio I, Villar J. Estradiol induces type 8 17beta-hydroxysteroid dehydrogenase expression: crosstalk between estrogen receptor alpha and C/EBPbeta. J Endocrinol. 2009;200(1):85-92. doi:10.1677/JOE-08-0134
**DHRS11**	Steroid-related dehydrogenase/reductase with oxidoreductase activity relevant to lipid and steroid metabolism.	Endo S, Miyagi N, Matsunaga T, Hara A, Ikari A. Human dehydrogenase/reductase (SDR family) member 11 is a novel type of 17β-hydroxysteroid dehydrogenase. Biochem Biophys Res Commun. 2016;472(1):231-236. doi:10.1016/j.bbrc.2016.01.190
**ECHS1**	Mitochondrial enzyme in fatty-acid β-oxidation; influences energy supply for steroidogenesis.	Burgin H, Sharpe AJ, Nie S, et al. Loss of mitochondrial fatty acid β-oxidation protein short-chain Enoyl-CoA hydratase disrupts oxidative phosphorylation protein complex stability and function. FEBS J. 2023;290(1):225-246. doi:10.1111/febs.16595
**ACAA1**	Peroxisomal thiolase contributing to β-oxidation and lipid substrate processing relevant for steroid production.	Latruffe N, Nicolas-Frances V, Dasari VK, Osumi T. Studies on regulation of the peroxisomal beta-oxidation at the 3-ketothiolase step. Dissection of the rat liver thiolase B gene promoter. Adv Exp Med Biol. 1999;466:253-259. doi:10.1007/0-306-46818-2_30
**AK2**	Mitochondrial adenylate kinase regulating ATP/ADP/AMP balance, supporting energy-dependent steroidogenic processes.	Six E, Lagresle-Peyrou C, Susini S, et al. AK2 deficiency compromises the mitochondrial energy metabolism required for differentiation of human neutrophil and lymphoid lineages. Cell Death Dis. 2015;6(8):e1856. Published 2015 Aug 13. doi:10.1038/cddis.2015.211
**POMC**	Precursor of ACTH; ACTH regulates steroidogenic enzyme expression and adrenal steroid output.	Gallo-Payet N. 60 YEARS OF POMC: Adrenal and extra-adrenal functions of ACTH. J Mol Endocrinol. 2016;56(4):T135-T156. doi:10.1530/JME-15-0257
**EPHX1**	Epoxide hydrolase involved in detoxification and indirectly in estrogen metabolite processing.	Hattori N, Fujiwara H, Maeda M, Fujii S, Ueda M. Epoxide hydrolase affects estrogen production in the human ovary. Endocrinology. 2000;141(9):3353-3365. doi:10.1210/endo.141.9.7682
**CYP27B1**	Mitochondrial 1α-hydroxylase potentially intersecting with steroid and lipid metabolism.	van der Meijden K, van Essen HW, Bloemers FW, Schulten EA, Lips P, Bravenboer N. Regulation of CYP27B1 mRNA Expression in Primary Human Osteoblasts. Calcif Tissue Int. 2016;99(2):164-173. doi:10.1007/s00223-016-0131-9
**RXRB**	Retinoid X receptor beta; heterodimeric nuclear receptor influencing steroid-responsive transcription.	Minucci S, Leid M, Toyama R, et al. Retinoid X receptor (RXR) within the RXR-retinoic acid receptor heterodimer binds its ligand and enhances retinoid-dependent gene expression. Mol Cell Biol. 1997;17(2):644-655. doi:10.1128/MCB.17.2.644
**KIFC1**	Minus-end kinesin motor associated with HSD17B8 cluster; involved in cytoskeletal organization.	She ZY, Pan MY, Tan FQ, Yang WX. Minus end-directed kinesin-14 KIFC1 regulates the positioning and architecture of the Golgi apparatus. Oncotarget. 2017;8(22):36469-36483. doi:10.18632/oncotarget.16863
**UGT1A9**	Enzyme conjugating a variety of steroids including estradiol metabolites.	Fujiwara R, Yokoi T, Nakajima M. Structure and Protein-Protein Interactions of Human UDP-Glucuronosyltransferases. Front Pharmacol. 2016;7:388. Published 2016 Oct 24. doi:10.3389/fphar.2016.00388
**GAPDH**	Housekeeping glycolytic enzyme	Wang J, Yu X, Cao X, et al. GAPDH: A common housekeeping gene with an oncogenic role in pan-cancer. Comput Struct Biotechnol J. 2023;21:4056-4069. Published 2023 Aug 9. doi:10.1016/j.csbj.2023.07.034
**UGT1A8**	Extrahepatic glucuronosyltransferase contributing to estrogen conjugation.	Zhao F, Wang X, Wang Y, et al. The function of uterine UDP-glucuronosyltransferase 1A8 (UGT1A8) and UDP-glucuronosyltransferase 2B7 (UGT2B7) is involved in endometrial cancer based on estrogen metabolism regulation. Hormones (Athens). 2020;19(3):403-412. doi:10.1007/s42000-020-00213-x

System-level meaning	Proteins
Reduced capacity to synthesize estrogens from androgens	CYP19A1 (down), CYP11B2 (up), CYP27B1 (NA), POMC (down), EPHX1 (down)
Altered balance between active and inactive estrogens due to repression of HSD17B8	HSD17B8 (down), HSD17B7 (up), HSDL2 (up), DHRS11 (down), UGT1A8 (up)
Mitochondrial/peroxisomal metabolic adjustments influencing hormone synthesis	ECHS1 (down), ACAA1 (up), AK2 (up)
Modified retinoid - steroid-receptor signalling interactions	RXRB (up), KIFC1 (down)
Increased capacity to conjugate/inactivate estrogen intermediates	UGT1A9 (down), UGT1A8 (up)
Changes in redox balance affecting biosynthetic enzyme efficiency	GAPDH (down), DHRS11 (down)

Estrogen biosynthetic process

stringId_A	stringId_B	preferredName_A	ensembl_gene_id_A	log2FoldChange_A	preferredName_B	ensembl_gene_id_B	log2FoldChange_B	score
9606.ENSP00000325822	9606.ENSP00000379683	CYP11B2	ENSG00000179142	0.228479383556454	CYP19A1	ENSG00000137869	-0.882362025384493	0.947
9606.ENSP00000363794	9606.ENSP00000363817	HSD17B8	ENSG00000204228	-0.233688080890375	RXRB	ENSG00000204231	0.0479562150646334	0.798
9606.ENSP00000363794	9606.ENSP00000393963	HSD17B8	ENSG00000204228	-0.233688080890375	KIFC1	ENSG00000237649	-0.170773448631387	0.82

score = 0.7

Estrogen response element binding

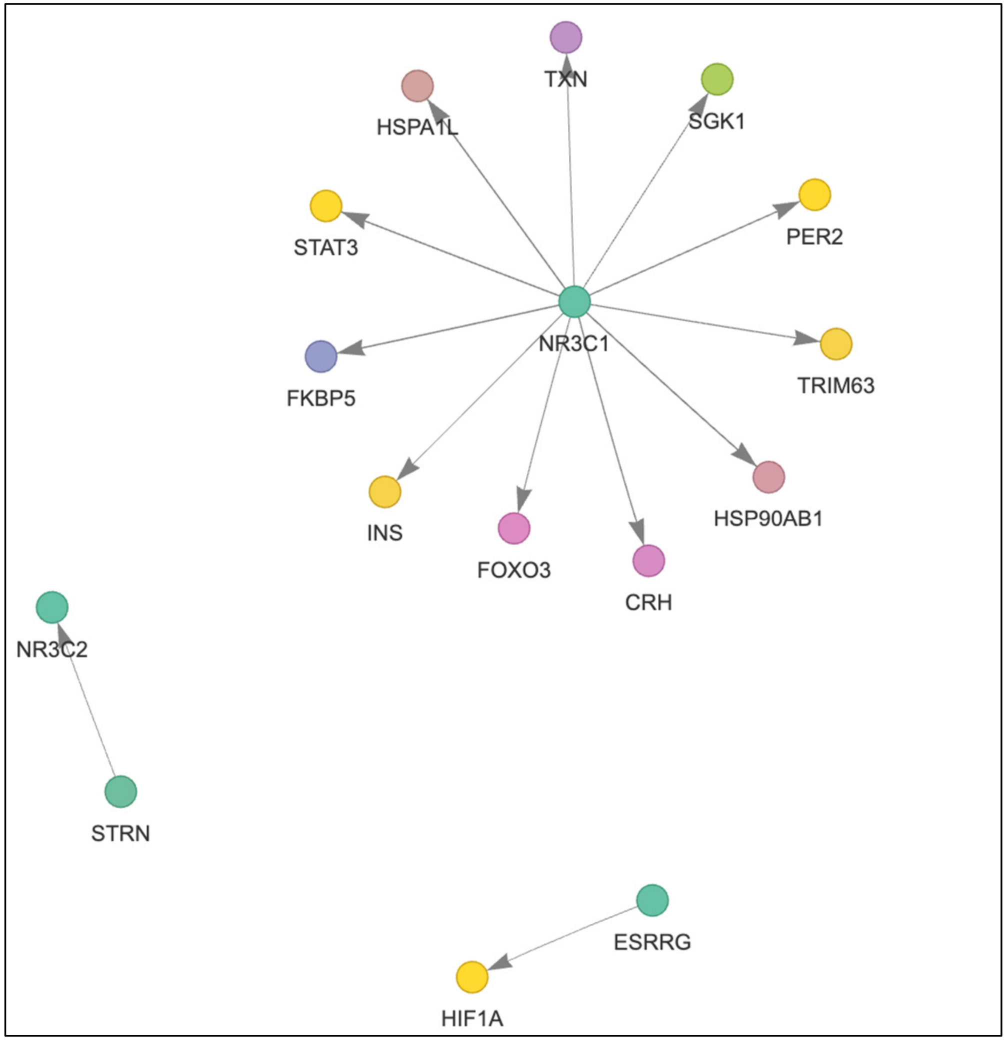

score = 0.4

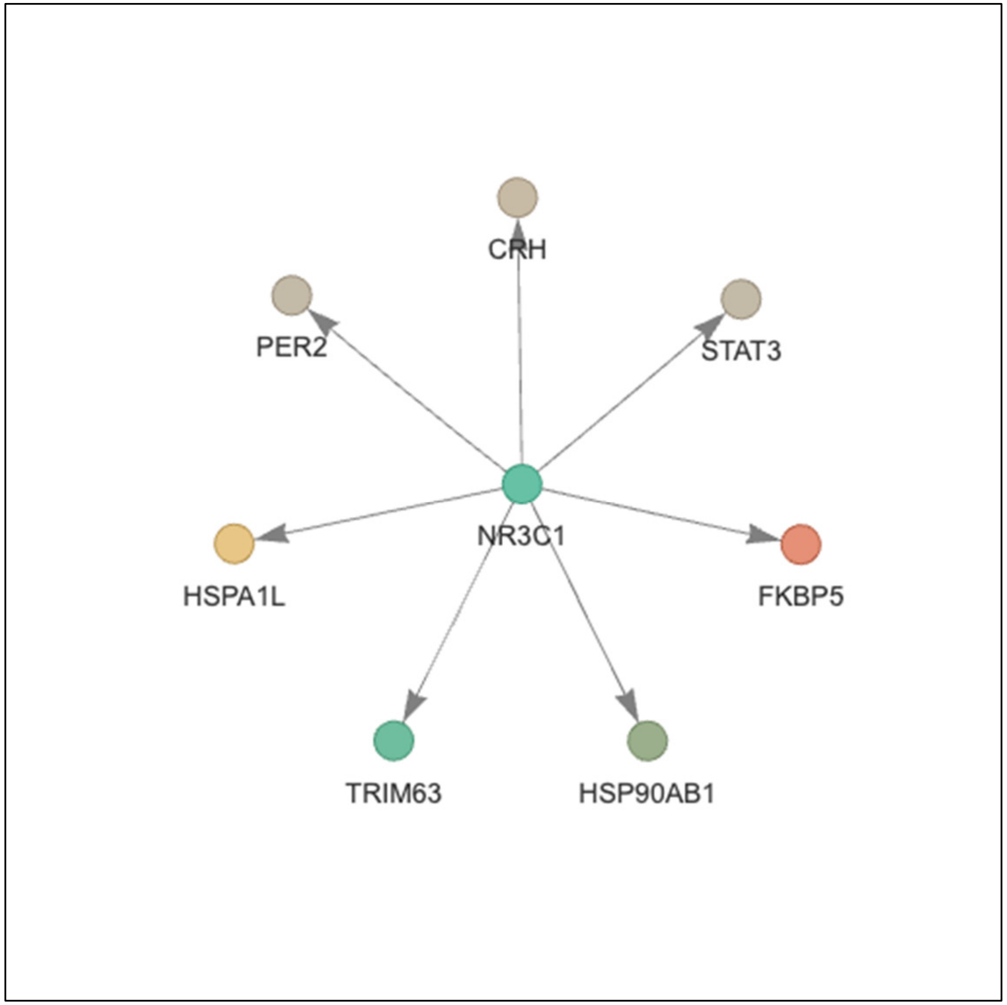

score = 0.7

Estrogen response element binding

stringId_A	stringId_B	preferredName_A	ensembl_gene_id_A	log2FoldChange_A	preferredName_B	ensembl_gene_id_B	log2FoldChange_B	score
**9606.ENSP00000231509**	9606.ENSP00000444810	NR3C1	ENSG00000113580	0.19869114523122	FKBP5	ENSG00000096060	-0.50510153301164	0.999
**9606.ENSP00000231509**	9606.ENSP00000360609	NR3C1	ENSG00000113580	0.19869114523122	HSP90AB1	ENSG00000096384	-0.129105267756862	0.998
**9606.ENSP00000231509**	9606.ENSP00000364805	NR3C1	ENSG00000113580	0.19869114523122	HSPA1L	ENSG00000204390	0.241281794241872	0.985
**9606.ENSP00000231509**	9606.ENSP00000264657	NR3C1	ENSG00000113580	0.19869114523122	STAT3	ENSG00000168610	0.913072719593478	0.981
**9606.ENSP00000231509**	9606.ENSP00000254657	NR3C1	ENSG00000113580	0.19869114523122	PER2	ENSG00000132326	-2.1704736958566	0.905
**9606.ENSP00000231509**	9606.ENSP00000276571	NR3C1	ENSG00000113580	0.19869114523122	CRH	ENSG00000147571	0.18035229790573	0.872
**9606.ENSP00000231509**	9606.ENSP00000363390	NR3C1	ENSG00000113580	0.19869114523122	TRIM63	ENSG00000158022	-0.452321000844343	0.828
**9606.ENSP00000231509**	9606.ENSP00000339527	NR3C1	ENSG00000113580	0.19869114523122	FOXO3	ENSG00000118689	0.220673499685745	0.686
**9606.ENSP00000231509**	9606.ENSP00000356832	NR3C1	ENSG00000113580	0.19869114523122	SGK1	ENSG00000118515	0.25124412351627	0.64
**9606.ENSP00000231509**	9606.ENSP00000380432	NR3C1	ENSG00000113580	0.19869114523122	INS	ENSG00000254647	-1.20142421383256	0.629
**9606.ENSP00000231509**	9606.ENSP00000363641	NR3C1	ENSG00000113580	0.19869114523122	TXN	ENSG00000136810	-0.184863798411965	0.626
**9606.ENSP00000263918**	9606.ENSP00000350815	STRN	ENSG00000115808	-0.803064322835722	NR3C2	ENSG00000151623	0.582934046748962	0.553
**9606.ENSP00000355904**	9606.ENSP00000437955	ESRRG	ENSG00000196482	-0.074669885238237	HIF1A	ENSG00000100644	-0.15475077683914	0.539

score = 0.4

Protein	Functional definition	Reference
**NR3C1**	Nuclear receptor that binds glucocorticoid response elements and shares co-regulators/chaperones with estrogen receptors; major hub in steroid receptor crosstalk.	Oakley RH, Cidlowski JA. The biology of the glucocorticoid receptor: new signaling mechanisms in health and disease. J Allergy Clin Immunol. 2013;132(5):1033-1044. doi:10.1016/j.jaci.2013.09.007
**FKBP5**	Co-chaperone regulating NR3C1 sensitivity and nuclear translocation; modulates steroid receptor responsiveness.	Binder EB. The role of FKBP5, a co-chaperone of the glucocorticoid receptor in the pathogenesis and therapy of affective and anxiety disorders. Psychoneuroendocrinology. 2009;34 Suppl 1:S186-S195. doi:10.1016/j.psyneuen.2009.05.021
**HSP90AB1**	Heat-shock chaperone essential for the folding and activation of steroid receptors, including GR and ER.	Sanchez ER. Chaperoning steroidal physiology: lessons from mouse genetic models of Hsp90 and its cochaperones. Biochim Biophys Acta. 2012;1823(3):722-729. doi:10.1016/j.bbamcr.2011.11.006
**HSPA1L**	Heat-shock protein participating in chaperone cycles of nuclear hormone receptors.	Tian G, Hu C, Yun Y, et al. Dual roles of HSP70 chaperone HSPA1 in quality control of nascent and newly synthesized proteins. EMBO J. 2021;40(13):e106183. doi:10.15252/embj.2020106183
**SGK1**	Serine/threonine kinase induced by glucocorticoids; integrates metabolic and steroid receptor signaling.	Szmulewitz RZ, Chung E, Al-Ahmadie H, et al. Serum/glucocorticoid-regulated kinase 1 expression in primary human prostate cancers. Prostate. 2012;72(2):157-164. doi:10.1002/pros.21416
**STAT3**	Transcription factor linking cytokine/inflammatory signaling to nuclear receptor pathways, including ER and GR crosstalk.	Hillmer EJ, Zhang H, Li HS, Watowich SS. STAT3 signaling in immunity. Cytokine Growth Factor Rev. 2016;31:1-15. doi:10.1016/j.cytogfr.2016.05.001
**FOXO3**	Forkhead transcription factor interacting with nuclear receptor signaling and modulating stress/metabolic pathways.	Christian M, Lam EW, Wilson MS, Brosens JJ. FOXO transcription factors and their role in disorders of the female reproductive tract. Curr Drug Targets. 2011;12(9):1291-1302. doi:10.2174/138945011796150253
**PER2**	Core circadian regulator influencing nuclear receptor transcriptional cycles, including steroid receptor rhythms.	Gery S, Virk RK, Chumakov K, Yu A, Koeffler HP. The clock gene Per2 links the circadian system to the estrogen receptor. Oncogene. 2007;26(57):7916-7920. doi:10.1038/sj.onc.1210585
**CRH**	Hypothalamic hormone regulating HPA axis output; interacts indirectly with glucocorticoid receptor signaling.	Yanovski JA, Cutler GB Jr, Chrousos GP, Nieman LK. The dexamethasone-suppressed corticotropin-releasing hormone stimulation test differentiates mild Cushing's disease from normal physiology. J Clin Endocrinol Metab. 1998;83(2):348-352. doi:10.1210/jcem.83.2.4568
**INS**	Metabolic hormone modulating nuclear receptor activity and intersecting with ER/GR signaling pathways.	Isenovic ER, Zakula Z, Koricanac G, Ribarac-Stepic N. Insulin modulates rat liver glucocorticoid receptor. Acta Biol Hung. 2006;57(1):37-48. doi:10.1556/ABiol.57.2006.1.4
**TXN**	Redox-regulating protein affecting nuclear receptor function through control of oxidative environment.	Makino Y, Yoshikawa N, Okamoto K, et al. Direct association with thioredoxin allows redox regulation of glucocorticoid receptor function. J Biol Chem. 1999;274(5):3182-3188. doi:10.1074/jbc.274.5.3182
**NR3C2**	Nuclear receptor closely related to NR3C1, sharing co-regulators and influencing steroid receptor crosstalk.	Viengchareun S, Le Menuet D, Martinerie L, Munier M, Pascual-Le Tallec L, Lombès M. The mineralocorticoid receptor: insights into its molecular and (patho)physiological biology. *Nucl Recept Signal*. 2007;5:e012. Published 2007 Nov 30. doi:10.1621/nrs.05012
**STRN**	Scaffolding protein modulating MR signaling and receptor complex assembly.	Stone IB, Green JAEM, Koefoed AW, et al. Striatin genotype-based, mineralocorticoid receptor antagonist-driven clinical trial: study rationale and design. Pharmacogenet Genomics. 2021;31(4):83-88. doi:10.1097/FPC.0000000000000425
**TRIM63**	Muscle-specific E3 ubiquitin ligase regulated by steroid hormone signaling; interacts indirectly with GR-dependent metabolic adaptation.	Baehr LM, Hughes DC, Lynch SA, et al. Identification of the MuRF1 Skeletal Muscle Ubiquitylome Through Quantitative Proteomics. Function (Oxf). 2021;2(4):zqab029. Published 2021 May 19. doi:10.1093/function/zqab029
**HIF1A**	Hypoxia-inducible transcription factor influencing metabolic and stress pathways that intersect with nuclear receptor regulation.	Lim J, Kim HI, Bang Y, Seol W, Choi HS, Choi HJ. Hypoxia-inducible factor-1α upregulates tyrosine hydroxylase and dopamine transporter by nuclear receptor ERRγ in SH-SY5Y cells. Neuroreport. 2015;26(6):380-386. doi:10.1097/WNR.0000000000000356
**ESRRG**	Estrogen-related receptor γ; orphan nuclear receptor participating in metabolic gene regulation and receptor crosstalk.	Kim DK, Ryu D, Koh M, et al. Orphan nuclear receptor estrogen-related receptor γ (ERRγ) is key regulator of hepatic gluconeogenesis. J Biol Chem. 2012;287(26):21628-21639. doi:10.1074/jbc.M111.315168

System-level meaning	Proteins
Altered stress–hormone response and transcriptional regulation	NR3C1 (up), FKBP5 (down), HSP90AB1 (down), HSPA1L (up)
Enhanced activation of downstream transcription factors	STAT3 (up), FOXO3 (up)
Disruption of circadian control of hormone-responsive pathways	PER2 (down), TXN (down)
Perturbation of insulin-linked metabolic signalling	INS (down), SGK1 (up)
Perturbed neuroendocrine hormone output	CRH (up), TRIM63 (down)
Modified interplay between GR and MR pathways	NR3C2 (up), STRN (down)
Shift in oxygen-responsive and estrogen-related transcription	ESRRG (down), HIF1A (down)

Estrogen response element binding

stringId_A	stringId_B	preferredName_A	ensembl_gene_id_A	log2FoldChange_A	preferredName_B	ensembl_gene_id_B	log2FoldChange_B	score
9606.ENSP00000231509	9606.ENSP00000444810	NR3C1	ENSG00000113580	0.19869114523122	FKBP5	ENSG00000096060	-0.50510153301164	0.999
9606.ENSP00000231509	9606.ENSP00000360609	NR3C1	ENSG00000113580	0.19869114523122	HSP90AB1	ENSG00000096384	-0.129105267756862	0.998
9606.ENSP00000231509	9606.ENSP00000364805	NR3C1	ENSG00000113580	0.19869114523122	HSPA1L	ENSG00000204390	0.241281794241872	0.985
9606.ENSP00000231509	9606.ENSP00000264657	NR3C1	ENSG00000113580	0.19869114523122	STAT3	ENSG00000168610	0.913072719593478	0.981
9606.ENSP00000231509	9606.ENSP00000254657	NR3C1	ENSG00000113580	0.19869114523122	PER2	ENSG00000132326	-2.1704736958566	0.905
9606.ENSP00000231509	9606.ENSP00000276571	NR3C1	ENSG00000113580	0.19869114523122	CRH	ENSG00000147571	0.18035229790573	0.872
9606.ENSP00000231509	9606.ENSP00000363390	NR3C1	ENSG00000113580	0.19869114523122	TRIM63	ENSG00000158022	-0.452321000844343	0.828

score = 0.7

Estrogen receptor signalling pathway

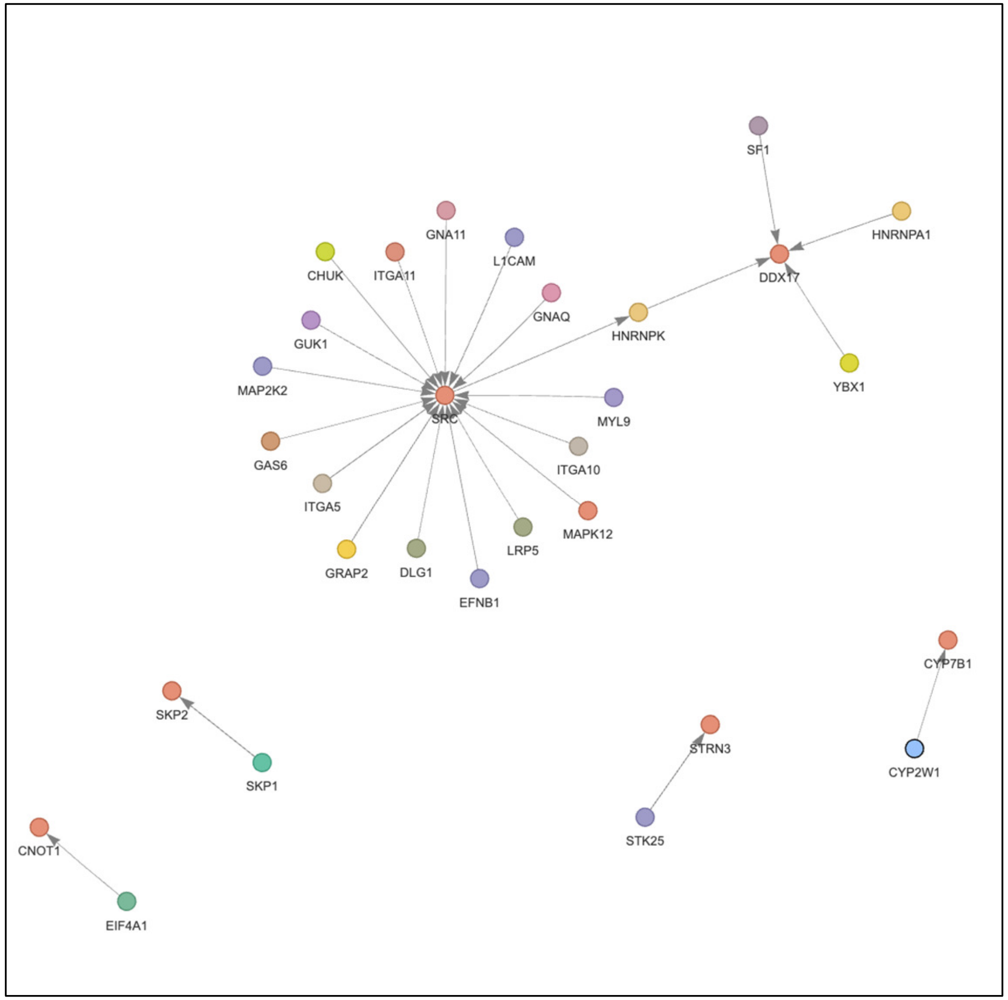

score = 0.4

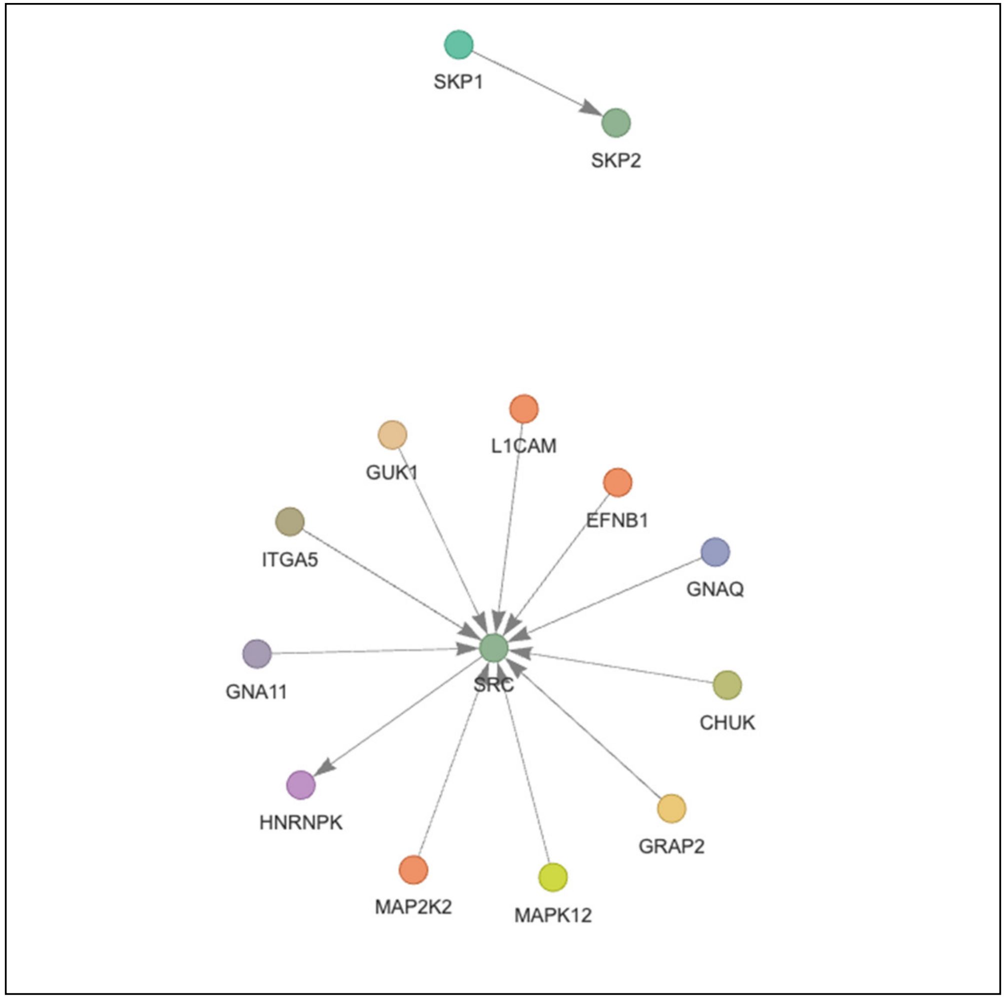

score = 0.7

Estrogen receptor signalling pathway

stringId_A	stringId_B	preferredName_A	ensembl_gene_id_A	log2FoldChange_A	preferredName_B	ensembl_gene_id_B	log2FoldChange_B	score
**9606.ENSP00000231487**	9606.ENSP00000274255	SKP1	ENSG00000113558	0.192330871345847	SKP2	ENSG00000145604	0.873448647127982	0.999
**9606.ENSP00000325748**	9606.ENSP00000350071	STK25	ENSG00000115694	-0.582500016367372	STRN3	ENSG00000196792	0.0307174518118815	0.998
**9606.ENSP00000293379**	9606.ENSP00000362680	ITGA5	ENSG00000161638	0.124528438182347	SRC	ENSG00000197122	0.014167751832404	0.949
**9606.ENSP00000339186**	9606.ENSP00000362680	GRAP2	ENSG00000100351	0.432123293132431	SRC	ENSG00000197122	0.014167751832404	0.909
**9606.ENSP00000341826**	9606.ENSP00000380033	HNRNPA1	ENSG00000135486	0.246390072811463	DDX17	ENSG00000100201	0.190788160408698	0.865
**9606.ENSP00000286548**	9606.ENSP00000362680	GNAQ	ENSG00000156052	0.136903888530539	SRC	ENSG00000197122	0.014167751832404	0.86
**9606.ENSP00000366604**	9606.ENSP00000380033	SF1	ENSG00000168066	0.66044542373951	DDX17	ENSG00000100201	0.190788160408698	0.859
**9606.ENSP00000359077**	9606.ENSP00000362680	L1CAM	ENSG00000198910	0.231508359117218	SRC	ENSG00000197122	0.014167751832404	0.832
**9606.ENSP00000365458**	9606.ENSP00000380033	HNRNPK	ENSG00000165119	0.126828305597743	DDX17	ENSG00000100201	0.190788160408698	0.822
**9606.ENSP00000362680**	9606.ENSP00000365458	SRC	ENSG00000197122	0.014167751832404	HNRNPK	ENSG00000165119	0.126828305597743	0.817
**9606.ENSP00000204961**	9606.ENSP00000362680	EFNB1	ENSG00000090776	0.77316859545731	SRC	ENSG00000197122	0.014167751832404	0.806
**9606.ENSP00000359424**	9606.ENSP00000362680	CHUK	ENSG00000213341	0.138622450678004	SRC	ENSG00000197122	0.014167751832404	0.75
**9606.ENSP00000215659**	9606.ENSP00000362680	MAPK12	ENSG00000188130	1.87975275321622	SRC	ENSG00000197122	0.014167751832404	0.743
**9606.ENSP00000355689**	9606.ENSP00000362680	GUK1	ENSG00000143774	-0.590574395163386	SRC	ENSG00000197122	0.014167751832404	0.727
**9606.ENSP00000262948**	9606.ENSP00000362680	MAP2K2	ENSG00000126934	-0.294016138763207	SRC	ENSG00000197122	0.014167751832404	0.716
**9606.ENSP00000078429**	9606.ENSP00000362680	GNA11	ENSG00000088256	-0.640672184905867	SRC	ENSG00000197122	0.014167751832404	0.713
**9606.ENSP00000327290**	9606.ENSP00000362680	ITGA11	ENSG00000137809	1.37667200784309	SRC	ENSG00000197122	0.014167751832404	0.697
**9606.ENSP00000358310**	9606.ENSP00000362680	ITGA10	ENSG00000143127	-0.0955021140614719	SRC	ENSG00000197122	0.014167751832404	0.697
**9606.ENSP00000293831**	9606.ENSP00000320949	EIF4A1	ENSG00000161960	-0.0620524636091356	CNOT1	ENSG00000125107	0.206556825152838	0.695
**9606.ENSP00000279022**	9606.ENSP00000362680	MYL9	ENSG00000101335	0.389695067495551	SRC	ENSG00000197122	0.014167751832404	0.695
**9606.ENSP00000361626**	9606.ENSP00000380033	YBX1	ENSG00000065978	-0.259080766205886	DDX17	ENSG00000100201	0.190788160408698	0.682
**9606.ENSP00000345731**	9606.ENSP00000362680	DLG1	ENSG00000075711	-0.129727651679456	SRC	ENSG00000197122	0.014167751832404	0.655
**9606.ENSP00000310149**	9606.ENSP00000310721	CYP2W1	ENSG00000073067	NA	CYP7B1	ENSG00000172817	0.134064828662285	0.654
**9606.ENSP00000294304**	9606.ENSP00000362680	LRP5	ENSG00000162337	0.0214105377971577	SRC	ENSG00000197122	0.014167751832404	0.636
**9606.ENSP00000331831**	9606.ENSP00000362680	GAS6	ENSG00000183087	-0.147338897959435	SRC	ENSG00000197122	0.014167751832404	0.634

score = 0.4

Protein	Functional definition	Reference
**SRC**	Central non-genomic estrogen receptor signaling mediator; ER activation rapidly triggers SRC-dependent phosphorylation cascades involved in adhesion and kinase signaling.	Meyer MR, Haas E, Prossnitz ER, Barton M. Non-genomic regulation of vascular cell function and growth by estrogen. Mol Cell Endocrinol. 2009;308(1-2):9-16. doi:10.1016/j.mce.2009.03.009
**ITGA11**	Integrin α11; adhesion receptor that can activate SRC-dependent signaling pathways affecting cell migration and extracellular matrix interactions.	Bansal R, Nakagawa S, Yazdani S, et al. Integrin alpha 11 in the regulation of the myofibroblast phenotype: implications for fibrotic diseases. Exp Mol Med. 2017;49(11):e396. Published 2017 Nov 17. doi:10.1038/emm.2017.213
**ITGA5**	Integrin α5; interacts with fibronectin to activate focal adhesion kinases and SRC-mediated pathways relevant to ER non-genomic signaling.	Zhao X, Guan JL. Focal adhesion kinase and its signaling pathways in cell migration and angiogenesis. Adv Drug Deliv Rev. 2011;63(8):610-615. doi:10.1016/j.addr.2010.11.001
**ITGA10**	Integrin α10; collagen-binding integrin contributing to adhesion/migration signaling cascades that can interface with SRC.	Liang C, Liu X, Liu C, Xu Y, Geng W, Li J. Integrin α10 regulates adhesion, migration, and osteogenic differentiation of alveolar bone marrow mesenchymal stem cells in type 2 diabetic patients who underwent dental implant surgery. Bioengineered. 2022;13(5):13252-13268. doi:10.1080/21655979.2022.2079254
**EFNB1 (Ephrin-B1)**	Membrane-bound ligand activating Eph receptors and SRC-family kinase signaling; modulates adhesion and cytoskeletal dynamics.	Atapattu L, Lackmann M, Janes PW. The role of proteases in regulating Eph/ephrin signaling. Cell Adh Migr. 2014;8(4):294-307. doi:10.4161/19336918.2014.970026
**L1CAM**	Cell adhesion molecule activating downstream SRC/MAPK pathways and modulating neuronal and non-neuronal migration.	Whittard JD, Sakurai T, Cassella MR, Gazdoiu M, Felsenfeld DP. MAP kinase pathway-dependent phosphorylation of the L1-CAM ankyrin binding site regulates neuronal growth. Mol Biol Cell. 2006;17(6):2696-2706. doi:10.1091/mbc.e06-01-0090
**MAPK12 (p38γ)**	Member of p38 MAPK family; participates in stress and receptor-mediated kinase signaling pathways downstream of SRC.	Han J, Wu J, Silke J. An overview of mammalian p38 mitogen-activated protein kinases, central regulators of cell stress and receptor signaling. F1000Res. 2020;9:F1000 Faculty Rev-653. Published 2020 Jun 29. doi:10.12688/f1000research.22092.1
**MAP2K2 (MEK2)**	Dual-specificity kinase in the ERK/MAPK cascade; integrates upstream SRC and receptor signaling.	Guo YJ, Pan WW, Liu SB, Shen ZF, Xu Y, Hu LL. ERK/MAPK signalling pathway and tumorigenesis. Exp Ther Med. 2020;19(3):1997-2007. doi:10.3892/etm.2020.8454
**CHUK (IKKα)**	Kinase involved in NF-κB activation; interacts with growth factor/SRC pathways and modulates nuclear receptor transcription.	Paul A, Edwards J, Pepper C, Mackay S. Inhibitory-κB Kinase (IKK) α and Nuclear Factor-κB (NFκB)-Inducing Kinase (NIK) as Anti-Cancer Drug Targets. Cells. 2018;7(10):176. Published 2018 Oct 20. doi:10.3390/cells7100176
**GNAQ**	G-protein α-subunit triggering PLCβ/Ca^{2+} pathways, which can interface with SRC and ER cross-talk signaling.	Cramer, Henning & Schmenger, Kai & Heinrich, Khristina & Horstmeyer, Angelika & Böning, Hilke & Breit, Andreas & Piiper, Albrecht & Lundstrom, Kenneth & Müller-Esterl, Werner & Schröder, Christian. (2001). Coupling of endothelin receptors to the ERK/MAP kinase pathway. European journal of biochemistry / FEBS. 268. 5449-59. 10.1046/j.0014-2956.2001.02486.x.
**GNA11**	G-protein α-subunit similar to GNAQ; mediates receptor signaling impacting MAPK/SRC pathways.	Silva-Rodríguez P, Fernández-Díaz D, Bande M, Pardo M, Loidi L, Blanco-Teijeiro MJ. GNAQ and GNA11 Genes: A Comprehensive Review on Oncogenesis, Prognosis and Therapeutic Opportunities in Uveal Melanoma. Cancers (Basel). 2022;14(13):3066. Published 2022 Jun 22. doi:10.3390/cancers14133066
**DDX17**	RNA helicase functioning as ER co-regulator, modulating ERα chromatin binding and transcriptional output.	Samaan S, Tranchevent LC, Dardenne E, et al. The Ddx5 and Ddx17 RNA helicases are cornerstones in the complex regulatory array of steroid hormone-signaling pathways. Nucleic Acids Res. 2014;42(4):2197-2207. doi:10.1093/nar/gkt1216
**HNRNPA1**	RNA-binding protein involved in splicing; interacts with chromatin and nuclear receptor–associated transcripts.	Clarke JP, Thibault PA, Salapa HE, Levin MC. A Comprehensive Analysis of the Role of hnRNP A1 Function and Dysfunction in the Pathogenesis of Neurodegenerative Disease. Front Mol Biosci. 2021;8:659610. Published 2021 Apr 12. doi:10.3389/fmolb.2021.659610
**HNRNPK**	RNA- and DNA-binding regulatory protein involved in chromatin remodeling and transcriptional control in nuclear receptor signaling.	Xu Y, Wu W, Han Q, et al. Post-translational modification control of RNA-binding protein hnRNPK function. Open Biol. 2019;9(3):180239. doi:10.1098/rsob.180239
**SF1**	Nuclear receptor regulating steroidogenic gene expression and acting as co-regulatory partner in ER-associated transcription.	Ferraz-de-Souza B, Lin L, Achermann JC. Steroidogenic factor-1 (SF-1, NR5A1) and human disease. Mol Cell Endocrinol. 2011;336(1-2):198-205. doi:10.1016/j.mce.2010.11.006
**YBX1**	Multifunctional RNA- and DNA-binding protein affecting transcription and mRNA stability; interacts with ER signaling networks.	Sangermano, Felicia & Delicato, Antonella & Calabrò, Viola. (2020). Y box binding protein 1 (YB-1) oncoprotein at the hub of DNA proliferation, damage and cancer progression. Biochimie. 179. 205-216. 10.1016/j.biochi.2020.10.004.
**SKP1**	Component of SCF ubiquitin ligase complex; regulates turnover of nuclear cofactors including ER-associated proteins.	Xie J, Jin Y, Wang G. The role of SCF ubiquitin-ligase complex at the beginning of life. Reprod Biol Endocrinol. 2019;17(1):101. Published 2019 Nov 28. doi:10.1186/s12958-019-0547-y
GRAP2	*cytosolic mediator capable of facilitating integration between membrane receptors and MAPK pathways*	Ma W, Xia C, Ling P, et al. Leukocyte-specific adaptor protein Grap2 interacts with hematopoietic progenitor kinase 1 (HPK1) to activate JNK signaling pathway in T lymphocytes. Oncogene. 2001;20(14):1703-1714. doi:10.1038/sj.onc.1204224
**SKP2**	F-box protein of SCF complex; mediates ubiquitination of regulatory proteins involved in ER signaling cycles.	Cai Z, Moten A, Peng D, et al. The Skp2 Pathway: A Critical Target for Cancer Therapy. Semin Cancer Biol. 2020;67(Pt 2):16-33. doi:10.1016/j.semcancer.2020.01.013
**CNOT1**	Core scaffold of the CCR4–NOT complex controlling mRNA decay and transcriptional repression, relevant for nuclear receptor regulation.	Winkler, G.S., Mulder, K.W., Bardwell, V.J. et al. Human Ccr4-Not complex is a ligand-dependent repressor of nuclear receptor-mediated transcription. EMBO J 25, 3089–3099 (2006). https://doi.org/10.1038/sj.emboj.7601194
**GUK1**	Guanylate kinase involved in nucleotide metabolism and indirectly supporting transcriptional processes.	Schneider JL, Kurmi K, Dai Y, et al. GUK1 activation is a metabolic liability in lung cancer. Cell. 2025;188(5):1248-1264.e23. doi:10.1016/j.cell.2025.01.024
**MYL9**	Myosin light chain involved in cytoskeletal remodeling and cell contractility, influenced by SRC signaling.	Brito C, Pereira JM, Mesquita FS, Cabanes D, Sousa S. Src-Dependent NM2A Tyrosine Phosphorylation Regulates Actomyosin Remodeling. Cells. 2023; 12(14):1871. https://doi.org/10.3390/cells12141871
**GAS6**	Ligand for AXL/TYRO3/MERTK receptor tyrosine kinases; activates downstream SRC–MAPK signalling and modulates cell survival and migration.	Zhai X, Pu D, Wang R, et al. Gas6/AXL pathway: immunological landscape and therapeutic potential. Front Oncol. 2023;13:1121130. Published 2023 May 10. doi:10.3389/fonc.2023.1121130
**DLG1**	Scaffolding protein of cell–cell junctions; organizes signalling complexes including SRC-linked adhesion and polarity pathways.	Walch L. Emerging role of the scaffolding protein Dlg1 in vesicle trafficking. Traffic. 2013;14(9):964-973. doi:10.1111/tra.12089
**LRP5**	Co-receptor in Wnt signalling; interfaces with integrin/SRC pathways influencing cytoskeletal dynamics and migration.	Hong J, Xie Z, Yang Z, et al. Inactivation of Wnt-LRP5 signaling suppresses the proliferation and migration of ovarian cancer cells. Transl Cancer Res. 2021;10(5):2277-2285. doi:10.21037/tcr-20-3462
**EIF4A1**	DEAD-box helicase required for translation initiation; modulates availability of ER/SRC-regulated transcripts.	Huang J, Zhang L, Yang R, et al. Eukaryotic translation initiation factor 4A1 in the pathogenesis and treatment of cancers. Front Mol Biosci. 2023;10:1289650. Published 2023 Nov 9. doi:10.3389/fmolb.2023.1289650
**CYP2W1**	Extrahepatic cytochrome P450 enzyme capable of metabolizing xenobiotics and lipid-derived ligands; indirectly interacts with steroid receptor signalling contexts.	Zhao Y, Wan D, Yang J, Hammock BD, Ortiz de Montellano PR. Catalytic Activities of Tumor-Specific Human Cytochrome P450 CYP2W1 Toward Endogenous Substrates. Drug Metab Dispos. 2016;44(5):771-780. doi:10.1124/dmd.116.069633
**CYP7B1**	Hydroxylase metabolizing steroidal oxysterols and neurosteroids; modulates availability of ligands intersecting with ER and SRC-dependent pathways.	Yantsevich AV, Dichenko YV, Mackenzie F, et al. Human steroid and oxysterol 7α-hydroxylase CYP7B1: substrate specificity, azole binding and misfolding of clinically relevant mutants. *FEBS J.* 2014;281(6):1700-1713. doi:10.1111/febs.12733
**STRN3**	Scaffolding component of the STRIPAK complex connecting kinases (including MST/LATS) to SRC-linked cytoskeletal and signalling modules.	Li AX, Martin TA, Lane J, Jiang WG. Cellular Impacts of Striatins and the STRIPAK Complex and Their Roles in the Development and Metastasis in Clinical Cancers (Review). Cancers (Basel). 2023;16(1):76. Published 2023 Dec 22. doi:10.3390/cancers16010076

System-level meaning	Proteins
Altered non-genomic ER signalling through adhesion and MAPK / G-protein pathways	SRC (unchanged), ITGA11 (up), ITGA5 (up), ITGA10 (down), EFNB1 (up), L1CAM (up), MAPK12 (up), MAP2K2 (down), CHUK (up), GNAQ (up), GNA11 (down), GAS6 (down), MYL9 (up), GUK1 (down), LRP5 (up), DLG1 (down), GRAP2 (up)
Modulation of ER-dependent transcription via RNA-binding cofactors	DDX17 (up), HNRNPA1 (up), HNRNPK (up), SF1 (up), YBX1 (down)
Adjusted degradation of ER-associated cofactors	SKP1 (up), SKP2 (up)
Altered availability of ER-responsive transcripts	EIF4A1 (down), CNOT1 (up)
Reorganisation of polarity and tension pathways affecting ER scaffolding	STK25 (down), STRN3 (up)
Changes in steroid / oxysterol metabolism that can modulate ER ligand pools	CYP2W1 (NA), CYP7B1 (up)

Estrogen receptor signalling pathway

stringId_A	stringId_B	preferredName_A	ensembl_gene_id_A	log2FoldChange_A	preferredName_B	ensembl_gene_id_B	log2FoldChange_B	score
9606.ENSP00000231487	9606.ENSP00000274255	SKP1	ENSG00000113558	0.192330871345847	SKP2	ENSG00000145604	0.873448647127982	0.999
9606.ENSP00000293379	9606.ENSP00000362680	ITGA5	ENSG00000161638	0.124528438182347	SRC	ENSG00000197122	0.014167751832404	0.949
9606.ENSP00000339186	9606.ENSP00000362680	GRAP2	ENSG00000100351	0.432123293132431	SRC	ENSG00000197122	0.014167751832404	0.909
9606.ENSP00000286548	9606.ENSP00000362680	GNAQ	ENSG00000156052	0.136903888530539	SRC	ENSG00000197122	0.014167751832404	0.860
9606.ENSP00000359077	9606.ENSP00000362680	L1CAM	ENSG00000198910	0.231508359117218	SRC	ENSG00000197122	0.014167751832404	0.832
9606.ENSP00000362680	9606.ENSP00000365458	SRC	ENSG00000197122	0.014167751832404	HNRNPK	ENSG00000165119	0.126828305597743	0.817
9606.ENSP00000204961	9606.ENSP00000362680	EFNB1	ENSG00000090776	0.77316859545731	SRC	ENSG00000197122	0.014167751832404	0.806
9606.ENSP00000359424	9606.ENSP00000362680	CHUK	ENSG00000213341	0.138622450678004	SRC	ENSG00000197122	0.014167751832404	0.750
9606.ENSP00000215659	9606.ENSP00000362680	MAPK12	ENSG00000188130	1.87975275321622	SRC	ENSG00000197122	0.014167751832404	0.743
9606.ENSP00000355689	9606.ENSP00000362680	GUK1	ENSG00000143774	-0.590574395163386	SRC	ENSG00000197122	0.014167751832404	0.727
9606.ENSP00000262948	9606.ENSP00000362680	MAP2K2	ENSG00000126934	-0.294016138763207	SRC	ENSG00000197122	0.014167751832404	0.716
9606.ENSP00000078429	9606.ENSP00000362680	GNA11	ENSG00000088256	-0.640672184905867	SRC	ENSG00000197122	0.014167751832404	0.713

score = 0.7

Cellular response to estrogen stimulus

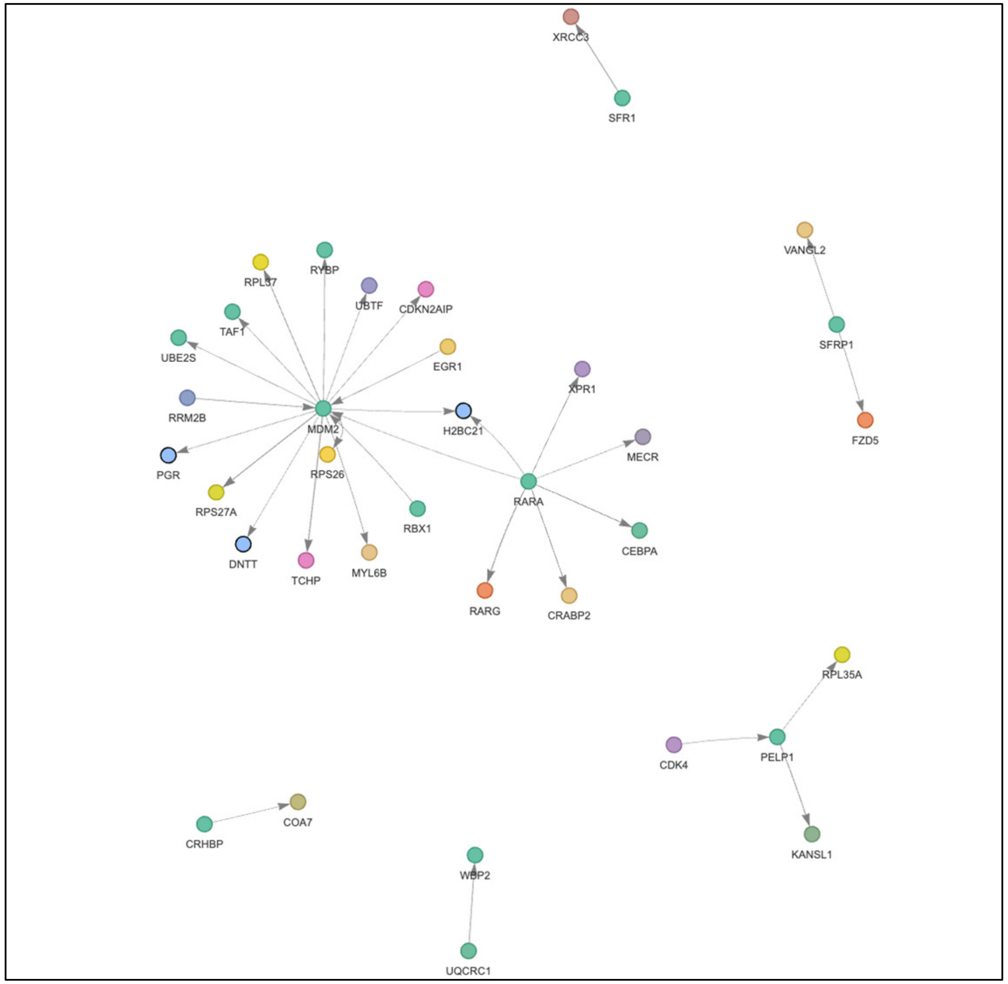

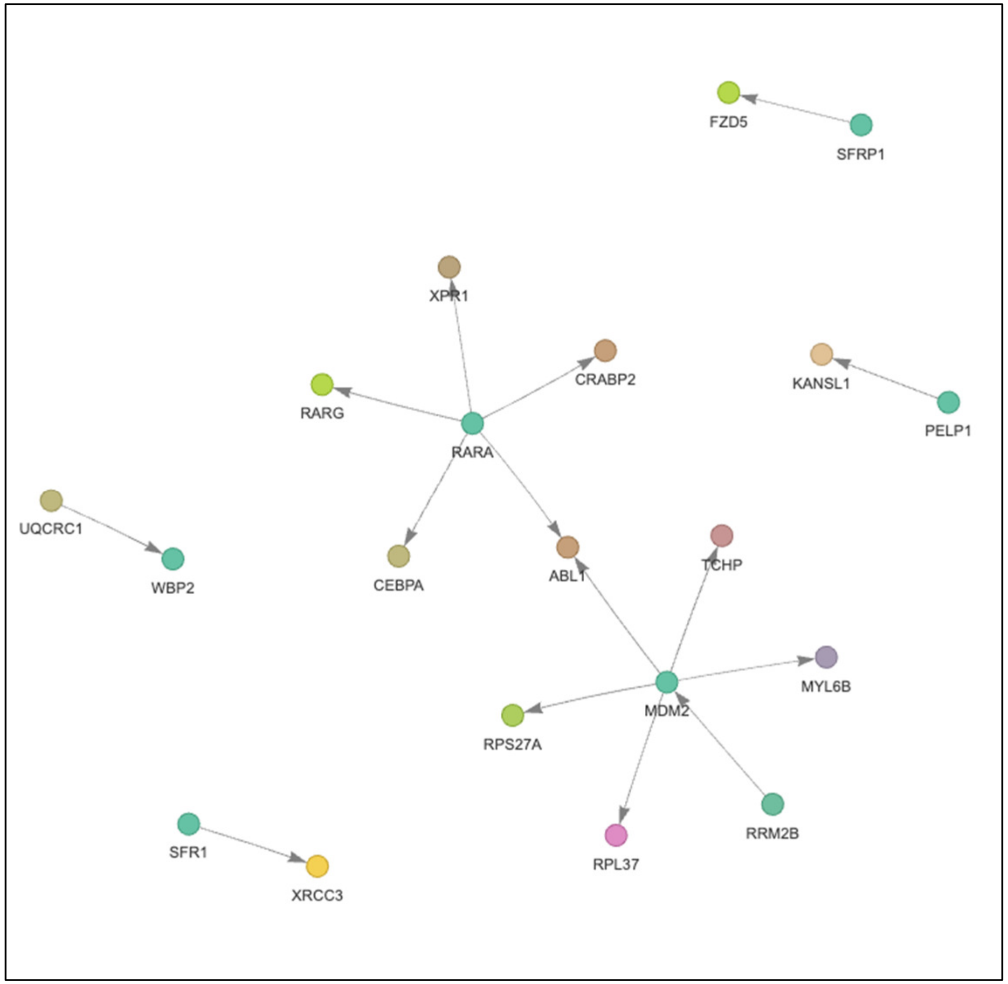

score = 0.4

score = 0.7

Cellular response to estrogen stimulus

stringId_A	stringId_B	preferredName_A	ensembl_gene_id_A	log2FoldChange_A	preferredName_B	ensembl_gene_id_B	log2FoldChange_B	score
**9606.ENSP00000258149**	9606.ENSP00000324404	MDM2	ENSG00000135679	0.673434120294442	TCHP	ENSG00000139437	0.134649870657416	0.997
**9606.ENSP00000258149**	9606.ENSP00000272317	MDM2	ENSG00000135679	0.673434120294442	RPS27A	ENSG00000143947	-0.0139888928125386	0.994
**9606.ENSP00000254066**	9606.ENSP00000388510	RARA	ENSG00000131759	-0.0631585338915923	RARG	ENSG00000172819	0.229870519121216	0.958
**9606.ENSP00000358742**	9606.ENSP00000451974	SFR1	ENSG00000156384	-2.44027600218733	XRCC3	ENSG00000126215	-0.0357158111932329	0.917
**9606.ENSP00000301396**	9606.ENSP00000387393	PELP1	ENSG00000141456	0.0592881496543431	KANSL1	ENSG00000120071	0.395114524749745	0.911
**9606.ENSP00000254066**	9606.ENSP00000427514	RARA	ENSG00000131759	-0.0631585338915923	CEBPA	ENSG00000245848	0.666116464877684	0.909
**9606.ENSP00000254066**	9606.ENSP00000482841	RARA	ENSG00000131759	-0.0631585338915923	CRABP2	ENSG00000143320	0.634236764442777	0.902
**9606.ENSP00000258149**	9606.ENSP00000274242	MDM2	ENSG00000135679	0.673434120294442	RPL37	ENSG00000145592	0.122922500541774	0.861
**9606.ENSP00000203407**	9606.ENSP00000467579	UQCRC1	ENSG00000010256	-0.0154577600448797	WBP2	ENSG00000132471	0.319052824015631	0.805
**9606.ENSP00000254066**	9606.ENSP00000356562	RARA	ENSG00000131759	-0.0631585338915923	XPR1	ENSG00000143324	0.0393972465402567	0.754
**9606.ENSP00000251810**	9606.ENSP00000258149	RRM2B	ENSG00000048392	-0.654764028813337	MDM2	ENSG00000135679	0.673434120294442	0.75
**9606.ENSP00000258149**	9606.ENSP00000450385	MDM2	ENSG00000135679	0.673434120294442	MYL6B	ENSG00000196465	-0.138701403716515	0.735
**9606.ENSP00000220772**	9606.ENSP00000354607	SFRP1	ENSG00000104332	-1.06181186373758	FZD5	ENSG00000163251	-1.9423149844083	0.728
**9606.ENSP00000258149**	9606.ENSP00000348849	MDM2	ENSG00000135679	0.673434120294442	RPS26	ENSG00000197728	0.314522683542367	0.691
**9606.ENSP00000258149**	9606.ENSP00000419494	MDM2	ENSG00000135679	0.673434120294442	RYBP	ENSG00000163602	0.274069549513414	0.685
**9606.ENSP00000239938**	9606.ENSP00000258149	EGR1	ENSG00000120738	0.799563342289828	MDM2	ENSG00000135679	0.673434120294442	0.684
**9606.ENSP00000220772**	9606.ENSP00000357040	SFRP1	ENSG00000104332	-1.06181186373758	VANGL2	ENSG00000162738	-0.0248485288694255	0.679
**9606.ENSP00000216225**	9606.ENSP00000258149	RBX1	ENSG00000100387	0.215494360361463	MDM2	ENSG00000135679	0.673434120294442	0.666
**9606.ENSP00000258149**	9606.ENSP00000406549	MDM2	ENSG00000135679	0.673434120294442	TAF1	ENSG00000147133	0.488578316634072	0.63
**9606.ENSP00000258149**	9606.ENSP00000264552	MDM2	ENSG00000135679	0.673434120294442	UBE2S	ENSG00000108106	0.5886535023913	0.622
**9606.ENSP00000254066**	9606.ENSP00000358151	RARA	ENSG00000131759	-0.0631585338915923	H2BC21	ENSG00000184678	NA	0.596
**9606.ENSP00000258149**	9606.ENSP00000325120	MDM2	ENSG00000135679	0.673434120294442	PGR	ENSG00000082175	NA	0.596
**9606.ENSP00000258149**	9606.ENSP00000302640	MDM2	ENSG00000135679	0.673434120294442	UBTF	ENSG00000108312	-0.184882671434266	0.588
**9606.ENSP00000258149**	9606.ENSP00000427108	MDM2	ENSG00000135679	0.673434120294442	CDKN2AIP	ENSG00000168564	-0.00932419063084441	0.574
**9606.ENSP00000274368**	9606.ENSP00000360593	CRHBP	ENSG00000145708	0.0128157665631034	COA7	ENSG00000162377	0.0416140603864908	0.572
**9606.ENSP00000254066**	9606.ENSP00000263702	RARA	ENSG00000131759	-0.0631585338915923	MECR	ENSG00000116353	-0.129437817377652	0.567
**9606.ENSP00000301396**	9606.ENSP00000495672	PELP1	ENSG00000141456	0.0592881496543431	RPL35A	ENSG00000182899	0.078889945641372	0.564
**9606.ENSP00000258149**	9606.ENSP00000358151	MDM2	ENSG00000135679	0.673434120294442	H2BC21	ENSG00000184678	NA	0.545
**9606.ENSP00000258149**	9606.ENSP00000360216	MDM2	ENSG00000135679	0.673434120294442	DNTT	ENSG00000107447	NA	0.528
**9606.ENSP00000257904**	9606.ENSP00000301396	CDK4	ENSG00000135446	1.09630707692628	PELP1	ENSG00000141456	0.0592881496543431	0.527
**9606.ENSP00000254066**	9606.ENSP00000258149	RARA	ENSG00000131759	-0.0631585338915923	MDM2	ENSG00000135679	0.673434120294442	0.526

score = 0.4

Protein (STRING preferred name)	Functional definition	Reference
**MDM2**	E3 ubiquitin ligase that targets p53 and other substrates; central regulator of stress-responsive transcriptional turnover and proteostasis.	Hamard PJ, Manfredi JJ. Mdm2's dilemma: to degrade or to translate p53?. Cancer Cell. 2012;21(1):3-5. doi:10.1016/j.ccr.2011.12.018
**RPS26**	Small-subunit ribosomal protein (40S) contributing to general translational capacity, here grouped as part of a “ribosomal component” module.	Belyy A, Levanova N, Tabakova I, Rospert S, Belyi Y. Ribosomal Protein Rps26 Influences 80S Ribosome Assembly in Saccharomyces cerevisiae. mSphere. 2016;1(1):e00109-15. Published 2016 Feb 24. doi:10.1128/mSphere.00109-15
**RPL37**	Large-subunit ribosomal protein (60S) contributing to protein synthesis, included as part of the translational/ribosomal cluster around MDM2.	Schwartz EI, Intine RV, Maraia RJ. CK2 is responsible for phosphorylation of human La protein serine-366 and can modulate rpL37 5'-terminal oligopyrimidine mRNA metabolism. Mol Cell Biol. 2004;24(21):9580-9591. doi:10.1128/MCB.24.21.9580-9591.2004
**RPS27A**	Ubiquitin–ribosomal protein fusion (ubiquitin–RPS27A) that couples ubiquitin supply with ribosome biogenesis; links ribosomal function to ubiquitin–proteasome pathways.	Eastham MJ, Pelava A, Wells GR, Watkins NJ, Schneider C. RPS27a and RPL40, Which Are Produced as Ubiquitin Fusion Proteins, Are Not Essential for p53 Signalling. Biomolecules. 2023;13(6):898. Published 2023 May 28. doi:10.3390/biom13060898
**UBE2S**	E2 ubiquitin-conjugating enzyme that elongates K11-linked ubiquitin chains for APC/C substrates, supporting proteasome-mediated protein turnover.	Wu T, Merbl Y, Huo Y, Gallop JL, Tzur A, Kirschner MW. UBE2S drives elongation of K11-linked ubiquitin chains by the anaphase-promoting complex. Proc Natl Acad Sci U S A. 2010;107(4):1355-1360. doi:10.1073/pnas.0912802107
**RBX1**	RING-box component of Cullin–RING ligases (SCF, VHL), essential activator of E3 ubiquitin ligase activity in ubiquitin–proteasome signalling.	Kamura T, Koepp DM, Conrad MN, et al. Rbx1, a component of the VHL tumor suppressor complex and SCF ubiquitin ligase. Science. 1999;284(5414):657-661. doi:10.1126/science.284.5414.657
**TAF1**	Largest and catalytically active subunit of TFIID (general transcription factor), integrating kinase and histone-acetyltransferase activities to regulate RNA Pol II transcription.	Bhattacharya S, Lou X, Hwang P, et al. Structural and functional insight into TAF1-TAF7, a subcomplex of transcription factor II D. Proc Natl Acad Sci U S A. 2014;111(25):9103-9108. doi:10.1073/pnas.1408293111
**UBTF**	Nucleolar transcription factor (UBF) that binds rDNA and activates RNA polymerase I transcription, linking chromatin state to ribosomal RNA synthesis.	Kuhn A, Stefanovsky V, Grummt I. The nucleolar transcription activator UBF relieves Ku antigen-mediated repression of mouse ribosomal gene transcription. Nucleic Acids Res. 1993;21(9):2057-2063. doi:10.1093/nar/21.9.2057
**RYBP**	Polycomb-associated repressor that interacts with PRC1 and transcription factor YY1, contributing to chromatin-based gene silencing.	García E, Marcos-Gutiérrez C, del Mar Lorente M, Moreno JC, Vidal M. RYBP, a new repressor protein that interacts with components of the mammalian Polycomb complex, and with the transcription factor YY1. EMBO J. 1999;18(12):3404-3418. doi:10.1093/emboj/18.12.3404
**EGR1**	Immediate-early zinc-finger transcription factor rapidly induced by stimuli; integrates stress and growth factor signals into transcriptional responses.	Duclot F, Kabbaj M. The Role of Early Growth Response 1 (EGR1) in Brain Plasticity and Neuropsychiatric Disorders. Front Behav Neurosci. 2017;11:35. Published 2017 Mar 6. doi:10.3389/fnbeh.2017.00035
**PGR**	Nuclear progesterone receptor (PR), a ligand-activated transcription factor mediating progesterone-responsive gene expression and cross-talk with other steroid receptors.	Li Z, Wei H, Li S, Wu P, Mao X. The Role of Progesterone Receptors in Breast Cancer. Drug Des Devel Ther. 2022;16:305-314. Published 2022 Jan 26. doi:10.2147/DDDT.S336643
**RARA**	Retinoic acid receptor alpha (RARα), nuclear receptor that heterodimerises with RXR and regulates retinoic-acid–responsive genes involved in development and differentiation.	Huang P, Chandra V, Rastinejad F. Retinoic acid actions through mammalian nuclear receptors. Chem Rev. 2014;114(1):233-254. doi:10.1021/cr400161b
**RARG**	Retinoic acid receptor gamma (RARγ), retinoid-regulated nuclear receptor contributing to developmental and morphogenetic retinoid signalling.	Kashyap V, Laursen KB, Brenet F, Viale AJ, Scandura JM, Gudas LJ. RARγ is essential for retinoic acid induced chromatin remodeling and transcriptional activation in embryonic stem cells. J Cell Sci. 2013;126(Pt 4):999-1008. doi:10.1242/jcs.119701
**CRABP2**	Cellular retinoic acid-binding protein II that binds all-trans retinoic acid in the cytosol and delivers it to nuclear RARs, modulating retinoid-responsive transcription.	Wolf G. Cellular retinoic acid-binding protein II: a coactivator of the transactivation by the retinoic acid receptor complex RAR.RXR. Nutr Rev. 2000;58(5):151-153. doi:10.1111/j.1753-4887.2000.tb01851.x
**CEBPA**	CCAAT/enhancer-binding protein alpha; bZIP transcription factor controlling adipogenesis, glucose and lipid metabolism, and myeloid differentiation.	Lourenço AR, Coffer PJ. A tumor suppressor role for C/EBPα in solid tumors: more than fat and blood. Oncogene. 2017;36(37):5221-5230. doi:10.1038/onc.2017.151
**XPR1**	Xenotropic and polytropic retrovirus receptor 1; multipass membrane protein that functions as a phosphate exporter and regulator of cellular phosphate homeostasis.	Chen L, He J, Wang M, She J. Structure and function of human XPR1 in phosphate export. Nat Commun. 2025;16(1):2983. Published 2025 Mar 26. doi:10.1038/s41467-025-58195-6
**MECR**	Mitochondrial trans-2-enoyl-CoA reductase; terminal enzyme of the mitochondrial fatty acid synthesis (mtFAS) pathway, supporting lipid and energy metabolism.	Steiner KK, Young AC, Patterson AR, et al. Mitochondrial fatty acid synthesis and MECR regulate CD4+ T cell function and oxidative metabolism. J Immunol. 2025;214(5):958-976. doi:10.1093/jimmun/vkaf034
**H2BC21**	Replication-independent histone H2B variant (H2B type 1-C/E/F/G/I) that contributes to nucleosomal structure and chromatin organisation.	NCBI Gene. H2BC21 – H2B clustered histone 21 [Homo sapiens]. Gene ID: 8349. Updated 2025. Accessed 01 December, 2025. https://www.ncbi.nlm.nih.gov/gene/8349
**PELP1**	Proline-, glutamic acid- and leucine-rich protein 1 (MNAR); nuclear receptor co-regulator and estrogen receptor coactivator integrating genomic and non-genomic ER signalling.	Manavathi B, Nair SS, Wang RA, Kumar R, Vadlamudi RK. Proline-, glutamic acid-, and leucine-rich protein-1 is essential in growth factor regulation of signal transducers and activators of transcription 3 activation. Cancer Res. 2005;65(13):5571-5577. doi:10.1158/0008-5472.CAN-04-4664
**KANSL1**	Non-catalytic subunit of the NSL histone-acetyltransferase complex that promotes histone H4 acetylation and transcriptional priming of developmental genes.	Dias J, Van Nguyen N, Georgiev P, et al. Structural analysis of the KANSL1/WDR5/KANSL2 complex reveals that WDR5 is required for efficient assembly and chromatin targeting of the NSL complex. Genes Dev. 2014;28(9):929-942. doi:10.1101/gad.240200.114
**RPL35A**	Large-subunit ribosomal protein (60S) that contributes to ribosome assembly and translational capacity.	Farrar JE, Nater M, Caywood E, et al. Abnormalities of the large ribosomal subunit protein, Rpl35a, in Diamond-Blackfan anemia. Blood. 2008;112(5):1582-1592. doi:10.1182/blood-2008-02-140012
**DNTT**	Terminal deoxynucleotidyl transferase (TdT); X-family DNA polymerase that adds non-templated nucleotides during V(D)J recombination in developing lymphocytes.	Motea EA, Berdis AJ. Terminal deoxynucleotidyl transferase: the story of a misguided DNA polymerase. Biochim Biophys Acta. 2010;1804(5):1151-1166. doi:10.1016/j.bbapap.2009.06.030
**SFRP1**	Secreted Frizzled-related protein 1; extracellular Wnt antagonist/modulator that binds Wnt ligands or Frizzled receptors and tunes Wnt signalling.	Elzi DJ, Song M, Hakala K, Weintraub ST, Shiio Y. Wnt antagonist SFRP1 functions as a secreted mediator of senescence. Mol Cell Biol. 2012;32(21):4388-4399. doi:10.1128/MCB.06023-11
**FZD5**	Frizzled-5, seven-pass Wnt receptor participating in canonical and non-canonical Wnt signalling and planar cell polarity pathways.	Range RC. Canonical and non-canonical Wnt signaling pathways define the expression domains of Frizzled 5/8 and Frizzled 1/2/7 along the early anterior-posterior axis in sea urchin embryos. Dev Biol. 2018;444(2):83-92. doi:10.1016/j.ydbio.2018.10.003
**VANGL2**	Core planar cell-polarity protein that transduces Wnt/PCP signals and controls cell polarity and morphogenetic movements.	Cheong SS, Akram KM, Matellan C, et al. The Planar Polarity Component VANGL2 Is a Key Regulator of Mechanosignaling. Front Cell Dev Biol. 2020;8:577201. Published 2020 Oct 29. doi:10.3389/fcell.2020.577201
**SFR1**	Swi5/Sfr1-like recombination factor; forms a complex with Rad51 paralogues to promote homologous recombination and genome stability.	Argunhan B, Murayama Y, Iwasaki H. The differentiated and conserved roles of Swi5-Sfr1 in homologous recombination. FEBS Lett. 2017;591(14):2035-2047. doi:10.1002/1873-3468.12656
**XRCC3**	RAD51-paralog DNA repair protein that forms a complex with RAD51C and participates in homologous recombination repair of double-strand breaks.	Pierce AJ, Johnson RD, Thompson LH, Jasin M. XRCC3 promotes homology-directed repair of DNA damage in mammalian cells. Genes Dev. 1999;13(20):2633-2638. doi:10.1101/gad.13.20.2633

Protein (STRING preferred name)	Functional definition	Reference
**SFRP1**	Secreted Wnt pathway antagonist; modulates Wnt–receptor interactions and can influence estrogen-regulated transcription via Wnt–ER crosstalk.	Elzi DJ, Song M, Hakala K, Weintraub ST, Shiio Y. Wnt antagonist SFRP1 functions as a secreted mediator of senescence. Mol Cell Biol. 2012;32(21):4388-4399. doi:10.1128/MCB.06023-11
**FZD5**	Frizzled family receptor binding Wnt ligands; participates in non-canonical Wnt signaling intersecting with steroid hormone responses.	Martinez-Marin D, Stroman GC, Fulton CJ, Pruitt K. Frizzled receptors: gatekeepers of Wnt signaling in development and disease. Front Cell Dev Biol. 2025;13:1599355. Published 2025 May 1. doi:10.3389/fcell.2025.1599355
**XRCC3**	DNA repair protein acting in homologous recombination; indirectly influences estrogen-responsive transcription via genome-stability control.	Brenneman MA, Wagener BM, Miller CA, Allen C, Nickoloff JA. XRCC3 controls the fidelity of homologous recombination: roles for XRCC3 in late stages of recombination. Mol Cell. 2002;10(2):387-395. doi:10.1016/s1097-2765(02)00595-6
**SFR1**	Component of the RAD51 recombination complex supporting homologous recombination; contributes to transcriptional responses requiring chromatin repair.	Akamatsu Y, Jasin M. Role for the mammalian Swi5-Sfr1 complex in DNA strand break repair through homologous recombination. PLoS Genet. 2010;6(10):e1001160. Published 2010 Oct 14. doi:10.1371/journal.pgen.1001160
**VANGL2**	Component of planar cell polarity pathway; contributes to cytoskeletal/adhesion regulation that interacts with hormone-sensitive signalling.	Cheong SS, Akram KM, Matellan C, et al. The Planar Polarity Component VANGL2 Is a Key Regulator of Mechanosignaling. Front Cell Dev Biol. 2020;8:577201. Published 2020 Oct 29. doi:10.3389/fcell.2020.577201
**WBP2**	Transcriptional co-activator for estrogen and progesterone receptors; enhances steroid-dependent gene expression.	Chen S, Wang H, Huang YF, et al. WW domain-binding protein 2: an adaptor protein closely linked to the development of breast cancer. Mol Cancer. 2017;16(1):128. Published 2017 Jul 19. doi:10.1186/s12943-017-0693-9
**UQCRC1**	Core subunit of mitochondrial complex III; supports oxidative phosphorylation relevant to energy-dependent hormone signalling.	Yi T, Chen H, Zhan J, et al. Ubiquinol-cytochrome c reductase core protein 1 contributes to cardiac tolerance to acute exhaustive exercise. Exp Biol Med (Maywood). 2022;247(2):165-173. doi:10.1177/15353702211046546
**COA7**	Mitochondrial assembly factor required for respiratory chain complex function; supports cellular energy environment affecting hormone-responsive pathways.	Formosa LE, Maghool S, Sharpe AJ, et al. Mitochondrial COA7 is a heme-binding protein with disulfide reductase activity, which acts in the early stages of complex IV assembly. Proc Natl Acad Sci U S A. 2022;119(9):e2110357119. doi:10.1073/pnas.2110357119

**System-level meaning**	**Proteins**
Changes in protein degradation, ribosomal stress and transcription	MDM2 (up), RPS26 (up), RPL37 (up), RPS27A (down), UBE2S (up), RBX1 (up), RRM2B (down), CDKN2AIP (down), TAF1 (up), UBTF (down), RYBP (up), EGR1 (up), MYL6B (down), TCHP (up), DNTT (NA), PGR (NA)
Altered retinoic-acid receptor pathways linked to estrogen response	RARA (down), RARG (up), CRABP2 (up), CEBPA (up), XPR1 (up), MECR (down), H2BC21 (NA)
Estrogen-linked control of G1/S transition and chromatin	PELP1 (up), CDK4 (up), KANSL1 (up), RPL35A (up)
Suppressed Wnt signalling affecting estrogen-responsive pathways	SFRP1 (down), FZD5 (down), VANGL2 (down)
Reduced homologous recombination capacity	SFR1 (down), XRCC3 (down)
Adjusted mitochondrial metabolism and hormone-binding proteins	WBP2 (up), UQCRC1 (down), COA7 (up), CRHBP (up)

Cellular response to estrogen stimulus

stringId_A	stringId_B	preferredName_A	ensembl_gene_id_A	log2FoldChange_A	preferredName_B	ensembl_gene_id_B	log2FoldChange_B	score
9606.ENSP00000258149	9606.ENSP00000324404	MDM2	ENSG00000135679	0.6734341202944428	TCHP	ENSG00000139437	0.1346498706574154	0.997
9606.ENSP00000258149	9606.ENSP00000272317	MDM2	ENSG00000135679	0.673434120294442	RPS27A	ENSG00000143947	-0.0139888928125386	0.994
9606.ENSP00000254066	9606.ENSP00000388510	RARA	ENSG00000131759	-0.0631585338915923	RARG	ENSG00000172819	0.229870519121216	0.958
9606.ENSP00000358742	9606.ENSP00000451974	SFR1	ENSG00000156384	-2.44027600218733	XRCC3	ENSG00000126215	-0.0357158111932329	0.917
9606.ENSP00000301396	9606.ENSP00000387393	PELP1	ENSG00000141456	0.0592881496543431	KANSL1	ENSG00000120071	0.395114524749745	0.911
9606.ENSP00000254066	9606.ENSP00000427514	RARA	ENSG00000131759	-0.0631585338915923	CEBPA	ENSG00000245848	0.666116464877684	0.909
9606.ENSP00000254066	9606.ENSP00000482841	RARA	ENSG00000131759	-0.0631585338915923	CRABP2	ENSG00000143320	0.634236764442777	0.902
9606.ENSP00000258149	9606.ENSP00000361423	MDM2	ENSG00000135679	0.673434120294442	ABL1	ENSG00000097007	0.8744892842113946	0.896
9606.ENSP00000258149	9606.ENSP00000274242	MDM2	ENSG00000135679	0.673434120294442	RPL37	ENSG00000145592	0.122922500541774	0.861
9606.ENSP00000203407	9606.ENSP00000467579	UQCRC1	ENSG00000010256	-0.0154577600448797	WBP2	ENSG00000132471	0.319052824015631	0.805
9606.ENSP00000254066	9606.ENSP00000356562	RARA	ENSG00000131759	-0.0631585338915923	XPR1	ENSG00000143324	0.0393972465402567	0.754
9606.ENSP00000251810	9606.ENSP00000258149	RRM2B	ENSG00000048392	-0.654764028813337	MDM2	ENSG00000135679	0.673434120294442	0.750
9606.ENSP00000258149	9606.ENSP00000450385	MDM2	ENSG00000135679	0.673434120294442	MYL6B	ENSG00000196465	-0.138701403716515	0.735
9606.ENSP00000220772	9606.ENSP00000354607	SFRP1	ENSG00000104332	-1.06181186373758	FZD5	ENSG00000163251	-1.9423149844083	0.728
9606.ENSP0000025406	9606.ENSP00000361423	RARA	ENSG00000131759	-0.0631585338915923	ABL1	ENSG00000097007	0.8744892842113946	0.719

score = 0.7

Androgen metabolic process

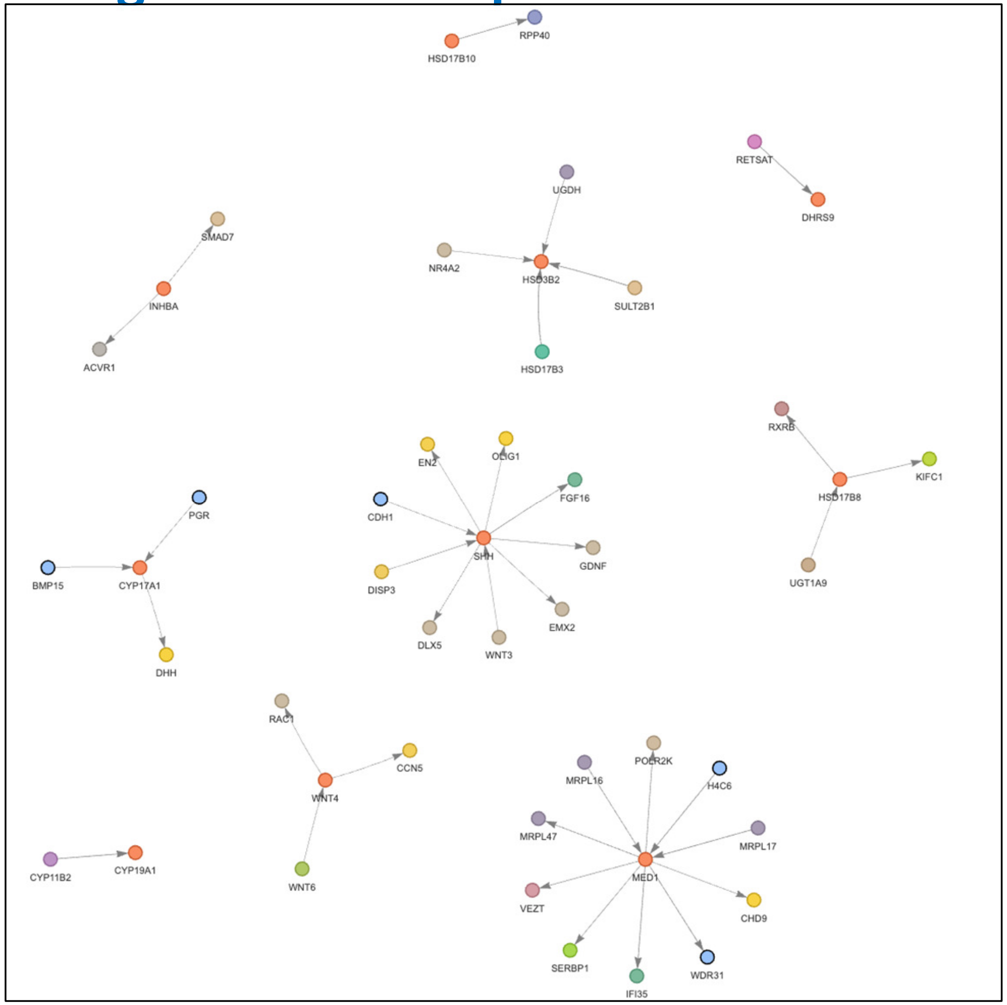

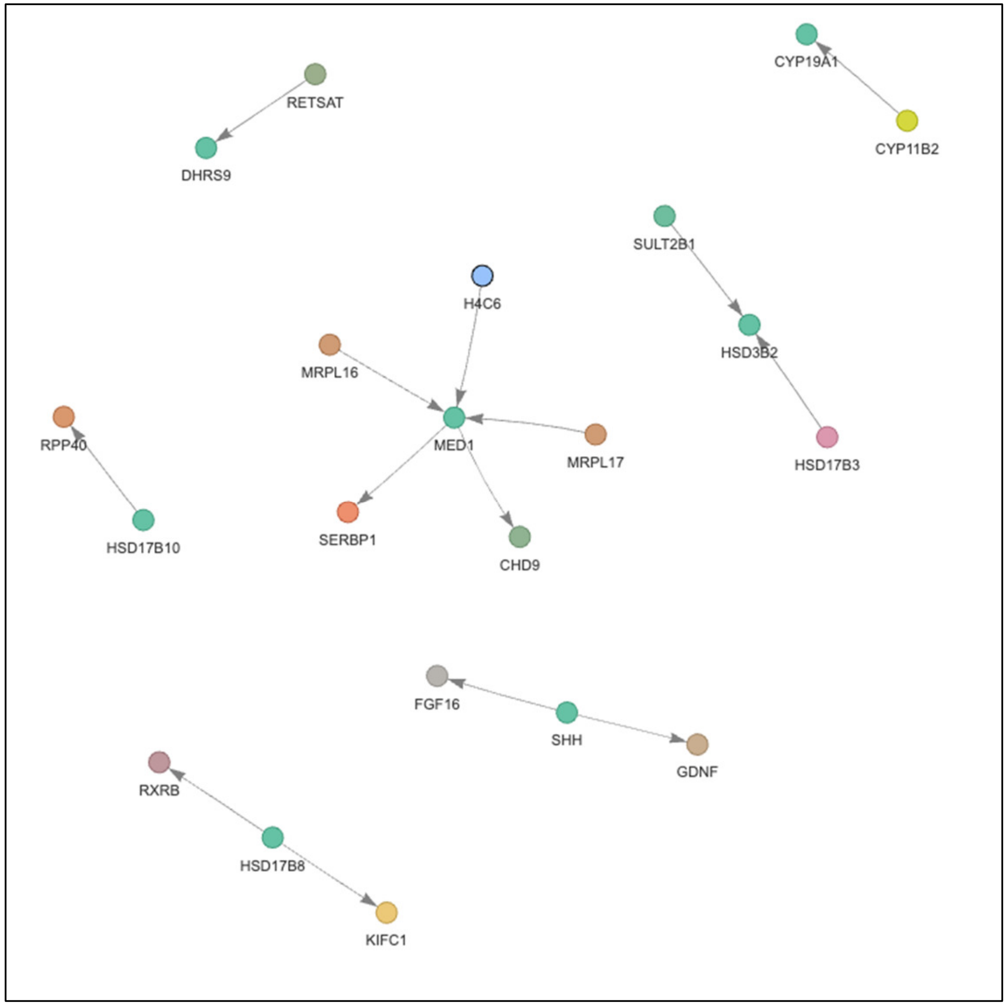

score = 0.4

score = 0.7

Androgen metabolic process

stringId_A	stringId_B	preferredName_A	ensembl_gene_id_A	log2FoldChange_A	preferredName_B	ensembl_gene_id_B	log2FoldChange_B	score
**9606.ENSP00000364412**	9606.ENSP00000445122	HSD17B3	ENSG00000130948	1.00017849812754	HSD3B2	ENSG00000203859	-0.833156486123551	0.987
**9606.ENSP00000288937**	9606.ENSP00000300651	MRPL17	ENSG00000158042	0.710576846930335	MED1	ENSG00000125686	-0.0198232731764219	0.983
**9606.ENSP00000325822**	9606.ENSP00000379683	CYP11B2	ENSG00000179142	0.228479383556454	CYP19A1	ENSG00000137869	-0.882362025384493	0.947
**9606.ENSP00000201586**	9606.ENSP00000445122	SULT2B1	ENSG00000088002	0.487528685865514	HSD3B2	ENSG00000203859	-0.833156486123551	0.942
**9606.ENSP00000295802**	9606.ENSP00000389241	RETSAT	ENSG00000042445	-0.402289898905001	DHRS9	ENSG00000073737	-0.309169165690439	0.917
**9606.ENSP00000300651**	9606.ENSP00000363308	MED1	ENSG00000125686	-0.0198232731764219	WDR31	ENSG00000148225	NA	0.916
**9606.ENSP00000300651**	9606.ENSP00000395590	MED1	ENSG00000125686	-0.0198232731764219	IFI35	ENSG00000068079	0.138029336432692	0.915
**9606.ENSP00000244537**	9606.ENSP00000300651	H4C6	ENSG00000274618	NA	MED1	ENSG00000125686	-0.0198232731764219	0.913
**9606.ENSP00000168216**	9606.ENSP00000369391	HSD17B10	ENSG00000072506	0.29147415617691	RPP40	ENSG00000124787	-0.0352293836860066	0.906
**9606.ENSP00000300651**	9606.ENSP00000417602	MED1	ENSG00000125686	-0.0198232731764219	MRPL47	ENSG00000136522	-0.115294678508557	0.848
**9606.ENSP00000300151**	9606.ENSP00000300651	MRPL16	ENSG00000166902	0.10409233244335	MED1	ENSG00000125686	-0.0198232731764219	0.83
**9606.ENSP00000363794**	9606.ENSP00000393963	HSD17B8	ENSG00000204228	-0.233688080890375	KIFC1	ENSG00000237649	-0.170773448631387	0.82
**9606.ENSP00000300651**	9606.ENSP00000342889	MED1	ENSG00000125686	-0.0198232731764219	POLR2K	ENSG00000147669	-0.02123600753193	0.814
**9606.ENSP00000297261**	9606.ENSP00000399324	SHH	ENSG00000164690	0.164875745572743	FGF16	ENSG00000196468	1.37667200784309	0.811
**9606.ENSP00000363794**	9606.ENSP00000363817	HSD17B8	ENSG00000204228	-0.233688080890375	RXRB	ENSG00000204231	0.0479562150646334	0.798
**9606.ENSP00000300651**	9606.ENSP00000410083	MED1	ENSG00000125686	-0.0198232731764219	VEZT	ENSG00000028203	-0.824755278959427	0.782
**9606.ENSP00000300651**	9606.ENSP00000360034	MED1	ENSG00000125686	-0.0198232731764219	SERBP1	ENSG00000142864	-0.195860136160896	0.761
**9606.ENSP00000297261**	9606.ENSP00000409007	SHH	ENSG00000164690	0.164875745572743	GDNF	ENSG00000168621	0.609500463075323	0.716
**9606.ENSP00000300651**	9606.ENSP00000396345	MED1	ENSG00000125686	-0.0198232731764219	CHD9	ENSG00000177200	-0.0710073592582278	0.702
**9606.ENSP00000319501**	9606.ENSP00000445122	UGDH	ENSG00000109814	0.191724334431065	HSD3B2	ENSG00000203859	-0.833156486123551	0.683
**9606.ENSP00000242208**	9606.ENSP00000405004	INHBA	ENSG00000122641	-0.276046478184335	ACVR1	ENSG00000115170	0.270392521608903	0.67
**9606.ENSP00000346768**	9606.ENSP00000363794	UGT1A9	ENSG00000241119	-0.607976524541623	HSD17B8	ENSG00000204228	-0.233688080890375	0.666
**9606.ENSP00000297261**	9606.ENSP00000498116	SHH	ENSG00000164690	0.164875745572743	DLX5	ENSG00000105880	-0.362919417259514	0.655
**9606.ENSP00000294484**	9606.ENSP00000297261	DISP3	ENSG00000204624	-1.71733386992125	SHH	ENSG00000164690	0.164875745572743	0.654
**9606.ENSP00000358903**	9606.ENSP00000497483	CYP17A1	ENSG00000148795	-0.988664869585963	DHH	ENSG00000139549	0.77316859545731	0.648
**9606.ENSP00000297261**	9606.ENSP00000450962	SHH	ENSG00000164690	0.164875745572743	EMX2	ENSG00000170370	-0.62887375913165	0.646
**9606.ENSP00000325120**	9606.ENSP00000358903	PGR	ENSG00000082175	NA	CYP17A1	ENSG00000148795	-0.988664869585963	0.605
**9606.ENSP00000290167**	9606.ENSP00000361959	WNT4	ENSG00000162552	0.642386637770552	CCN5	ENSG00000064205	0.52563273916771	0.6
**9606.ENSP00000297261**	9606.ENSP00000371785	SHH	ENSG00000164690	0.164875745572743	OLIG1	ENSG00000184221	0.387613177637705	0.599
**9606.ENSP00000261769**	9606.ENSP00000297261	CDH1	ENSG00000039068	NA	SHH	ENSG00000164690	0.164875745572743	0.59
**9606.ENSP00000242208**	9606.ENSP00000262158	INHBA	ENSG00000122641	-0.276046478184335	SMAD7	ENSG00000101665	-0.458315779319397	0.579
**9606.ENSP00000344479**	9606.ENSP00000445122	NR4A2	ENSG00000153234	0.77316859545731	HSD3B2	ENSG00000203859	-0.833156486123551	0.578
**9606.ENSP00000225512**	9606.ENSP00000297261	WNT3	ENSG00000108379	-1.60653356236968	SHH	ENSG00000164690	0.164875745572743	0.573
**9606.ENSP00000233948**	9606.ENSP00000290167	WNT6	ENSG00000115596	3.47971249904906	WNT4	ENSG00000162552	0.642386637770552	0.562
**9606.ENSP00000297261**	9606.ENSP00000297375	SHH	ENSG00000164690	0.164875745572743	EN2	ENSG00000164778	0.270622444142613	0.556
**9606.ENSP00000252677**	9606.ENSP00000358903	BMP15	ENSG00000130385	NA	CYP17A1	ENSG00000148795	-0.988664869585963	0.555
**9606.ENSP00000290167**	9606.ENSP00000348461	WNT4	ENSG00000162552	0.642386637770552	RAC1	ENSG00000136238	-0.119373003562648	0.555

score = 0.4

Protein	Functional definition	Reference
HSD17B3	Testis-expressed 17β-hydroxysteroid dehydrogenase that catalyses the final reduction of androstenedione to testosterone, defining the terminal step of classical androgen biosynthesis.	Planinic A, Maric T, Himelreich Peric M, Jezek D, Katusic Bojanac A. Dynamics of HSD17B3 expression in human fetal testis: implications for the role of Sertoli cells in fetal testosterone biosynthesis. Front Cell Dev Biol. 2024;12:1429292. Published 2024 Jul 30. doi:10.3389/fcell.2024.1429292
HSD3B2	3β-hydroxysteroid dehydrogenase/Δ5–Δ4 isomerase type 2 that converts pregnenolone and 17-hydroxypregnenolone to progesterone and 17-hydroxyprogesterone, providing precursors for downstream androgen and glucocorticoid synthesis.	Chen L, Huang H, Zhang H, Zhu G, Zhu M. Three cases of 3β-hydroxysteroid dehydrogenase deficiency: Clinical analysis. Adv Clin Exp Med. 2021;30(3):289-299. doi:10.17219/acem/131220
HSD17B10	Mitochondrial dehydrogenase involved in the oxidation of steroid substrates including neurosteroids and androgen derivatives.	He XY, Frackowiak J, Dobkin C, Brown WT, Yang SY. Involvement of Type 10 17β-Hydroxysteroid Dehydrogenase in the Pathogenesis of Infantile Neurodegeneration and Alzheimer's Disease. Int J Mol Sci. 2023;24(24):17604. Published 2023 Dec 18. doi:10.3390/ijms242417604
HSD17B8	Oxidizes active estrogens and androgens to less active forms.	Rotinen M, Celay J, Alonso MM, Arrazola A, Encio I, Villar J. Estradiol induces type 8 17beta-hydroxysteroid dehydrogenase expression: crosstalk between estrogen receptor alpha and C/EBPbeta. J Endocrinol. 2009;200(1):85-92. doi:10.1677/JOE-08-0134
CYP17A1	Microsomal cytochrome P450 with 17α-hydroxylase and 17,20-lyase activities that generates DHEA and androstenedione, key androgen precursors in adrenal and gonadal steroidogenesis.	Geller DH, Auchus RJ, Mendonça BB, Miller WL. The genetic and functional basis of isolated 17,20-lyase deficiency. Nat Genet. 1997;17(2):201-205. doi:10.1038/ng1097-201
CYP19A1	Aromatase; converts androgens to estrogens, rate-limiting step in estrogen biosynthesis.	Simpson ER, Davis SR. Minireview: aromatase and the regulation of estrogen biosynthesis--some new perspectives. Endocrinology. 2001 Nov;142(11):4589-94. doi: 10.1210/endo.142.11.8547. PMID: 11606422.
CYP11B2	Mitochondrial steroidogenic enzyme producing aldosterone; influences overall steroidogenic flux.	Reddish MJ, Guengerich FP. Human cytochrome P450 11B2 produces aldosterone by a processive mechanism due to the lactol form of the intermediate 18-hydroxycorticosterone. J Biol Chem. 2019;294(35):12975-12991. doi:10.1074/jbc.RA119.009830
WNT6	Secreted Wnt ligand strongly expressed in early embryonic and gonadal tissues; modulates cell fate and patterning and is associated with gonadal supporting-cell programs often linked to reduced androgenic tone.	Wei M, Zhang C, Tian Y, Du X, Wang Q, Zhao H. Expression and Function of WNT6: From Development to Disease. Front Cell Dev Biol. 2020;8:558155. Published 2020 Dec 9. doi:10.3389/fcell.2020.558155
WNT4	Canonical Wnt ligand essential for female gonadal development; suppresses Leydig cell differentiation and testicular androgen biosynthesis, thereby favouring ovarian/Müllerian trajectories.	Jeays-Ward K, Dandonneau M, Swain A. Wnt4 is required for proper male as well as female sexual development. Dev Biol. 2004;276(2):431-440. doi:10.1016/j.ydbio.2004.08.049
SHH	Sonic hedgehog morphogen that provides positional and patterning cues in multiple organs, including gonads, where Hedgehog signalling influences steroidogenic cell differentiation.	Komada M. Sonic hedgehog signaling coordinates the proliferation and differentiation of neural stem/progenitor cells by regulating cell cycle kinetics during development of the neocortex. Congenit Anom (Kyoto). 2012;52(2):72-77. doi:10.1111/j.1741-4520.2012.00368.x.
GDNF	Glial cell line–derived neurotrophic factor that is a key niche factor for spermatogonial stem cells, promoting their self-renewal and maintenance in the testis.	Hofmann MC. Gdnf signaling pathways within the mammalian spermatogonial stem cell niche. Mol Cell Endocrinol. 2008;288(1-2):95-103. doi:10.1016/j.mce.2008.04.012
FGF16	Paracrine fibroblast growth factor that supports embryonic heart development and myocardial growth; in this context considered as part of a broader growth-factor milieu linked to developmental responses to altered steroids.	Lu SY, Sheikh F, Sheppard PC, et al. FGF-16 is required for embryonic heart development. Biochem Biophys Res Commun. 2008;373(2):270-274. doi:10.1016/j.bbrc.2008.06.029
DLX5	Distal-less homeobox transcription factor involved in skeletal and craniofacial development and, in reproductive tissues, in Müllerian duct and other developmental programs sensitive to steroidal context.	Robledo RF et al. (2002). The Dlx5 and Dlx6 homeobox genes are essential for craniofacial, axial, and appendicular skeletal development. Genes Dev 16(9):1089–1101. PMID: 12000792.
EMX2	Homeobox transcription factor required for urogenital tract development; expressed in early primordia of kidneys, gonads and reproductive ducts, linking it to steroid-sensitive reproductive patterning.	Miyamoto N et al. (1997). Defects of urogenital development in mice lacking Emx2. Development 124(9):1653–1664. PMID: 9165114.
MED1	Core subunit of the Mediator complex and key nuclear receptor coactivator for androgen, estrogen, PPAR and other receptors, providing a scaffold that integrates steroid-dependent transcription.	Jin F, Claessens F, Fondell JD. Regulation of androgen receptor-dependent transcription by coactivator MED1 is mediated through a newly discovered noncanonical binding motif. J Biol Chem. 2012;287(2):858-870. doi:10.1074/jbc.M111.304519
IFI35	Interferon-induced protein 35 kDa (IFI35), an interferon-stimulated protein that modulates innate immune and inflammatory signalling; here considered as a stress/inflammation-related regulator within the steroid-responsive context.	De Masi R, Orlando S, Bagordo F, Grassi T. IFP35 Is a Relevant Factor in Innate Immunity, Multiple Sclerosis, and Other Chronic Inflammatory Diseases: A Review. Biology (Basel). 2021;10(12):1325. Published 2021 Dec 14. doi:10.3390/biology10121325
CHD9	Chromodomain helicase DNA-binding protein 9, an ATP-dependent chromatin remodeler that regulates gene expression programs and can modulate accessibility of nuclear receptor target genes.	Alendar A et al. (2020). Gene expression regulation by the chromodomain helicase DNA-binding protein 9 (CHD9) chromatin remodeler is dispensable for murine development. PLoS One 15(5): e0233394. PMID: 32453735.
MRPL47	Mitochondrial large-subunit ribosomal protein L47, component of the 39S mitoribosome, contributing to mitochondrial translation and thus to oxidative and steroidogenic metabolism.	NCBI Gene. MRPL47 mitochondrial ribosomal protein L47 (human), Gene ID: 57129. Updated 2025.
VEZT	Vezatin, an integral membrane protein of adherens junctions that links the cadherin–catenin complex to the actin cytoskeleton and participates in epithelial morphogenesis, including in preimplantation and other developmental contexts.	Küssel-Andermann P et al. (2000). Vezatin, a novel transmembrane protein, bridges myosin VIIA to the cadherin–catenins complex. EMBO J 19(22): 6020–6029. PMID: 11080149.
SERBP1	SERPINE1 mRNA-binding protein 1, an RNA-binding protein associated with ribosomes that regulates mRNA translation and cell-cycle related transcripts, contributing to post-transcriptional control in stress and signalling pathways.	Baudin A et al. (2021). Structural characterization of the RNA-binding protein SERBP1 reveals an RGG-motif-containing novel fold. Nucleic Acids Res 49(21): 12455–12470. PMID: 34631798.
POLR2K	Small shared subunit of RNA polymerases I, II and III, essential component of the basal transcription machinery that supports global mRNA synthesis, including of steroid-responsive genes.	NCBI Gene. POLR2K RNA polymerase II, I and III subunit K (human), Gene ID: 5440. Updated 2025.

Protein	Functional definition	Reference
**RETSAT**	Retinol saturase that converts all-trans-retinol to 13,14-dihydroretinol; regulates adipogenesis and lipid metabolism, linking vitamin A metabolism to cellular energy balance.	Schupp M, Lefterova MI, Janke J, *et al.* Retinol saturase promotes adipogenesis and is downregulated in obesity. *J Biol Chem.* 2009;284(28):19365-19371. doi:10.1074/jbc.M109.011403
**DHRS9**	Short-chain dehydrogenase/reductase functioning as a retinol and 3α-hydroxysteroid dehydrogenase; contributes to retinoic acid and steroid metabolism.	Belyaeva OV, Wirth SE, Boeglin WE, et al. Dehydrogenase reductase 9 (SDR9C4) and related homologs recognize a broad spectrum of lipid mediator oxylipins as substrates. J Biol Chem. 2022;298(1):101527. doi:10.1016/j.jbc.2021.101527
**RPP40**	Subunit of ribonuclease P/MRP complexes required for tRNA and rRNA processing, supporting global RNA maturation.	Esakova O, Krasilnikov AS. Of proteins and RNA: the RNase P/MRP family. RNA. 2010;16(9):1725-1747. doi:10.1261/rna.2214510
**MRPL16**	Mitochondrial ribosomal protein of the large (39S) subunit, required for mitochondrial translation and oxidative metabolism.	Koc EC, Haque ME, Spremulli LL. Current views of the structure of the mammalian mitochondrial ribosome. *Isr J Chem.* 2010;50(1):45-59.
**MRPL17**	Component of the 39S mitochondrial ribosomal large subunit, contributing to mitochondrial protein synthesis and energy production.	NCBI Gene: MRPL17 mitochondrial ribosomal protein L17 (human), Gene ID: 63875. Updated 2025.
**WDR31**	WD-repeat protein associated with primary cilia and centrosomes; implicated in cilium organization and signalling.	Cevik S, Peng X, Beyer T, et al. WDR31 displays functional redundancy with GTPase-activating proteins (GAPs) ELMOD and RP2 in regulating IFT complex and recruiting the BBSome to cilium. Life Sci Alliance. 2023;6(8):e202201844. Published 2023 May 19. doi:10.26508/lsa.202201844
**H4C6**	Replication-dependent histone H4 variant; core nucleosomal protein that packages DNA into chromatin and regulates transcriptional accessibility.	Talbert PB, Henikoff S. Histone variants at a glance. J Cell Sci. 2021;134(6):jcs244749. Published 2021 Mar 26. doi:10.1242/jcs.244749
**INHBA**	Encodes inhibin βA subunit; homodimers form activin A, a TGF-β family ligand regulating reproductive axis, folliculogenesis and gonadal development.	Welt C, Sidis Y, Keutmann H, Schneyer A. Activins, inhibins, and follistatins: from endocrinology to signaling. A paradigm for the new millennium. Exp Biol Med (Maywood). 2002;227(9):724-752. doi:10.1177/153537020222700905
**ACVR1**	Type I receptor serine/threonine kinase for BMP/activin ligands; transduces TGF-β family signals relevant to skeletal and reproductive development.	Wentworth KL, Lalonde RL, Groppe JC, et al. Functional Testing of Bone Morphogenetic Protein (BMP) Pathway Variants Identified on Whole-Exome Sequencing in a Patient with Delayed-Onset Fibrodysplasia Ossificans Progressiva (FOP) Using ACVR1R206H -Specific Human Cellular and Zebrafish Models. J Bone Miner Res. 2022;37(11):2058-2076. doi:10.1002/jbmr.4711
**SMAD7**	Inhibitory SMAD that negatively regulates TGF-β/BMP signalling by blocking receptor-mediated activation of receptor-regulated SMADs.	de Ceuninck van Capelle C, *et al.* Current perspectives on inhibitory SMAD7 in health and disease. *Crit Rev Biochem Mol Biol.* 2020;55(6):691-715. doi:10.1080/10409238.2020.1828260
**WNT3**	Canonical Wnt ligand essential for limb and craniofacial formation and urogenital development; activates β-catenin signalling.	Niemann S, Zhao C, Pascu F, et al. Homozygous WNT3 mutation causes tetra-amelia in a large consanguineous family. Am J Hum Genet. 2004;74(3):558-563. doi:10.1086/382196
**EN2**	Engrailed-2 homeobox transcription factor controlling midbrain/hindbrain and cerebellar patterning.	Cheng Y, Sudarov A, Szulc KU, et al. The Engrailed homeobox genes determine the different foliation patterns in the vermis and hemispheres of the mammalian cerebellum. Development. 2010;137(3):519-529. doi:10.1242/dev.027045
**OLIG1**	Basic helix–loop–helix transcription factor required for oligodendrocyte differentiation and CNS myelination.	Dai J, Bercury KK, Ahrendsen JT, Macklin WB. Olig1 function is required for oligodendrocyte differentiation in the mouse brain. J Neurosci. 2015;35(10):4386-4402. doi:10.1523/JNEUROSCI.4962-14.2015
**DISP3**	12-pass transmembrane “Dispatched” family protein; modulates Hedgehog-related and thyroid hormone-regulated signalling in neural tissues.	Zikova M, Corlett A, Bendova Z, Pajer P, Bartunek P. DISP3, a sterol-sensing domain-containing protein that links thyroid hormone action and cholesterol metabolism. Mol Endocrinol. 2009;23(4):520-528. doi:10.1210/me.2008-0271
**CDH1**	E-cadherin; calcium-dependent cell–cell adhesion molecule at epithelial adherens junctions, linking to the actin cytoskeleton.	Berx G, Van Roy F. The E-cadherin/catenin complex: an important gatekeeper in breast cancer tumorigenesis and malignant progression. Breast Cancer Res. 2001;3(5):289-293. doi:10.1186/bcr309
**RAC1**	Small Rho-family GTPase controlling actin cytoskeleton dynamics, cell migration and invasion.	Ma N, Xu E, Luo Q, Song G. Rac1: A Regulator of Cell Migration and a Potential Target for Cancer Therapy. Molecules. 2023;28(7):2976. Published 2023 Mar 27. doi:10.3390/molecules28072976
**NR4A2 (NURR1)**	Orphan nuclear receptor transcription factor involved in dopaminergic neuron development and broader metabolic and inflammatory programmes.	Luo GR, Chen Y, Li XP, Liu TX, Le WD. Nr4a2 is essential for the differentiation of dopaminergic neurons during zebrafish embryogenesis. Mol Cell Neurosci. 2008;39(2):202-210. doi:10.1016/j.mcn.2008.06.010
**UGDH**	UDP-glucose 6-dehydrogenase generating UDP-glucuronate, the precursor for glucuronidation reactions and glycosaminoglycan/hyaluronan synthesis; also androgen-regulated.	Price MJ, Nguyen AD, Byemerwa JK, Flowers J, Baëta CD, Goodwin CR. UDP-glucose dehydrogenase (UGDH) in clinical oncology and cancer biology. Oncotarget. 2023;14:843-857. Published 2023 Sep 28. doi:10.18632/oncotarget.28514

System-level meaning	Proteins
Altered developmental morphogen signalling intersecting with androgen pathways	SHH (up), WNT6 (up), WNT4 (up), WNT3 (down), FGF16 (up), GDNF (up), DLX5 (down), EMX2 (down), EN2 (up), OLIG1 (up), DISP3 (down), CDH1 (NA), RAC1 (down)
Disrupted precursor androgen synthesis with compensatory activation of later steps	HSD3B2 (down), HSD17B3 (up), NR4A2 (up), SULT2B1 (up), UGDH (up)
Modified mitochondrial steroid oxidation and RNA-processing link	HSD17B10 (up), RPP40 (down)
Reduced retinoid/steroid oxidation capacity	DHRS9 (down), RETSAT (down)
Shift in androgen/estrogen inactivation and intracellular transport	HSD17B8 (down), RXRB (up), KIFC1 (down), UGT1A9 (down)
Weakened activin/TGFβ regulation influencing steroidogenesis	INHBA (down), ACVR1 (up), SMAD7 (down)
Altered steroidogenic enzyme regulation and gonadal signalling cues	CYP17A1 (down), DHH (up), PGR (NA), BMP15 (NA), CYP11B2 (up), CYP19A1 (down)
Rewiring of transcriptional co-activation and mitochondrial translation networks	MED1 (unchanged), MRPL16 (up), MRPL17 (up), MRPL47 (down), POLR2K (down), SERBP1 (down), VEZT (down), IFI35 (up), WDR31 (NA), H4C6 (NA)

Androgen metabolic process

stringId_A	stringId_B	preferredName_A	ensembl_gene_id_A	log2FoldChange_A	preferredName_B	ensembl_gene_id_B	log2FoldChange_B	score
9606.ENSP00000364412	9606.ENSP00000445122	HSD17B3	ENSG00000130948	1.00017849812754	HSD3B2	ENSG00000203859	-0.833156486123551	0.987
9606.ENSP00000288937	9606.ENSP00000300651	MRPL17	ENSG00000158042	0.710576846930335	MED1	ENSG00000125686	-0.0198232731764219	0.983
9606.ENSP00000325822	9606.ENSP00000379683	CYP11B2	ENSG00000179142	0.228479383556454	CYP19A1	ENSG00000137869	-0.882362025384493	0.947
9606.ENSP00000201586	9606.ENSP00000445122	SULT2B1	ENSG00000088002	0.487528685865514	HSD3B2	ENSG00000203859	-0.833156486123551	0.942
9606.ENSP00000295802	9606.ENSP00000389241	RETSAT	ENSG00000042445	-0.402289898905001	DHRS9	ENSG00000073737	-0.309169165690439	0.917
9606.ENSP00000244537	9606.ENSP00000300651	H4C6	ENSG00000274618	NA	MED1	ENSG00000125686	-0.0198232731764219	0.913
9606.ENSP00000168216	9606.ENSP00000369391	HSD17B10	ENSG00000072506	0.29147415617691	RPP40	ENSG00000124787	-0.0352293836860066	0.906
9606.ENSP00000300151	9606.ENSP00000300651	MRPL16	ENSG00000166902	0.10409233244335	MED1	ENSG00000125686	-0.0198232731764219	0.83
9606.ENSP00000363794	9606.ENSP00000393963	HSD17B8	ENSG00000204228	-0.233688080890375	KIFC1	ENSG00000237649	-0.170773448631387	0.82
9606.ENSP00000297261	9606.ENSP00000399324	SHH	ENSG00000164690	0.164875745572743	FGF16	ENSG00000196468	1.37667200784309	0.811
9606.ENSP00000363794	9606.ENSP00000363817	HSD17B8	ENSG00000204228	-0.233688080890375	RXRB	ENSG00000204231	0.0479562150646334	0.798
9606.ENSP00000300651	9606.ENSP00000360034	MED1	ENSG00000125686	-0.0198232731764219	SERBP1	ENSG00000142864	-0.195860136160896	0.761
9606.ENSP00000297261	9606.ENSP00000409007	SHH	ENSG00000164690	0.164875745572743	GDNF	ENSG00000168621	0.609500463075323	0.716
9606.ENSP00000300651	9606.ENSP00000396345	MED1	ENSG00000125686	-0.0198232731764219	CHD9	ENSG00000177200	-0.0710073592582278	0.702

score = 0.7

Androgen biosynthetic process

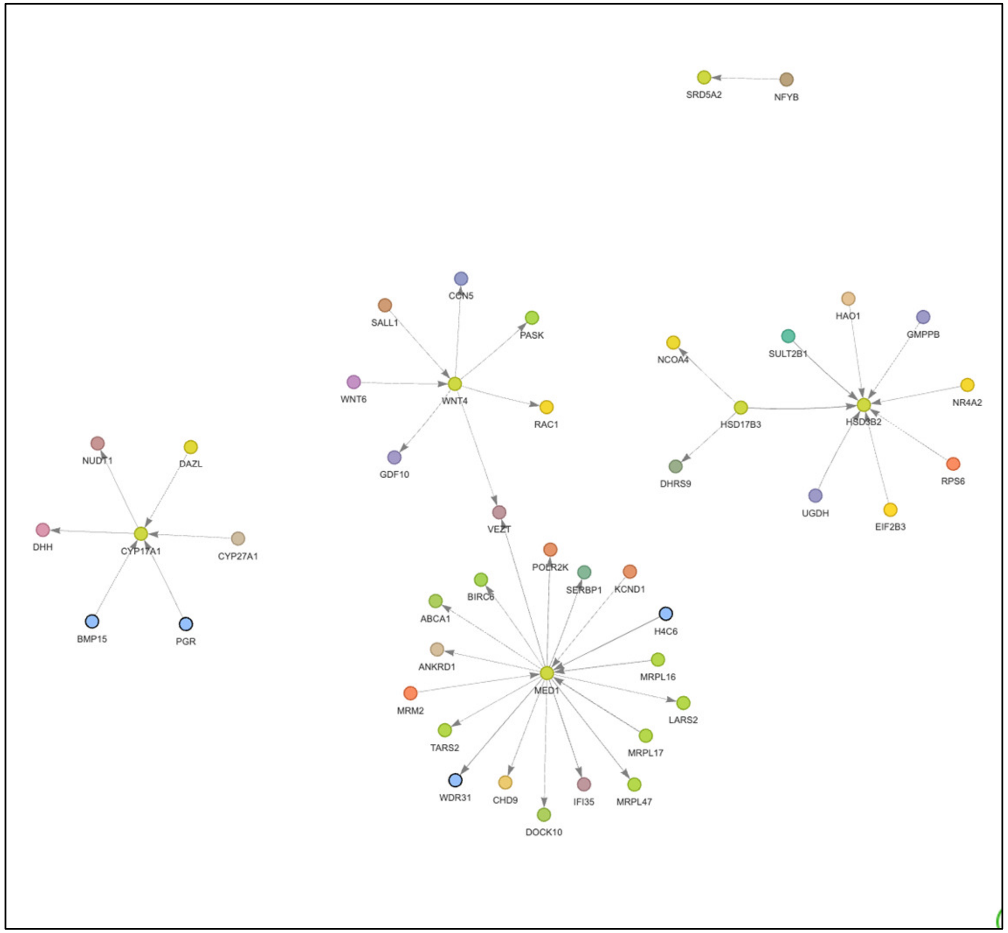

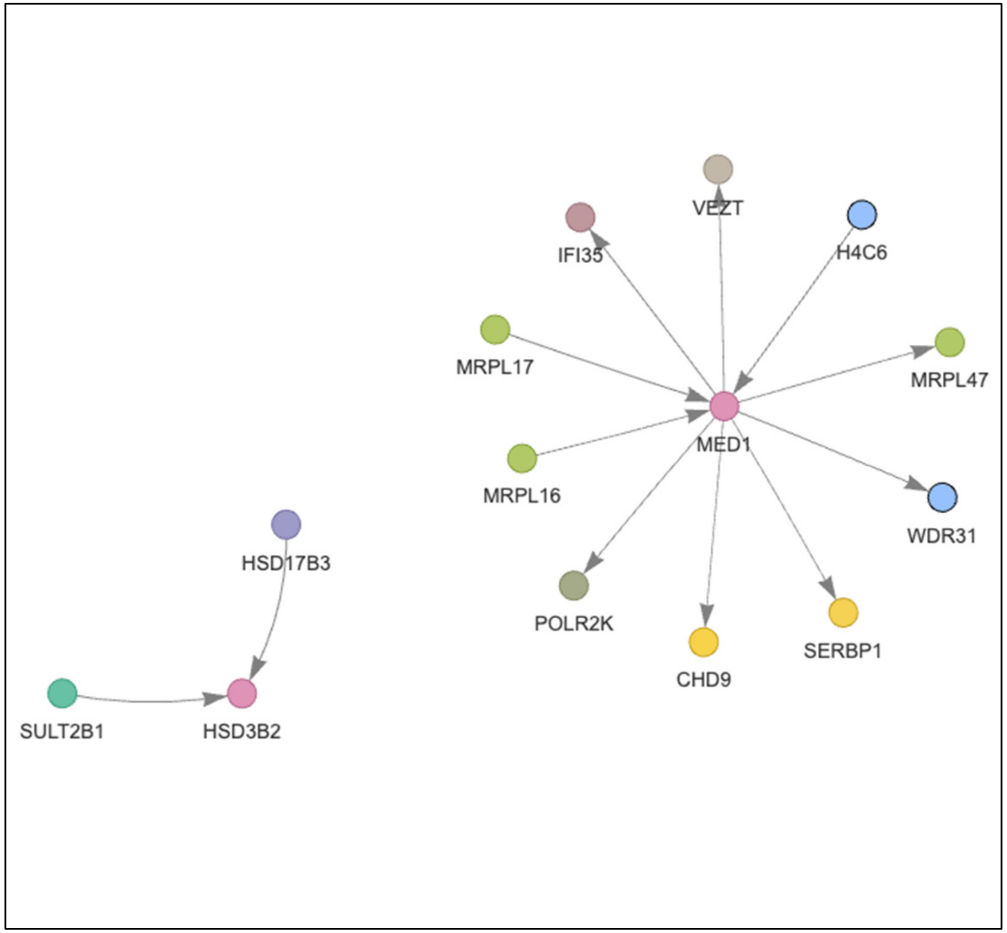

score = 0.4

score = 0.7

Androgen biosynthetic process

stringId_A	stringId_B	preferredName_A	ensembl_gene_id_A	log2FoldChange_A	preferredName_B	ensembl_gene_id_B	log2FoldChange_B	score
**9606.ENSP00000201586**	9606.ENSP00000445122	SULT2B1	ENSG00000088002	0.487528685865514	HSD3B2	ENSG00000203859	-0.833156486123551	0.942
**9606.ENSP00000309092**	9606.ENSP00000445122	GMPPB	ENSG00000173540	-0.0120969351177152	HSD3B2	ENSG00000203859	-0.833156486123551	0.512
**9606.ENSP00000319501**	9606.ENSP00000445122	UGDH	ENSG00000109814	0.191724334431065	HSD3B2	ENSG00000203859	-0.833156486123551	0.683
**9606.ENSP00000344479**	9606.ENSP00000445122	NR4A2	ENSG00000153234	0.77316859545731	HSD3B2	ENSG00000203859	-0.833156486123551	0.578
**9606.ENSP00000353575**	9606.ENSP00000445122	EIF2B3	ENSG00000070785	-0.36653265714018	HSD3B2	ENSG00000203859	-0.833156486123551	0.512
**9606.ENSP00000364412**	9606.ENSP00000463027	HSD17B3	ENSG00000130948	1.00017849812754	NCOA4	ENSG00000266412	-0.242683991495028	0.416
**9606.ENSP00000364412**	9606.ENSP00000389241	HSD17B3	ENSG00000130948	1.00017849812754	DHRS9	ENSG00000073737	-0.309169165690439	0.49
**9606.ENSP00000364412**	9606.ENSP00000445122	HSD17B3	ENSG00000130948	1.00017849812754	HSD3B2	ENSG00000203859	-0.833156486123551	0.987
**9606.ENSP00000368066**	9606.ENSP00000445122	HAO1	ENSG00000101323	-0.322379815548293	HSD3B2	ENSG00000203859	-0.833156486123551	0.485
**9606.ENSP00000369757**	9606.ENSP00000445122	RPS6	ENSG00000137154	-0.371972791678673	HSD3B2	ENSG00000203859	-0.833156486123551	0.531
**9606.ENSP00000250863**	9606.ENSP00000358903	DAZL	ENSG00000092345	-1.20142421383256	CYP17A1	ENSG00000148795	-0.988664869585963	0.4
**9606.ENSP00000252677**	9606.ENSP00000358903	BMP15	ENSG00000130385	NA	CYP17A1	ENSG00000148795	-0.988664869585963	0.555
**9606.ENSP00000258415**	9606.ENSP00000358903	CYP27A1	ENSG00000135929	0.601320195091909	CYP17A1	ENSG00000148795	-0.988664869585963	0.411
**9606.ENSP00000325120**	9606.ENSP00000358903	PGR	ENSG00000082175	NA	CYP17A1	ENSG00000148795	-0.988664869585963	0.605
**9606.ENSP00000358903**	9606.ENSP00000380241	CYP17A1	ENSG00000148795	-0.988664869585963	NUDT1	ENSG00000106268	0.0128353075424751	0.456
**9606.ENSP00000358903**	9606.ENSP00000497483	CYP17A1	ENSG00000148795	-0.988664869585963	DHH	ENSG00000139549	0.77316859545731	0.648
**9606.ENSP00000240055**	9606.ENSP00000477587	NFYB	ENSG00000120837	-0.0788484718978411	SRD5A2	ENSG00000277893	-0.358743675036838	0.54
**9606.ENSP00000218176**	9606.ENSP00000300651	KCND1	ENSG00000102057	-0.535609338780258	MED1	ENSG00000125686	-0.0198232731764219	0.522
**9606.ENSP00000233948**	9606.ENSP00000290167	WNT6	ENSG00000115596	3.47971249904906	WNT4	ENSG00000162552	0.642386637770552	0.562
**9606.ENSP00000242257**	9606.ENSP00000300651	MRM2	ENSG00000122687	-0.262021962677258	MED1	ENSG00000125686	-0.0198232731764219	0.427
**9606.ENSP00000244537**	9606.ENSP00000300651	H4C6	ENSG00000274618	NA	MED1	ENSG00000125686	-0.0198232731764219	0.913
**9606.ENSP00000251020**	9606.ENSP00000290167	SALL1	ENSG00000103449	0.451909008058015	WNT4	ENSG00000162552	0.642386637770552	0.457
**9606.ENSP00000288937**	9606.ENSP00000300651	MRPL17	ENSG00000158042	0.710576846930335	MED1	ENSG00000125686	-0.0198232731764219	0.983
**9606.ENSP00000290167**	9606.ENSP00000351475	WNT4	ENSG00000162552	0.642386637770552	PASK	ENSG00000115687	0.227022044537624	0.4
**9606.ENSP00000290167**	9606.ENSP00000410083	WNT4	ENSG00000162552	0.642386637770552	VEZT	ENSG00000028203	-0.824755278959427	0.431
**9606.ENSP00000290167**	9606.ENSP00000464145	WNT4	ENSG00000162552	0.642386637770552	GDF10	ENSG00000266524	-0.53064835333416	0.485
**9606.ENSP00000290167**	9606.ENSP00000348461	WNT4	ENSG00000162552	0.642386637770552	RAC1	ENSG00000136238	-0.119373003562648	0.555
**9606.ENSP00000290167**	9606.ENSP00000361959	WNT4	ENSG00000162552	0.642386637770552	CCN5	ENSG00000064205	0.52563273916771	0.6
**9606.ENSP00000300151**	9606.ENSP00000300651	MRPL16	ENSG00000166902	0.10409233244335	MED1	ENSG00000125686	-0.0198232731764219	0.83
**9606.ENSP00000300651**	9606.ENSP00000498867	MED1	ENSG00000125686	-0.0198232731764219	LARS2	ENSG00000011376	-0.953190102551873	0.427
**9606.ENSP00000300651**	9606.ENSP00000360762	MED1	ENSG00000125686	-0.0198232731764219	ANKRD1	ENSG00000148677	-0.0710234065399214	0.433
**9606.ENSP00000300651**	9606.ENSP00000358060	MED1	ENSG00000125686	-0.0198232731764219	TARS2	ENSG00000143374	0.56835349935444	0.44
**9606.ENSP00000300651**	9606.ENSP00000493664	MED1	ENSG00000125686	-0.0198232731764219	DOCK10	ENSG00000135905	-0.0465332227748506	0.475
**9606.ENSP00000300651**	9606.ENSP00000363868	MED1	ENSG00000125686	-0.0198232731764219	ABCA1	ENSG00000165029	0.00334098435421702	0.496
**9606.ENSP00000300651**	9606.ENSP00000393596	MED1	ENSG00000125686	-0.0198232731764219	BIRC6	ENSG00000115760	0.506126248763016	0.498
**9606.ENSP00000300651**	9606.ENSP00000396345	MED1	ENSG00000125686	-0.0198232731764219	CHD9	ENSG00000177200	-0.0710073592582278	0.702
**9606.ENSP00000300651**	9606.ENSP00000360034	MED1	ENSG00000125686	-0.0198232731764219	SERBP1	ENSG00000142864	-0.195860136160896	0.761
**9606.ENSP00000300651**	9606.ENSP00000410083	MED1	ENSG00000125686	-0.0198232731764219	VEZT	ENSG00000028203	-0.824755278959427	0.782
**9606.ENSP00000300651**	9606.ENSP00000342889	MED1	ENSG00000125686	-0.0198232731764219	POLR2K	ENSG00000147669	-0.02123600753193	0.814
**9606.ENSP00000300651**	9606.ENSP00000417602	MED1	ENSG00000125686	-0.0198232731764219	MRPL47	ENSG00000136522	-0.115294678508557	0.848
**9606.ENSP00000300651**	9606.ENSP00000395590	MED1	ENSG00000125686	-0.0198232731764219	IFI35	ENSG00000068079	0.138029336432692	0.915
**9606.ENSP00000300651**	9606.ENSP00000363308	MED1	ENSG00000125686	-0.0198232731764219	WDR31	ENSG00000148225	NA	0.916

score = 0.4

Protein	Functional definition	Reference
HSD3B2	3β-hydroxysteroid dehydrogenase/Δ5–Δ4 isomerase type 2 that converts pregnenolone and 17-hydroxypregnenolone to progesterone and 17-hydroxyprogesterone, providing precursors for downstream androgen and glucocorticoid synthesis.	Chen L, Huang H, Zhang H, Zhu G, Zhu M. Three cases of 3β-hydroxysteroid dehydrogenase deficiency: Clinical analysis. Adv Clin Exp Med. 2021;30(3):28 299. doi:10.17219/acem/131220
CYP17A1	Microsomal cytochrome P450 with 17α-hydroxylase and 17,20-lyase activities that generates DHEA and androstenedione, key androgen precursors in adrenal and gonadal steroidogenesis.	Geller DH, Auchus RJ, Mendonça BB, Miller WL. The genetic and functional basis of isolated 17,20-lyase deficiency. Nat Genet. 1997;17(2):201-205. doi:10.1038/ng1097-201
HSD17B3	Testis-expressed 17β-hydroxysteroid dehydrogenase that catalyses the final reduction of androstenedione to testosterone, defining the terminal step of classical androgen biosynthesis.	Planinic A, Maric T, Himelreich Peric M, Jezek D, Katusic Bojanac A. Dynamics of HSD17B3 expression in human fetal testis: implications for the role of Ser cells in fetal testosterone biosynthesis. Front Cell Dev Biol. 2024;12:1429292. Published 2024 Jul 30. doi:10.3389/fcell.2024.1429292
SULT2B1	Steroid sulfotransferase adding sulfate groups to hydroxysteroids, regulating androgen availability and inactivation.	Fuda H, et al. “Characterization and tissue distribution of human SULT2B1b, a cholesterol sulfotransferase.” J Biol Chem. 2002;277: 36161–36168. PMI 12138160
NR4A2	Orphan nuclear receptor regulating transcriptional programs and modulating steroidogenic pathways via co-regulator interactions.	Maxwell MA & Muscat GEO. “The NR4A subgroup: immediate early response genes with pleiotropic physiological roles.” Nucl Recept Signal. 2006;4:e00 PMID: 16604165
UGDH	UDP-glucose dehydrogenase generating UDP-glucuronate, required for glucuronidation of steroids and metabolites.	Ouyang Y, et al. “UDP-glucose dehydrogenase: structure and function.” Crit Rev Biochem Mol Biol. 2008;43(1):1–16. PMID: 18172683
HAO1	Glycolate oxidase involved in peroxisomal metabolism; supports cellular redox state and intermediary metabolism affecting steroidogenesis.	Rumsby G, et al. “Primary hyperoxaluria type 3 caused by mutations in HAO1.” N Engl J Med. 2010;363(21):1982–1990. PMID: 21083381
RPS6	Ribosomal protein S6; regulates translation efficiency and mTOR-linked signaling pathways responsive to growth and hormone signals.	Ruvinsky I & Meyuhas O. “Ribosomal protein S6 phosphorylation: from protein synthesis to cell size.” Trends Biochem Sci. 2006;31(6):342–348. PMID: 16714167
EIF2B3	Subunit of eIF2B regulating initiation of translation under stress and hormone-dependent conditions.	van der Knaap MS, et al. “Mutations in eIF2B3 cause leukoencephalopathy with vanishing white matter.” Nat Genet. 2002;29(4):383–388. PMID: 118101
DAZL	Germ-cell–specific RNA-binding protein essential for male germ-cell development; indirectly linked to androgen-dependent spermatogenesis.	Ruggiu M, et al. “The RNA-binding protein DAZL is essential for gametogenesis.” Nature. 2000;403: 486–489. PMID: 10676954
DHH	Sertoli-cell–derived Hedgehog ligand crucial for fetal Leydig cell differentiation and testicular development.	Bitgood MJ, Shen L, McMahon AP. “Sertoli cells are the primary source of Desert hedgehog in the developing mouse testis.” Development. 1996;122(10): 3 3165. PMID: 8898223
SRD5A2	Steroid 5α-reductase type 2 converting testosterone → dihydrotestosterone (DHT); key enzyme in androgen activation.	Imperato-McGinley J & Zhu YS. “5α-reductase type 2 deficiency.” Endocrinol Metab Clin North Am. 1999;28(2): 341–356. PMID: 10384820
NFYB	Subunit of NF-Y transcription factor, modulating promoter activity of metabolic and hormonal genes, including steroidogenic pathways.	Dolfini D, et al. “The CCAAT box-binding NF-Y complex: a key regulator in transcription.” Gene. 2012;518(1): 1–7. PMID: 22305978
MED1	Core subunit of the Mediator complex and key nuclear receptor coactivator for androgen, estrogen, PPAR and other receptors, providing a scaffold that integrates steroid-dependent transcription.	Jin F, Claessens F, Fondell JD. Regulation of androgen receptor-dependent transcription by coactivator MED1 is mediated through a newly discovered noncanonical binding motif. J Biol Chem. 2012;287(2):858-870. doi:10.1074/jbc.M111.304519
MRPL16 / MRPL17 / MRPL47	Mitochondrial ribosomal proteins required for mitochondrial translation, supporting energy metabolism needed for steroid biosynthesis.	NCBI Gene. MRPL16 mitochondrial ribosomal protein L16 (human), Gene ID: 54948. Updated 2025.<br>MRPL17 mitochondrial ribosomal protein L17 [Homo sapiens (human)]<br>Gene ID: 63875, updated on 25-Nov-2025<br>NCBI Gene. MRPL47 mitochondrial ribosomal protein L47 (human), Gene ID: 57129. Updated 2025.
LARS2 / TARS2	Aminoacyl-tRNA synthetases essential for mitochondrial protein synthesis and oxidative function affecting steroidogenic capacity.	Konovalova S & Tyynismaa H. “Mitochondrial aminoacyl-tRNA synthetases in human disease.” Mol Genet Metab. 2013;108(4): 206–211. PMID: 2353868
ABCA1	Cholesterol efflux transporter controlling intracellular sterol levels; impacts steroidogenic substrate availability.	Oram JF & Vaughan AM. “ATP-binding cassette cholesterol transporters and cardiovascular disease.” Circ Res. 2006;99(10):1031–1043. PMID: 1709573
CHD9	Chromodomain helicase DNA-binding protein 9, an ATP-dependent chromatin remodeler that regulates gene expression programs and can modulate accessibility of nuclear receptor target genes.	Alendar A et al. (2020). Gene expression regulation by the chromodomain helicase DNA-binding protein 9 (CHD9) chromatin remodeler is dispensable fo murine development. PLoS One 15(5): e0233394. PMID: 32453735.
SERBP1	SERPINE1 mRNA-binding protein 1, an RNA-binding protein associated with ribosomes that regulates mRNA translation and cell-cycle related transcripts, contributing to post-transcriptional control in stress and signalling pathways.	Baudin A et al. (2021). Structural characterization of the RNA-binding protein SERBP1 reveals an RGG-motif-containing novel fold. Nucleic Acids Res 49(2 12455–12470. PMID: 34631798.
BIRC6	Anti-apoptotic protein (IAP family) functioning as E2/E3 hybrid ubiquitin ligase; modulates stress responses.	Bartke T & Pohl C. “The ubiquitin system in programmed cell death.” Nat Rev Mol Cell Biol. 2020;21: 653–668. PMID: 32958835
DOCK10	Guanine nucleotide exchange factor involved in actin regulation and cell signaling.	Gadea G & Blangy A. “Dock-family exchange factors in cell migration and morphogenesis.” Eur J Cell Biol. 2014;93(10–12): 466–477. PMID: 25468070
POLR2K	Small shared subunit of RNA polymerases I, II and III, essential component of the basal transcription machinery that supports global mRNA synthesis, including of steroid-responsive genes.	NCBI Gene. POLR2K RNA polymerase II, I and III subunit K (human), Gene ID: 5440. Updated 2025.
ANKRD1	Ankyrin-repeat transcriptional regulator responsive to stress/mechanical signals.	Kojic S, et al. “Ankrd1/carp regulates cardiac transcription and function.” Circ Res. 2010;106(8): 1392–1403. PMID: 20339119
IFI35	Interferon-induced protein 35 kDa (IFI35), an interferon-stimulated protein that modulates innate immune and inflammatory signalling; here considered as a stress/inflammation-related regulator within the steroid-responsive context.	De Masi R, Orlando S, Bagordo F, Grassi T. IFP35 Is a Relevant Factor in Innate Immunity, Multiple Sclerosis, and Other Chronic Inflammatory Diseases: Review. Biology (Basel). 2021;10(12):1325. Published 2021 Dec 14. doi:10.3390/biology10121325
KCND1	Voltage-gated potassium channel affecting membrane excitability and signaling.	Rosati B & McKinnon D. “Regulation of ion channel expression.” Circ Res. 2004;94: 874–883. PMID: 15059933
MRM2	Mitochondrial rRNA methyltransferase required for mitoribosome assembly and function.	Lee KW et al. “Human MRM2 methylates U1369 of 16S mitochondrial rRNA.” Nucleic Acids Res. 2013;41(11): 5484–5493. PMID: 23620290
WNT4 / WNT6	Canonical Wnt ligands driving ovarian-promoting and anti-androgenic developmental programs.	Wei M, Zhang C, Tian Y, Du X, Wang Q, Zhao H. Expression and Function of WNT6: From Development to Disease. Front Cell Dev Biol. 2020;8:558155. Published 2020 Dec 9. doi:10.3389/fcell.2020.558155<br>Jeays-Ward K, Dandonneau M, Swain A. Wnt4 is required for proper male as well as female sexual development. Dev Biol. 2004;276(2):431-440. doi:10.1016/j.ydbio.2004.08.049
NCOA4	Nuclear coactivator and ferritinophagy mediator; modulates iron availability and mitochondrial function, indirectly impacting steroidogenesis.	Bellelli R, et al. *NCOA4-mediated ferritinophagy: a cargo-centric pathway regulating intracellular iron homeostasis.* Nat Commun. 2016;7:12594. PMID: 27596085
GMPPB	GDP-mannose pyrophosphorylase subunit B, essential for protein glycosylation; defects alter signalling pathways including hormone receptor stability.	Carss KJ, et al. *Mutations in GDP-mannose pyrophosphorylase B cause congenital and limb-girdle muscular dystrophies associated with hypoglycosylation of α-dystroglycan.* Am J Hum Genet. 2013;93(1):29–41. PMID: 23768512
HADH	Mitochondrial 3-hydroxyacyl-CoA dehydrogenase involved in fatty-acid oxidation; influences redox balance and energy supply for steroidogenesis.	Clayton PT. *Disorders of fatty acid oxidation.* J Inherit Metab Dis. 2001;24(2):121–126. PMID: 11392433

System-level meaning	Proteins
Altered developmental morphogen signalling intersecting with androgen pathways	WNT4 (up), WNT6 (up), SALL1 (up), PASK (up), GDF10 (down), RAC1 (down), VEZT (down), CCN5 (up)
Disrupted precursor androgen synthesis with compensatory activation of later steps	HSD3B2 (down), HSD17B3 (up), SULT2B1 (up), UGDH (up), DHRS9 (down), NR4A2 (up), EIF2B3 (down), RPS6 (down), HAO1 (down), NCOA4 (down)
Altered steroidogenic enzyme regulation and gonadal signalling cues	CYP17A1 (down), DAZL (down), BMP15 (NA), CYP27A1 (up), PGR (NA), NUDT1 (up), DHH (up)
Rewiring of transcriptional co-activation and mitochondrial translation networks	MED1 (unchanged), MRPL16 (up), MRPL17 (up), MRPL47 (down), LARS2 (down), MRM2 (down), KCND1 (down), TARS2 (up), DOCK10 (down), ABCA1 (unchanged), BIRC6 (up), CHD9 (down), SERBP1 (down), VEZT (down), POLR2K (down), IFI35 (up), WDR31 (NA), H4C6 (NA)
Shift in androgen activation/inactivation balance	SRD5A2 (down), NFYB (down)

Androgen biosynthetic process

stringId_A	stringId_B	preferredName_A	ensembl_gene_id_A	log2FoldChange_A	preferredName_B	ensembl_gene_id_B	log2FoldChange_B	score
9606.ENSP00000201586	9606.ENSP00000445122	SULT2B1	ENSG00000088002	0.487528685865514	HSD3B2	ENSG00000203859	-0.833156486123551	0.942
9606.ENSP00000364412	9606.ENSP00000445122	HSD17B3	ENSG00000130948	1.00017849812754	HSD3B2	ENSG00000203859	-0.833156486123551	0.987
9606.ENSP00000288937	9606.ENSP00000300651	MRPL17	ENSG00000158042	0.710576846930335	MED1	ENSG00000125686	-0.0198232731764219	0.983
9606.ENSP00000300151	9606.ENSP00000300651	MRPL16	ENSG00000166902	0.10409233244335	MED1	ENSG00000125686	-0.0198232731764219	0.83
9606.ENSP00000300651	9606.ENSP00000417602	MED1	ENSG00000125686	-0.0198232731764219	MRPL47	ENSG00000136522	-0.115294678508557	0.848
9606.ENSP00000300651	9606.ENSP00000363308	MED1	ENSG00000125686	-0.0198232731764219	WDR31	ENSG00000148225	NA	0.916
9606.ENSP00000300651	9606.ENSP00000395590	MED1	ENSG00000125686	-0.0198232731764219	IFI35	ENSG00000068079	0.138029336432692	0.915
9606.ENSP00000244537	9606.ENSP00000300651	H4C6	ENSG00000274618	NA	MED1	ENSG00000125686	-0.0198232731764219	0.913
9606.ENSP00000300651	9606.ENSP00000410083	MED1	ENSG00000125686	-0.0198232731764219	VEZT	ENSG00000028203	-0.824755278959427	0.782
9606.ENSP00000300651	9606.ENSP00000342889	MED1	ENSG00000125686	-0.0198232731764219	POLR2K	ENSG00000147669	-0.02123600753193	0.814
9606.ENSP00000300651	9606.ENSP00000396345	MED1	ENSG00000125686	-0.0198232731764219	CHD9	ENSG00000177200	-0.0710073592582278	0.702
9606.ENSP00000300651	9606.ENSP00000360034	MED1	ENSG00000125686	-0.0198232731764219	SERBP1	ENSG00000142864	-0.195860136160896	0.761

score = 0.7

Androgen receptor signalling pathway

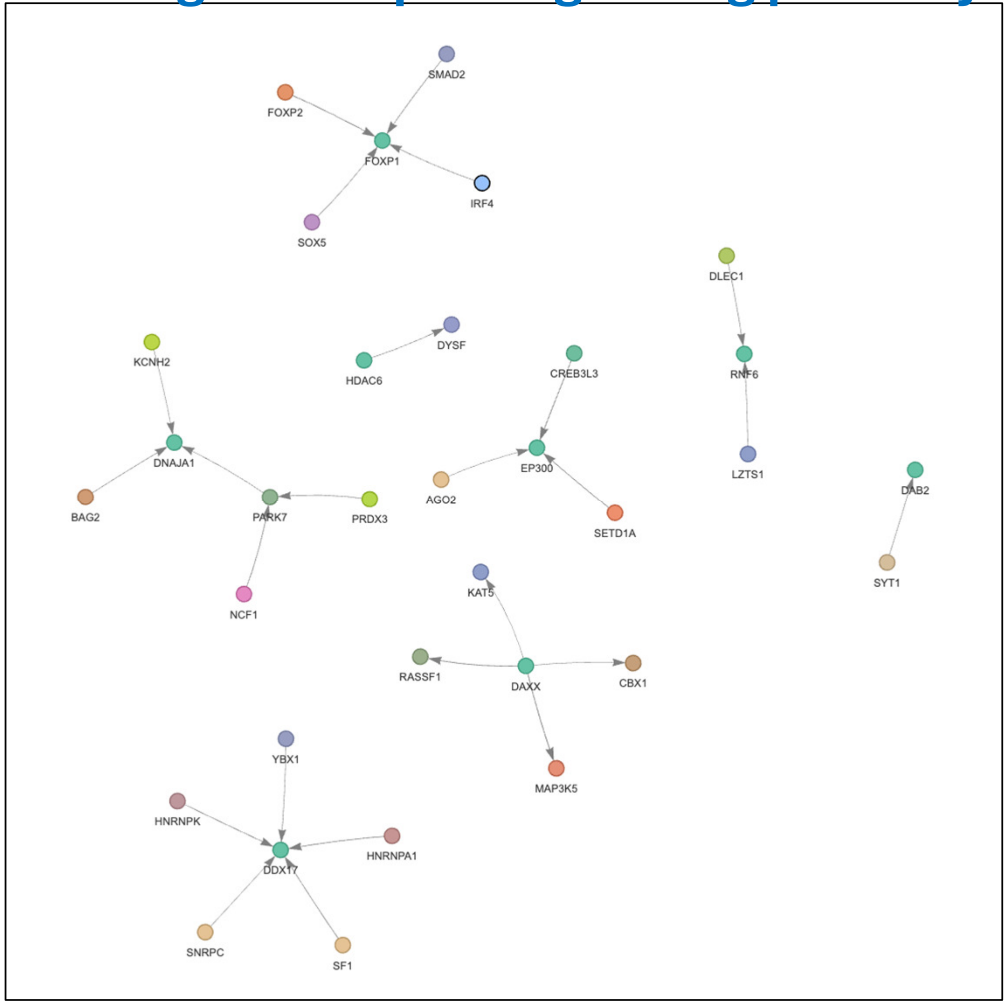

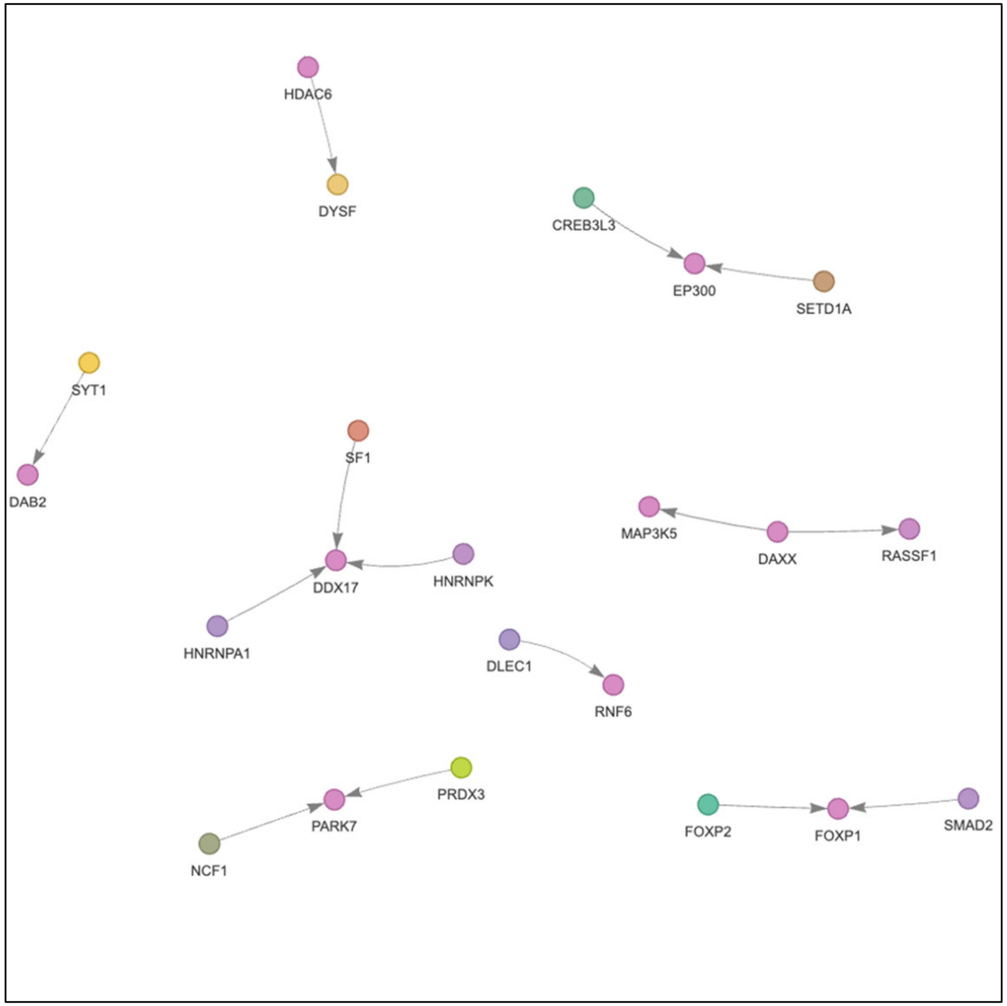

score = 0.4

score = 0.7

Androgen receptor signalling pathway

stringId_A	stringId_B	preferredName_A	ensembl_gene_id_A	log2FoldChange_A	preferredName_B	ensembl_gene_id_B	log2FoldChange_B	score
**9606.ENSP00000266000**	9606.ENSP00000351908	DAXX	ENSG00000204209	0.0638010850766978	MAP3K5	ENSG00000197442	0.460236902928167	0.999
**9606.ENSP00000266000**	9606.ENSP00000349547	DAXX	ENSG00000204209	0.0638010850766978	RASSF1	ENSG00000068028	0.802651209001862	0.948
**9606.ENSP00000078445**	9606.ENSP00000263253	CREB3L3	ENSG00000060566	-0.640357750341971	EP300	ENSG00000100393	-0.0398553990391347	0.912
**9606.ENSP00000386200**	9606.ENSP00000484803	FOXP2	ENSG00000128573	0.625528881948343	FOXP1	ENSG00000114861	-0.562759703536269	0.897
**9606.ENSP00000341826**	9606.ENSP00000380033	HNRNPA1	ENSG00000135486	0.246390072811463	DDX17	ENSG00000100201	0.190788160408698	0.865
**9606.ENSP00000366604**	9606.ENSP00000380033	SF1	ENSG00000168066	0.66044542373951	DDX17	ENSG00000100201	0.190788160408698	0.859
**9606.ENSP00000262519**	9606.ENSP00000263253	SETD1A	ENSG00000099381	0.212828269329481	EP300	ENSG00000100393	-0.0398553990391347	0.854
**9606.ENSP00000289473**	9606.ENSP00000340278	NCF1	ENSG00000158517	0.0158466096542398	PARK7	ENSG00000116288	0.139644690249654	0.835
**9606.ENSP00000365458**	9606.ENSP00000380033	HNRNPK	ENSG00000165119	0.126828305597743	DDX17	ENSG00000100201	0.190788160408698	0.822
**9606.ENSP00000262160**	9606.ENSP00000484803	SMAD2	ENSG00000175387	-0.151303050398991	FOXP1	ENSG00000114861	-0.562759703536269	0.784
**9606.ENSP00000308597**	9606.ENSP00000371000	DLEC1	ENSG00000008226	-0.447945365652523	RNF6	ENSG00000127870	0.0156703603999244	0.756
**9606.ENSP00000298510**	9606.ENSP00000340278	PRDX3	ENSG00000165672	-0.176481521629766	PARK7	ENSG00000116288	0.139644690249654	0.746
**9606.ENSP00000340278**	9606.ENSP00000369127	PARK7	ENSG00000116288	0.139644690249654	DNAJA1	ENSG00000086061	0.653713495570871	0.724
**9606.ENSP00000359727**	9606.ENSP00000369127	BAG2	ENSG00000112208	-1.19504760196443	DNAJA1	ENSG00000086061	0.653713495570871	0.713
**9606.ENSP00000261205**	9606.ENSP00000313391	SYT1	ENSG00000067715	0.0159922200279733	DAB2	ENSG00000153071	0.414302756448581	0.71
**9606.ENSP00000365804**	9606.ENSP00000386881	HDAC6	ENSG00000094631	0.436997669149432	DYSF	ENSG00000135636	-1.25670612058719	0.708
**9606.ENSP00000370343**	9606.ENSP00000484803	IRF4	ENSG00000137265	NA	FOXP1	ENSG00000114861	-0.562759703536269	0.69
**9606.ENSP00000262186**	9606.ENSP00000369127	KCNH2	ENSG00000055118	0.0132505988118139	DNAJA1	ENSG00000086061	0.653713495570871	0.683
**9606.ENSP00000361626**	9606.ENSP00000380033	YBX1	ENSG00000065978	-0.259080766205886	DDX17	ENSG00000100201	0.190788160408698	0.682
**9606.ENSP00000370981**	9606.ENSP00000371000	LZTS1	ENSG00000061337	0.216366535766903	RNF6	ENSG00000127870	0.0156703603999244	0.67
**9606.ENSP00000398273**	9606.ENSP00000484803	SOX5	ENSG00000134532	0.109547743998372	FOXP1	ENSG00000114861	-0.562759703536269	0.639
**9606.ENSP00000220592**	9606.ENSP00000263253	AGO2	ENSG00000123908	-2.44027600218733	EP300	ENSG00000100393	-0.0398553990391347	0.633
**9606.ENSP00000244520**	9606.ENSP00000380033	SNRPC	ENSG00000124562	-0.328885922015605	DDX17	ENSG00000100201	0.190788160408698	0.628
**9606.ENSP00000266000**	9606.ENSP00000377060	DAXX	ENSG00000204209	0.0638010850766978	CBX1	ENSG00000108468	0.20529632762675	0.626
**9606.ENSP00000266000**	9606.ENSP00000340330	DAXX	ENSG00000204209	0.0638010850766978	KAT5	ENSG00000172977	0.803905315605387	0.606

score = 0.4

Protein (STRING name)	Functional definition	Reference
**DAXX**	Death domain-associated protein 6; Transcription corepressor known to repress transcriptional potential of several sumoylated transcription factors.	UniProt Consortium. DAXX – Death domain-associated protein 6 (Q9UER7). UniProtKB/Swiss-Prot. Available at: https://www.uniprot.org/uniprot/Q9UER7. Accessed 2025.
**MAP3K5**	MAP3K5, also known as apoptosis signal-regulating kinase 1 (ASK1), is a key stress-responsive mitogen-activated protein kinase kinase kinase that integrates a variety of intracellular and extracellular stimuli to regulate cell fate.	Ichijo H, Nishida E, Irie K, et al. Induction of apoptosis by ASK1, a mammalian MAPKKK that activates SAPK/JNK and p38 signaling pathways. Science. 1997;275(5296):90-94. doi:10.1126/science.275.5296.90
**RASSF1**	Tumor suppressor interacting with MAP3K pathways and nuclear signaling modules; regulates apoptosis and transcriptional responses.	Matallanas D, Romano D, Yee K, et al. RASSF1A elicits apoptosis through an MST2 pathway directing proapoptotic transcription by the p73 tumor suppressor protein. Mol Cell. 2007;27(6):962-975. doi:10.1016/j.molcel.2007.08.008
**CBX1**	Chromobox protein involved in heterochromatin formation and transcriptional regulation, interacting with nuclear receptor complexes.	Iwata-Otsubo, A., Dias, K.-R., Su, C. et al. (2020) 'CBX1 variants cause a neurodevelopmental syndrome due to facultative heterochromatin dysfunction', bioRxiv. doi: 10.1101/2020.09.29.319228.
**KAT5**	Histone acetyltransferase involved in chromatin remodeling; modulates AR and ER transcription through acetylation of nuclear targets.	Agrawal A, Sikder S, Singh S, et al. KAT5-mediated acetylation of PC4 facilitates DNA repair by promoting chromatin reorganization. Nucleic Acids Res. 2025;53(18):gkaf974. doi:10.1093/nar/gkaf974
**DDX17**	RNA helicase functioning as a co-regulator of AR and ER, modulating spliceosome activity and chromatin-associated transcription.	Samaan S, Tranchevent LC, Dardenne E, et al. The Ddx5 and Ddx17 RNA helicases are cornerstones in the complex regulatory array of steroid hormone-signaling pathways. Nucleic Acids Res. 2014;42(4):2197-2207. doi:10.1093/nar/gkt1216
**HNRNPA1**	RNA-binding protein involved in splicing and RNA processing; interacts with nuclear receptor–regulated transcripts.	Bonomi S, di Matteo A, Buratti E, et al. HnRNP A1 controls a splicing regulatory circuit promoting mesenchymal-to-epithelial transition. Nucleic Acids Res. 2013;41(18):8665-8679. doi:10.1093/nar/gkt579
**HNRNPK**	Multifunctional RNA- and DNA-binding protein with roles in chromatin remodeling and steroid receptor transcriptional regulation.	Xu Y, Li R, Zhang K, et al. The multifunctional RNA-binding protein hnRNPK is critical for the proliferation and differentiation of myoblasts. BMB Rep. 2018;51(7):350-355. doi:10.5483/bmbrep.2018.51.7.043
**SF1**	Steroidogenic transcription factor influencing AR activity and supporting transcription of steroidogenic enzymes.	Naamneh Elzenaty R, Kouri C, Martinez de Lapiscina I, et al. NR5A1/SF-1 Collaborates with Inhibin α and the Androgen Receptor. Int J Mol Sci. 2024;25(18):10109. Published 2024 Sep 20. doi:10.3390/ijms251810109
**YBX1**	DNA/RNA-binding protein influencing chromatin accessibility and transcription; interacts with hormone receptor regulatory networks.	Zheng X, Zeng F, Lei Y, et al. YBX1: an RNA/DNA-binding protein that affects disease progression. Front Oncol. 2025;15:1635209. Published 2025 Jul 29. doi:10.3389/fonc.2025.1635209
SNRPC	Core spliceosomal protein of the U1 snRNP, mediating 5' splice-site recognition; contributes to post-transcriptional regulation in steroid-responsive programs.	Rossi F., et al. (1996) *Involvement of U1 Small Nuclear Ribonucleoproteins (snRNP) in 5' Splice Site–U1 snRNP Interaction.* Journal of Biological Chemistry, 271(39):23985–23991.
EP300	Chromatin acetyltransferase and nuclear coactivator required for AR engagement with chromatin.	Goodman RH & Smolik S. "CBP/p300 in cell growth, transformation, and development." Genes Dev. 2000;14:1553–1577. PMID: 10887150
SETD1A	Histone H3K4 methyltransferase involved in transcriptional activation of nuclear receptor target genes.	Lee JH, Skalnik DG. Wdr82 is a C-terminal domain-binding protein that recruits the Setd1A Histone H3-Lys4 methyltransferase complex to transcription start sites of transcribed human genes. Mol Cell Biol. 2008;28(2):609-618. doi:10.1128/MCB.01356-07
CREB3L3	Stress-responsive ER-bound transcription factor regulating metabolic gene expression; linked to steroid-regulated metabolic responses.	Nakagawa Y, Satoh A, Yabe S, et al. Hepatic CREB3L3 controls whole-body energy homeostasis and improves obesity and diabetes. Endocrinology. 2014;155(12):4706-4719. doi:10.1210/en.2014-1113
AGO2	Catalytic core of RISC complex; mediates microRNA-dependent post-transcriptional repression and affects nuclear receptor outputs.	Johnson KC, Kilikevicius A, Hofman C, et al. Nuclear localization of Argonaute 2 is affected by cell density and may relieve repression by microRNAs. Nucleic Acids Res. 2024;52(4):1930-1952. doi:10.1093/nar/gkad1155
**DNAJA1**	HSP40 co-chaperone required for proper folding and nuclear import of AR; enhances AR stability.	Terada, K., Yomogida, K., Imai, T. et al. A type I DnaJ homolog, DjA1, regulates androgen receptor signaling and spermatogenesis. EMBO J 24, 611–622 (2005). https://doi.org/10.1038/sj.emboj.7600549
**PARK7**	Parkinson's disease protein PARK7 prevents metabolite and protein damage caused by a glycolytic metabolite.	Heremans IP, Caligiore F, Gerin I, et al. Parkinson's disease protein PARK7 prevents metabolite and protein damage caused by a glycolytic metabolite. Proc Natl Acad Sci U S A. 2022;119(4):e2111338119. doi:10.1073/pnas.2111338119
**BAG2**	Co-chaperone inhibiting HSP70 activity; regulates folding stability of nuclear receptors.	Arndt V, Daniel C, Nastainczyk W, Alberti S, Höhfeld J. BAG-2 acts as an inhibitor of the chaperone-associated ubiquitin ligase CHIP. Mol Biol Cell. 2005;16(12):5891-5900. doi:10.1091/mbc.e05-07-0660
**PRDX3**	Mitochondrial peroxiredoxin involved in redox regulation, modulating oxidative conditions that influence AR stability.	Cox AG, Pullar JM, Hughes G, Ledgerwood EC, Hampton MB. Oxidation of mitochondrial peroxiredoxin 3 during the initiation of receptor-mediated apoptosis. Free Radic Biol Med. 2008;44(6):1001-1009. doi:10.1016/j.freeradbiomed.2007.11.017
**SYT1**	Synaptotagmin involved in vesicle trafficking; here part of a module reflecting trafficking and signalling dynamics.	Quiñones-Frías MC, Littleton JT. Function of Drosophila Synaptotagmins in membrane trafficking at synapses. Cell Mol Life Sci. 2021;78(9):4335-4364. doi:10.1007/s00018-021-03788-9
**DAB2**	Adaptor protein modulating endocytosis, trafficking, and MAPK signaling, contributing to AR regulatory environment.	Chetrit D, Barzilay L, Horn G, Bielik T, Smorodinsky NI, Ehrlich M. Negative regulation of the endocytic adaptor disabled-2 (Dab2) in mitosis. J Biol Chem. 2011;286(7):5392-5403. doi:10.1074/jbc.M110.161851
**KCNH2**	Voltage-gated potassium channel involved in electrical signaling; connected here through interactions affecting cellular excitability and stress responses.	Zheng Z, Song Y. Integrated analysis of the voltage-gated potassium channel-associated gene KCNH2 across cancers. BMC Bioinformatics. 2023;24(1):51. Published 2023 Feb 15. doi:10.1186/s12859-023-05180-9
**FOXP1**	AR-modulating forkhead transcription factor; downregulation shifts nuclear receptor specificity.	Takayama K, Horie-Inoue K, Ikeda K, et al. FOXP1 is an androgen-responsive transcription factor that negatively regulates androgen receptor signaling in prostate cancer cells. Biochem Biophys Res Commun. 2008;374(2):388-393. doi:10.1016/j.bbrc.2008.07.056
**FOXP2**	Forkhead transcription factor affecting nuclear transcriptional balance; upregulation alters AR regulatory circuitry.	Morgan A, Fisher SE, Scheffer I, et al. FOXP2-Related Speech and Language Disorder. 2016 Jun 23 [Updated 2023 Jan 26]. In: Adam MP, Bick S, Mirzaa GM, et al., editors. GeneReviews® [Internet]. Seattle (WA): University of Washington, Seattle; 1993-2025. Available from: https://www.ncbi.nlm.nih.gov/books/NBK368474/
**SMAD2**	TGF-β signalling effector interacting with AR and modulating transcriptional crosstalk between pathways.	Luo K. Signaling Cross Talk between TGF-β/Smad and Other Signaling Pathways. Cold Spring Harb Perspect Biol. 2017;9(1):a022137. Published 2017 Jan 3. doi:10.1101/cshperspect.a022137
**DLEC1**	Tumor suppressor interacting with ubiquitin ligases and signalling nodes affecting receptor turnover.	Qiu GH, Xie X, Deng L, Hooi SC. Tumor Suppressor DLEC1 can Stimulate the Proliferation of Cancer Cells When AP-2α2 is Down-Regulated in HCT116. Hepat Mon. 2015;15(11):e29829. Published 2015 Nov 28. doi:10.5812/hepatmon.29829
**RNF6**	E3 ubiquitin ligase modifying AR and enhancing transcriptional activity via ubiquitination.	Xu K, Shimelis H, Linn DE, et al. Regulation of androgen receptor transcriptional activity and specificity by RNF6-induced ubiquitination. Cancer Cell. 2009;15(4):270-282. doi:10.1016/j.ccr.2009.02.021
**HDAC6**	Cytoplasmic histone deacetylase regulating AR stability, trafficking, and chaperone interactions.	Valenzuela-Fernández A, Cabrero JR, Serrador JM, Sánchez-Madrid F. HDAC6: a key regulator of cytoskeleton, cell migration and cell-cell interactions. Trends Cell Biol. 2008;18(6):291-297. doi:10.1016/j.tcb.2008.04.003
**DYSF**	Dysferlin involved in membrane repair; downregulation indicates altered proteostasis and stress signaling.	Defour A, Van der Meulen JH, Bhat R, et al. Dysferlin regulates cell membrane repair by facilitating injury-triggered acid sphingomyelinase secretion. Cell Death Dis. 2014;5(6):e1306. Published 2014 Jun 26. doi:10.1038/cddis.2014.272
**LZTS1**	Tumor suppressor influencing cell-cycle control and transcriptional regulation.	Lovat F, Ishii H, Schiappacassi M, et al. LZTS1 downregulation confers paclitaxel resistance and is associated with worse prognosis in breast cancer. Oncotarget. 2014;5(4):970-977. doi:10.18632/oncotarget.1630

**System-level meaning**	**Proteins**
Altered co-regulator recruitment and stress-response signalling influencing AR function	DAXX (up), MAP3K5 (up), RASSF1 (up), CBX1 (up), KAT5 (up)
Reduced transcriptional co-activation capacity affecting AR-driven gene expression	CREB3L3 (down), EP300 (down), SETD1A (up), AGO2 (down)
Perturbed FOXP transcription factor axis modulating AR chromatin binding	FOXP2 (up), FOXP1 (down), IRF4 (NA), SOX5 (up), SMAD2 (down)
Altered RNA-processing and RNP dynamics impacting AR transcription cycles	HNRNPA1 (up), HNRNPK (up), YBX1 (down), SNRPC (down), DDX17 (up), SF1 (up)
Oxidative-stress response shift influencing AR post-translational activation	PARK7 (up), PRDX3 (down), NCF1 (up), DNAJA1 (up), BAG2 (down), KCNH2 (up)
Nucleocytoplasmic trafficking of AR	HDAC6 (up), DYSF (down)
Ubiquitin-dependent modulation of AR receptor turnover	RNF6 (up), DLEC1 (down), LZTS1 (up)
Vesicle-trafficking associated modulation of AR nuclear shuttling	SYT1 (up), DAB2 (up)

Androgen receptor signalling pathway

stringId_A	stringId_B	preferredName_A	ensembl_gene_id_A	log2FoldChange_A	preferredName_B	ensembl_gene_id_B	log2FoldChange_B	score
9606.ENSP00000266000	9606.ENSP00000351908	DAXX	ENSG00000204209	0.0638010850766978	MAP3K5	ENSG00000197442	0.460236902928167	0.999
9606.ENSP00000266000	9606.ENSP00000349547	DAXX	ENSG00000204209	0.0638010850766978	RASSF1	ENSG00000068028	0.802651209001862	0.948
9606.ENSP00000078445	9606.ENSP00000263253	CREB3L3	ENSG00000060566	-0.640357750341971	EP300	ENSG00000100393	-0.0398553990391347	0.912
9606.ENSP00000386200	9606.ENSP00000484803	FOXP2	ENSG00000128573	0.625528881948343	FOXP1	ENSG00000114861	-0.562759703536269	0.897
9606.ENSP00000341826	9606.ENSP00000380033	HNRNPA1	ENSG00000135486	0.246390072811463	DDX17	ENSG00000100201	0.190788160408698	0.865
9606.ENSP00000366604	9606.ENSP00000380033	SF1	ENSG00000168066	0.66044542373951	DDX17	ENSG00000100201	0.190788160408698	0.859
9606.ENSP00000262519	9606.ENSP00000263253	SETD1A	ENSG00000099381	0.212828269329481	EP300	ENSG00000100393	-0.0398553990391347	0.854
9606.ENSP00000289473	9606.ENSP00000340278	NCF1	ENSG00000158517	0.0158466096542398	PARK7	ENSG00000116288	0.139644690249654	0.835
9606.ENSP00000365458	9606.ENSP00000380033	HNRNPK	ENSG00000165119	0.126828305597743	DDX17	ENSG00000100201	0.190788160408698	0.822
9606.ENSP00000262160	9606.ENSP00000484803	SMAD2	ENSG00000175387	-0.151303050398991	FOXP1	ENSG00000114861	-0.562759703536269	0.784
9606.ENSP00000308597	9606.ENSP00000371000	DLEC1	ENSG00000008226	-0.447945365652523	RNF6	ENSG00000127870	0.0156703603999244	0.756
9606.ENSP00000298510	9606.ENSP00000340278	PRDX3	ENSG00000165672	-0.176481521629766	PARK7	ENSG00000116288	0.139644690249654	0.746
9606.ENSP00000261205	9606.ENSP00000313391	SYT1	ENSG00000067715	0.0159922200279733	DAB2	ENSG00000153071	0.414302756448581	0.71
9606.ENSP00000365804	9606.ENSP00000386881	HDAC6	ENSG00000094631	0.436997669149432	DYSF	ENSG00000135636	-1.25670612058719	0.708

score = 0.7

Regulation of androgen receptor signalling pathway

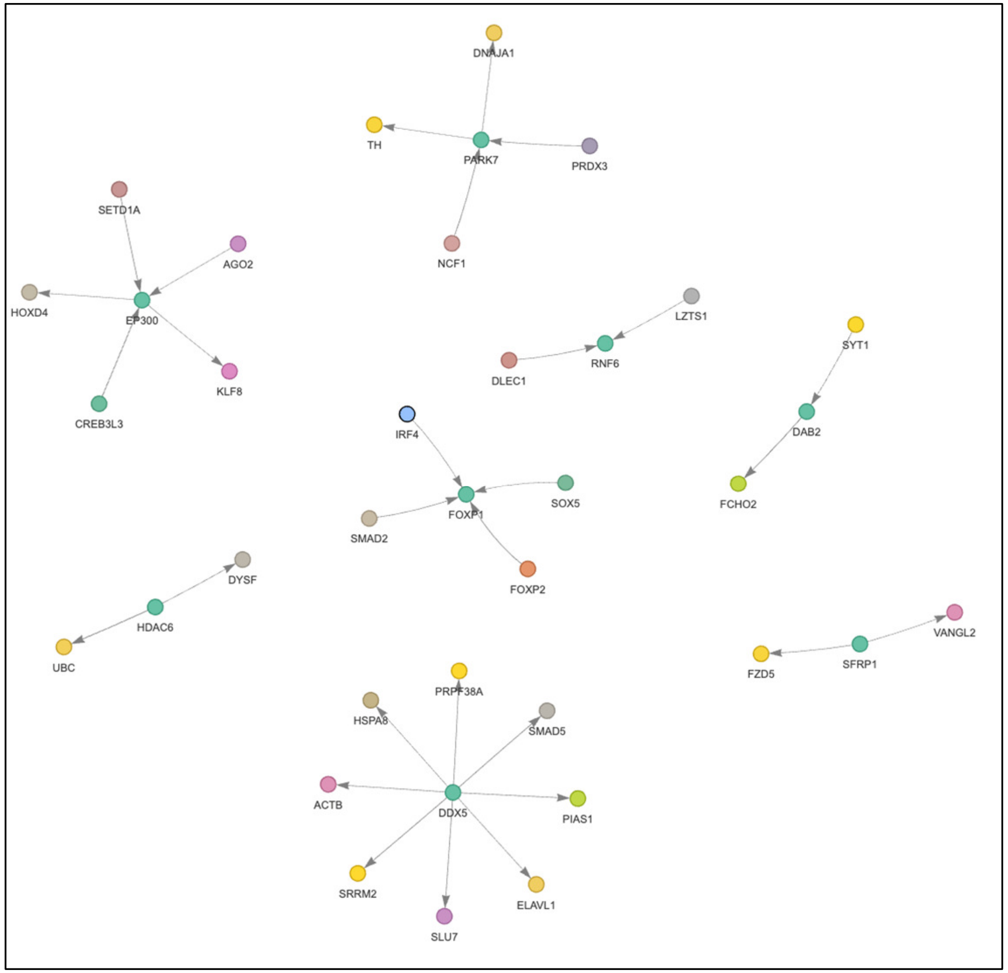

score = 0.4

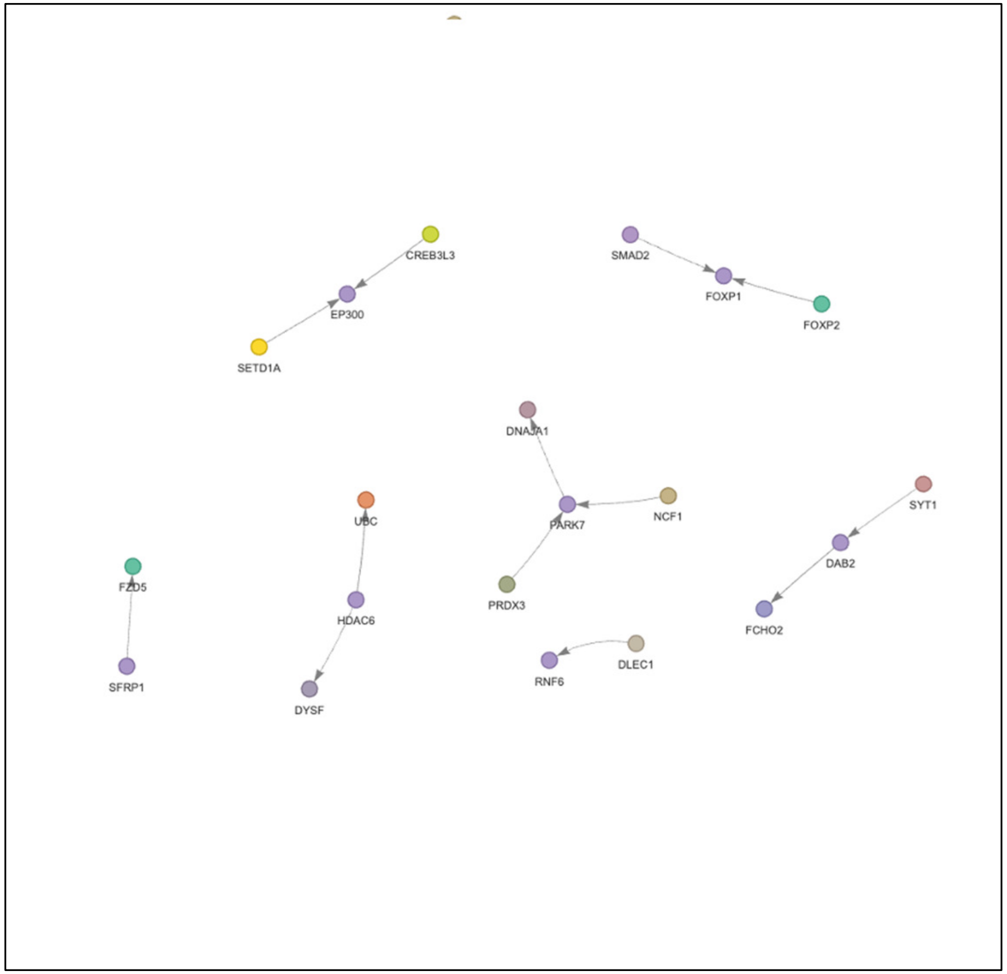

score = 0.7

selected less nodes

Regulation of androgen receptor signalling pathway

stringId_A	stringId_B	preferredName_A	ensembl_gene_id_A	log2FoldChange_A	preferredName_B	ensembl_gene_id_B	log2FoldChange_B	score
**9606.ENSP00000365804**	9606.ENSP00000441543	HDAC6	ENSG00000094631	0.436997669149432	UBC	ENSG00000150991	-0.312034852868253	0.995
**9606.ENSP00000313391**	9606.ENSP00000393776	DAB2	ENSG00000153071	0.414302756448581	FCHO2	ENSG00000157107	-0.00171763033896641	0.968
**9606.ENSP00000225792**	9606.ENSP00000301740	DDX5	ENSG00000108654	-0.290947572571089	SRRM2	ENSG00000167978	-0.208030314387177	0.912
**9606.ENSP00000078445**	9606.ENSP00000263253	CREB3L3	ENSG00000060566	-0.640357750341971	EP300	ENSG00000100393	-0.0398553990391347	0.912
**9606.ENSP00000386200**	9606.ENSP00000484803	FOXP2	ENSG00000128573	0.625528881948343	FOXP1	ENSG00000114861	-0.562759703536269	0.897
**9606.ENSP00000225792**	9606.ENSP00000441954	DDX5	ENSG00000108654	-0.290947572571089	SMAD5	ENSG00000113658	0.0125480292024104	0.882
**9606.ENSP00000225792**	9606.ENSP00000297151	DDX5	ENSG00000108654	-0.290947572571089	SLU7	ENSG00000164609	0.0224408238709146	0.855
**9606.ENSP00000262519**	9606.ENSP00000263253	SETD1A	ENSG00000099381	0.212828269329481	EP300	ENSG00000100393	-0.0398553990391347	0.854
**9606.ENSP00000289473**	9606.ENSP00000340278	NCF1	ENSG00000158517	0.0158466096542398	PARK7	ENSG00000116288	0.139644690249654	0.835
**9606.ENSP00000225792**	9606.ENSP00000257181	DDX5	ENSG00000108654	-0.290947572571089	PRPF38A	ENSG00000134748	0.195842881978449	0.826
**9606.ENSP00000262160**	9606.ENSP00000484803	SMAD2	ENSG00000175387	-0.151303050398991	FOXP1	ENSG00000114861	-0.562759703536269	0.784
**9606.ENSP00000308597**	9606.ENSP00000371000	DLEC1	ENSG00000008226	-0.447945365652523	RNF6	ENSG00000127870	0.0156703603999244	0.756
**9606.ENSP00000225792**	9606.ENSP00000438574	DDX5	ENSG00000108654	-0.290947572571089	PIAS1	ENSG00000033800	0.665353020788877	0.752
**9606.ENSP00000298510**	9606.ENSP00000340278	PRDX3	ENSG00000165672	-0.176481521629766	PARK7	ENSG00000116288	0.139644690249654	0.746
**9606.ENSP00000220772**	9606.ENSP00000354607	SFRP1	ENSG00000104332	-1.06181186373758	FZD5	ENSG00000163251	-1.9423149844083	0.728
**9606.ENSP00000340278**	9606.ENSP00000369127	PARK7	ENSG00000116288	0.139644690249654	DNAJA1	ENSG00000086061	0.653713495570871	0.724
**9606.ENSP00000225792**	9606.ENSP00000494750	DDX5	ENSG00000108654	-0.290947572571089	ACTB	ENSG00000075624	-0.396064192020913	0.713
**9606.ENSP00000261205**	9606.ENSP00000313391	SYT1	ENSG00000067715	0.0159922200279733	DAB2	ENSG00000153071	0.414302756448581	0.71
**9606.ENSP00000365804**	9606.ENSP00000386881	HDAC6	ENSG00000094631	0.436997669149432	DYSF	ENSG00000135636	-1.25670612058719	0.708
**9606.ENSP00000225792**	9606.ENSP00000385269	DDX5	ENSG00000108654	-0.290947572571089	ELAVL1	ENSG00000066044	-0.119678748353951	0.703
**9606.ENSP00000225792**	9606.ENSP00000437125	DDX5	ENSG00000108654	-0.290947572571089	HSPA8	ENSG00000109971	0.0971835265834756	0.697
**9606.ENSP00000370343**	9606.ENSP00000484803	IRF4	ENSG00000137265	NA	FOXP1	ENSG00000114861	-0.562759703536269	0.69
**9606.ENSP00000220772**	9606.ENSP00000357040	SFRP1	ENSG00000104332	-1.06181186373758	VANGL2	ENSG00000162738	-0.0248485288694255	0.679
**9606.ENSP00000370981**	9606.ENSP00000371000	LZTS1	ENSG00000061337	0.216366535766903	RNF6	ENSG00000127870	0.0156703603999244	0.67
**9606.ENSP00000340278**	9606.ENSP00000370571	PARK7	ENSG00000116288	0.139644690249654	TH	ENSG00000180176	-1.53986914425978	0.659
**9606.ENSP00000263253**	9606.ENSP00000302548	EP300	ENSG00000100393	-0.0398553990391347	HOXD4	ENSG00000170166	0.387239609500037	0.657
**9606.ENSP00000263253**	9606.ENSP00000417303	EP300	ENSG00000100393	-0.0398553990391347	KLF8	ENSG00000102349	-0.257662879605205	0.653
**9606.ENSP00000398273**	9606.ENSP00000484803	SOX5	ENSG00000134532	0.109547743998372	FOXP1	ENSG00000114861	-0.562759703536269	0.639
**9606.ENSP00000220592**	9606.ENSP00000263253	AGO2	ENSG00000123908	-2.44027600218733	EP300	ENSG00000100393	-0.0398553990391347	0.633

score = 0.4

Protein	Functional definition	Reference
**DDX5**	RNA helicase functioning as a nuclear AR coactivator; regulates AR-dependent transcription, chromatin association, and AR-associated splicing.	Endoh H, Maruyama K, Masuhiro Y, et al. Purification and identification of p68 RNA helicase acting as a transcriptional coactivator specific for the activation function 1 of human estrogen receptor alpha [retracted in: Mol Cell Biol. 2014 Mar;34(5):915. doi: 10.1128/MCB.01458-13.]. Mol Cell Biol. 1999;19(8):5363-5372. doi:10.1128/MCB.19.8.5363
**SRRM2**	Nuclear splicing factor involved in splice-site selection and mRNA maturation; supports AR-linked RNA processing.	Blencowe BJ. Alternative splicing: new insights from global analyses. Cell. 2006;126(1):37-47. doi:10.1016/j.cell.2006.06.023
**SLU7**	Splicing factor ensuring accurate 3' splice-site selection during late-stage spliceosome assembly; contributes to AR-associated post-transcriptional regulation.	Gárate-Rascón M, Recalde M, Rojo C, et al. SLU7: A New Hub of Gene Expression Regulation-From Epigenetics to Protein Stability in Health and Disease. Int J Mol Sci. 2022;23(21):13411. Published 2022 Nov 2. doi:10.3390/ijms232113411
**PRPF38A**	Spliceosomal protein involved in U4/U6–U5 tri-snRNP assembly and splicing regulation.	Xie J, Beickman K, Otte E, Rymond BC. Progression through the spliceosome cycle requires Prp38p function for U4/U6 snRNA dissociation. EMBO J. 1998;17(10):2938-2946. doi:10.1093/emboj/17.10.2938
**ELAVL1**	RNA-binding protein that stabilizes target transcripts and modulates hormone-responsive gene expression.	Abdelmohsen K, Gorospe M. Posttranscriptional regulation of cancer traits by HuR. Wiley Interdiscip Rev RNA. 2010;1(2):214-229. doi:10.1002/wrna.4
**HSPA8**	Constitutive HSP70-family chaperone supporting folding and trafficking of nuclear receptors.	Stricher F, Macri C, Ruff M, Muller S. HSPA8/HSC70 chaperone protein: structure, function, and chemical targeting. Autophagy. 2013;9(12):1937-1954. doi:10.4161/auto.26448
**ACTB**	Actin isoform functioning in nuclear architecture, chromatin regulation, and transcription factor mobility.	Miralles F, Visa N. Actin in transcription and transcription regulation. Curr Opin Cell Biol. 2006;18(3):261-266. doi:10.1016/j.ceb.2006.04.009
**SMAD5**	Signalling effector of BMP/TGF-β pathways that modulates transcription factor networks interacting with AR.	Kretzschmar M, Massagué J. SMADs: mediators and regulators of TGF-beta signaling. Curr Opin Genet Dev. 1998;8(1):103-111. doi:10.1016/s0959-437x(98)80069-5
**EP300**	Chromatin acetyltransferase and nuclear coactivator required for AR engagement with chromatin.	Goodman RH & Smolik S. **“CBP/p300 in cell growth, transformation, and development.”** Genes Dev. 2000;14:1553–1577. **PMID: 10887150**
**CREB3L3**	Stress-responsive ER-bound transcription factor regulating metabolic gene expression; linked to steroid-regulated metabolic responses.	Nakagawa Y, Satoh A, Yabe S, et al. Hepatic CREB3L3 controls whole-body energy homeostasis and improves obesity and diabetes. Endocrinology. 2014;155(12):4706-4719. doi:10.1210/en.2014-1113
**SETD1A**	Histone methyltransferase (H3K4) promoting transcriptional activation of nuclear receptor target genes.	Lee JH, Skalnik DG. Wdr82 is a C-terminal domain-binding protein that recruits the Setd1A Histone H3-Lys4 methyltransferase complex to transcription start sites of transcribed human genes. Mol Cell Biol. 2008;28(2):609-618. doi:10.1128/MCB.01356-07
**HOXD4**	Developmental homeobox transcription factor influencing chromatin context and transcriptional programs relevant to AR.	Rijli FM, Chambon P. Genetic interactions of Hox genes in limb development: learning from compound mutants. Curr Opin Genet Dev. 1997;7(4):481-487. doi:10.1016/s0959-437x(97)80074-3
**KLF8**	Transcription factor controlling cell cycle and transcriptional networks intersecting with AR regulation.	Wang X, Zheng M, Liu G, et al. Krüppel-like factor 8 induces epithelial to mesenchymal transition and epithelial cell invasion. Cancer Res. 2007;67(15):7184-7193. doi:10.1158/0008-5472.CAN-06-4729
**FOXP1**	AR-modulating forkhead transcription factor; downregulation shifts nuclear receptor specificity.	Takayama K, Horie-Inoue K, Ikeda K, et al. FOXP1 is an androgen-responsive transcription factor that negatively regulates androgen receptor signaling in prostate cancer cells. Biochem Biophys Res Commun. 2008;374(2):388-393. doi:10.1016/j.bbrc.2008.07.056
**FOXP2**	Forkhead transcription factor affecting nuclear transcriptional balance; upregulation alters AR regulatory circuitry.	Morgan A, Fisher SE, Scheffer I, et al. FOXP2-Related Speech and Language Disorder. 2016 Jun 23 [Updated 2023 Jan 26]. In: Adam MP, Bick S, Mirzaa GM, et al., editors. GeneReviews® [Internet]. Seattle (WA): University of Washington, Seattle; 1993-2025. Available from: https://www.ncbi.nlm.nih.gov/books/NBK368474/
**PIAS1**	SUMO E3 ligase modifying AR and other nuclear receptors, modulating transcriptional output and nuclear localization.	Kotaja N, Karvonen U, Jänne OA, Palvimo JJ. PIAS proteins modulate transcription factors by functioning as SUMO-1 ligases. Mol Cell Biol. 2002;22(14):5222-5234. doi:10.1128/MCB.22.14.5222-5234.2002
**HDAC6**	Cytoplasmic deacetylase that modulates AR stability, HSP90 activity, and nuclear transport.	Ai J, Wang Y, Dar JA, et al. HDAC6 regulates androgen receptor hypersensitivity and nuclear localization via modulating Hsp90 acetylation in castration-resistant prostate cancer. Mol Endocrinol. 2009;23(12):1963-1972. doi:10.1210/me.2009-0188
**DNAJA1**	HSP40 co-chaperone required for proper folding and nuclear import of AR; enhances AR stability.	Terada, K., Yomogida, K., Imai, T. et al. A type I DnaJ homolog, DjA1, regulates androgen receptor signaling and spermatogenesis. EMBO J 24, 611–622 (2005). https://doi.org/10.1038/sj.emboj.7600549
**PARK7**	Parkinson's disease protein PARK7 prevents metabolite and protein damage caused by a glycolytic metabolite.	Heremans IP, Caligiore F, Gerin I, et al. Parkinson's disease protein PARK7 prevents metabolite and protein damage caused by a glycolytic metabolite. Proc Natl Acad Sci U S A. 2022;119(4):e2111338119. doi:10.1073/pnas.2111338119
**DYSF**	Membrane repair protein; its downregulation reflects altered proteostasis and stress response.	Bulgart HR, Lopez Perez MA, Tucker A, et al. Plasma membrane repair defect in Alzheimer's disease neurons is driven by the reduced dysferlin expression. FASEB J. 2024;38(20):e70099. doi:10.1096/fj.202401731RR
**SFRP1**	Secreted Wnt antagonist linking Wnt–AR crosstalk; downregulation shifts Wnt/PCP balance.	Elzi DJ, Song M, Hakala K, Weintraub ST, Shiio Y. Wnt antagonist SFRP1 functions as a secreted mediator of senescence. Mol Cell Biol. 2012;32(21):4388-4399. doi:10.1128/MCB.06023-11
**FZD5**	Wnt receptor mediating canonical and PCP signaling modules that interface with androgen-regulated developmental programs.	Wright SC, Cañizal MCA, Benkel T, et al. FZD5 is a Gαq-coupled receptor that exhibits the functional hallmarks of prototypical GPCRs. Sci Signal. 2018;11(559):eaar5536. Published 2018 Dec 4. doi:10.1126/scisignal.aar5536
**VANGL2**	Core PCP protein coordinating planar cell polarity and Wnt–PCP signalling, integrated into AR developmental context.	Shafer B, Onishi K, Lo C, Colakoglu G, Zou Y. Vangl2 promotes Wnt/planar cell polarity-like signaling by antagonizing Dvl1-mediated feedback inhibition in growth cone guidance. Dev Cell. 2011;20(2):177-191. doi:10.1016/j.devcel.2011.01.002
**DAB2**	Endocytic adaptor regulating receptor trafficking and signalling, supporting AR-associated pathways.	Chetrit D, Barzilay L, Horn G, Bielik T, Smorodinsky NI, Ehrlich M. Negative regulation of the endocytic adaptor disabled-2 (Dab2) in mitosis. J Biol Chem. 2011;286(7):5392-5403. doi:10.1074/jbc.M110.161851
**FCHO2**	Endocytic protein regulating clathrin-mediated vesicle formation and receptor trafficking.	Henne WM, Boucrot E, Meinecke M, et al. FCHo proteins are nucleators of clathrin-mediated endocytosis. Science. 2010;328(5983):1281-1284. doi:10.1126/science.1188462
**PRDX3**	Mitochondrial peroxiredoxin supporting redox balance and protecting AR-related nuclear processes.	Gomes F, Turano H, Haddad LA, Netto LES. Human mitochondrial peroxiredoxin Prdx3 is dually localized in the intermembrane space and matrix subcompartments. Redox Biol. 2024;78:103436. doi:10.1016/j.redox.2024.103436
**TH**	Rate-limiting enzyme in catecholamine synthesis; influences neuroendocrine tone impacting AR signalling.	Rao F, Zhang L, Wessel J, et al. Tyrosine hydroxylase, the rate-limiting enzyme in catecholamine biosynthesis: discovery of common human genetic variants governing transcription, autonomic activity, and blood pressure in vivo. Circulation. 2007;116(9):993-1006. doi:10.1161/CIRCULATIONAHA.106.682302

**System-level meaning**	**Proteins**
Altered chromatin remodelling and transcriptional modulation of AR	EP300 (down), CREB3L3 (down), SETD1A (up), AGO2 (down), HOXD4 (up), KLF8 (down)
Chaperone-mediated oxidative stress response influencing AR stability	PARK7 (up), PRDX3 (down), NCF1 (up), DNAJA1 (up), TH (down)
Ubiquitin-dependent regulation of AR turnover	RNF6 (up), DLEC1 (down), LZTS1 (up)
Vesicle trafficking and receptor shuttling	DAB2 (up), SYT1 (up), FCHO2 (down)
FOXP transcription factor axis modulating AR chromatin binding	FOXP1 (down), FOXP2 (up), SOX5 (up), SMAD2 (down), IRF4 (NA)
RNA processing and spliceosome-related AR modulation	DDX5 (down), PRPF38A (up), PIAS1 (up), ELAVL1 (down), HSPA8 (up), ACTB (down), SRRM2 (down), SLU7 (up), SMAD5 (up)
Regulation of AR nucleocytoplasmic trafficking and stability	HDAC6 (up), DYSF (down), UBC (down)
Non-canonical WNT signalling affecting AR responsiveness	SFRP1 (down), FZD5 (down), VANGL2 (down)

regulation of androgen receptor signalling pathway

stringId_A	stringId_B	preferredName_A	ensembl_gene_id_A	log2FoldChange_A	preferredName_B	ensembl_gene_id_B	log2FoldChange_B	score
9606.ENSP00000365804	9606.ENSP00000441543	HDAC6	ENSG00000094631	0.436997669149432	UBC	ENSG00000150991	-0.312034852868253	0.995
9606.ENSP00000313391	9606.ENSP00000393776	DAB2	ENSG00000153071	0.414302756448581	FCHO2	ENSG00000157107	-0.00171763033896641	0.968
9606.ENSP00000078445	9606.ENSP00000263253	CREB3L3	ENSG00000060566	-0.640357750341971	EP300	ENSG00000100393	-0.0398553990391347	0.912
9606.ENSP00000386200	9606.ENSP00000484803	FOXP2	ENSG00000128573	0.625528881948343	FOXP1	ENSG00000114861	-0.562759703536269	0.897
9606.ENSP00000262519	9606.ENSP00000263253	SETD1A	ENSG00000099381	0.212828269329481	EP300	ENSG00000100393	-0.0398553990391347	0.854
9606.ENSP00000289473	9606.ENSP00000340278	NCF1	ENSG00000158517	0.0158466096542398	PARK7	ENSG00000116288	0.139644690249654	0.835
9606.ENSP00000262160	9606.ENSP00000484803	SMAD2	ENSG00000175387	-0.151303050398991	FOXP1	ENSG00000114861	-0.562759703536269	0.784
9606.ENSP00000308597	9606.ENSP00000371000	DLEC1	ENSG00000008226	-0.447945365652523	RNF6	ENSG00000127870	0.0156703603999244	0.756
9606.ENSP00000298510	9606.ENSP00000340278	PRDX3	ENSG00000165672	-0.176481521629766	PARK7	ENSG00000116288	0.139644690249654	0.746
9606.ENSP00000220772	9606.ENSP00000354607	SFRP1	ENSG00000104332	-1.06181186373758	FZD5	ENSG00000163251	-1.9423149844083	0.728
9606.ENSP00000340278	9606.ENSP00000369127	PARK7	ENSG00000116288	0.139644690249654	DNAJA1	ENSG00000086061	0.653713495570871	0.724
9606.ENSP00000261205	9606.ENSP00000313391	SYT1	ENSG00000067715	0.0159922200279733	DAB2	ENSG00000153071	0.414302756448581	0.71
9606.ENSP00000365804	9606.ENSP00000386881	HDAC6	ENSG00000094631	0.436997669149432	DYSF	ENSG00000135636	-1.25670612058719	0.708

score = 0.7

Nuclear androgen receptor binding

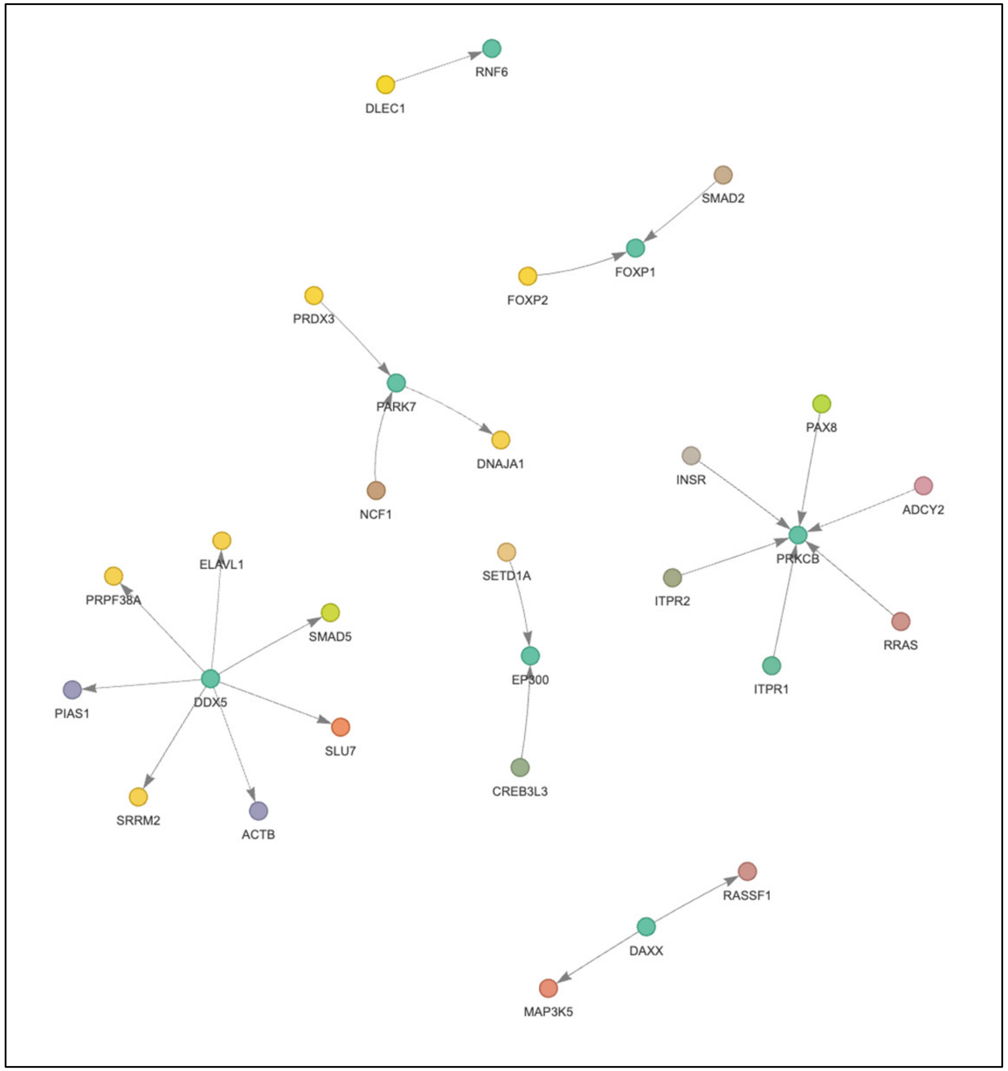

score = 0.4

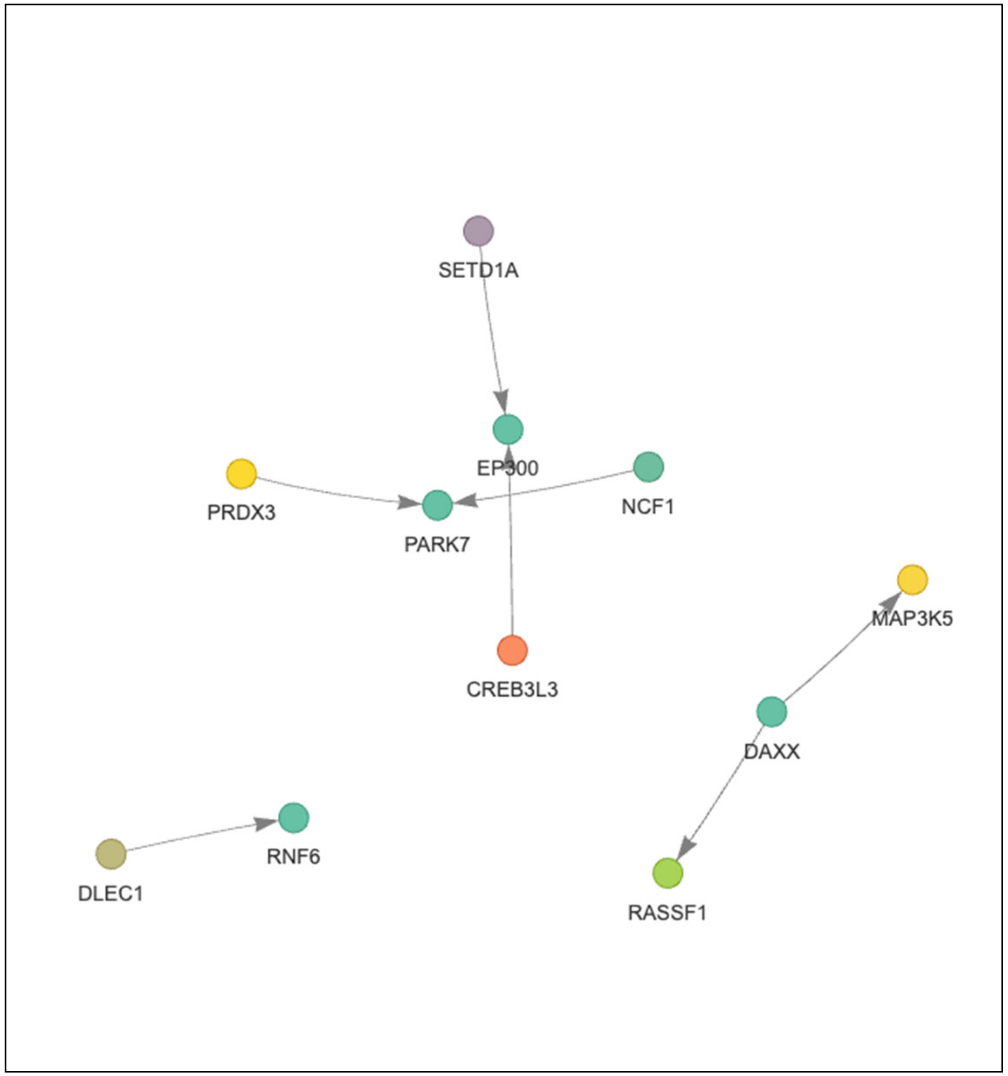

score = 0.7

selected less nodes

Nuclear androgen receptor binding

stringId_A	stringId_B	preferredName_A	ensembl_gene_id_A	log2FoldChange_A	preferredName_B	ensembl_gene_id_B	log2FoldChange_B	score
**9606.ENSP00000266000**	9606.ENSP00000351908	DAXX	ENSG00000204209	0.0638010850766978	MAP3K5	ENSG00000197442	0.460236902928167	0.999
**9606.ENSP00000266000**	9606.ENSP00000349547	DAXX	ENSG00000204209	0.0638010850766978	RASSF1	ENSG00000068028	0.802651209001862	0.948
**9606.ENSP00000306253**	9606.ENSP00000496129	ITPR1	ENSG00000150995	0.71810256072489	PRKCB	ENSG00000166501	-0.634718635887524	0.937
**9606.ENSP00000246792**	9606.ENSP00000496129	RRAS	ENSG00000126458	-0.33764521691585	PRKCB	ENSG00000166501	-0.634718635887524	0.919
**9606.ENSP00000263334**	9606.ENSP00000496129	PAX8	ENSG00000125618	-0.162037281146594	PRKCB	ENSG00000166501	-0.634718635887524	0.918
**9606.ENSP00000370744**	9606.ENSP00000496129	ITPR2	ENSG00000123104	-0.831935155207364	PRKCB	ENSG00000166501	-0.634718635887524	0.917
**9606.ENSP00000225792**	9606.ENSP00000301740	DDX5	ENSG00000108654	-0.290947572571089	SRRM2	ENSG00000167978	-0.208030314387177	0.912
**9606.ENSP00000078445**	9606.ENSP00000263253	CREB3L3	ENSG00000060566	-0.640357750341971	EP300	ENSG00000100393	-0.0398553990391347	0.912
**9606.ENSP00000303830**	9606.ENSP00000496129	INSR	ENSG00000171105	0.281583818619248	PRKCB	ENSG00000166501	-0.634718635887524	0.911
**9606.ENSP00000386200**	9606.ENSP00000484803	FOXP2	ENSG00000128573	0.625528881948343	FOXP1	ENSG00000114861	-0.562759703536269	0.897
**9606.ENSP00000225792**	9606.ENSP00000441954	DDX5	ENSG00000108654	-0.290947572571089	SMAD5	ENSG00000113658	0.0125480292024104	0.882
**9606.ENSP00000225792**	9606.ENSP00000297151	DDX5	ENSG00000108654	-0.290947572571089	SLU7	ENSG00000164609	0.0224408238709146	0.855
**9606.ENSP00000262519**	9606.ENSP00000263253	SETD1A	ENSG00000099381	0.212828269329481	EP300	ENSG00000100393	-0.0398553990391347	0.854
**9606.ENSP00000289473**	9606.ENSP00000340278	NCF1	ENSG00000158517	0.0158466096542398	PARK7	ENSG00000116288	0.139644690249654	0.835
**9606.ENSP00000225792**	9606.ENSP00000257181	DDX5	ENSG00000108654	-0.290947572571089	PRPF38A	ENSG00000134748	0.195842881978449	0.826
**9606.ENSP00000262160**	9606.ENSP00000484803	SMAD2	ENSG00000175387	-0.151303050398991	FOXP1	ENSG00000114861	-0.562759703536269	0.784
**9606.ENSP00000342952**	9606.ENSP00000496129	ADCY2	ENSG00000078295	0.272449985126027	PRKCB	ENSG00000166501	-0.634718635887524	0.768
**9606.ENSP00000308597**	9606.ENSP00000371000	DLEC1	ENSG00000008226	-0.447945365652523	RNF6	ENSG00000127870	0.0156703603999244	0.756
**9606.ENSP00000225792**	9606.ENSP00000438574	DDX5	ENSG00000108654	-0.290947572571089	PIAS1	ENSG00000033800	0.665353020788877	0.752
**9606.ENSP00000298510**	9606.ENSP00000340278	PRDX3	ENSG00000165672	-0.176481521629766	PARK7	ENSG00000116288	0.139644690249654	0.746
**9606.ENSP00000340278**	9606.ENSP00000369127	PARK7	ENSG00000116288	0.139644690249654	DNAJA1	ENSG00000086061	0.653713495570871	0.724
**9606.ENSP00000225792**	9606.ENSP00000494750	DDX5	ENSG00000108654	-0.290947572571089	ACTB	ENSG00000075624	-0.396064192020913	0.713
**9606.ENSP00000225792**	9606.ENSP00000385269	DDX5	ENSG00000108654	-0.290947572571089	ELAVL1	ENSG00000066044	-0.119678748353951	0.703

Protein	Functional definition	Reference
DAXX	Death domain-associated protein 6; Transcription corepressor known to repress transcriptional potential of several sumoylated transcription factors.	UniProt Consortium. DAXX – Death domain-associated protein 6 (Q9UER7). UniProtKB/Swiss-Prot. Available at: https://www.uniprot.org/uniprot/Q9UER7. Accessed 2025.
SRRM2	Nuclear splicing factor involved in splice-site selection and mRNA maturation; supports AR-linked RNA processing.	Blencowe BJ. Alternative splicing: new insights from global analyses. Cell. 2006;126(1):37-47. doi:10.1016/j.cell.2006.06.023
SLU7	Splicing factor ensuring accurate 3' splice-site selection during late-stage spliceosome assembly; contributes to AR-associated post-transcriptional regulation.	Gárate-Rascón M, Recalde M, Rojo C, et al. SLU7: A New Hub of Gene Expression Regulation-From Epigenetics to Protein Stability in Health and Disease. Int J Mol Sci. 2022;23(21):13411. Published 2022 Nov 2. doi:10.3390/ijms232113411
PRPF38A	Spliceosomal protein involved in U4/U6–U5 tri-snRNP assembly and splicing regulation.	Xie J, Beickman K, Otte E, Rymond BC. Progression through the spliceosome cycle requires Prp38p function for U4/U6 snRNA dissociation. EMBO J. 1998;17(10):2938-2946. doi:10.1093/emboj/17.10.2938
ELAVL1	RNA-binding protein that stabilizes target transcripts and modulates hormone-responsive gene expression.	Abdelmohsen K, Gorospe M. Posttranscriptional regulation of cancer traits by HuR. Wiley Interdiscip Rev RNA. 2010;1(2):214-229. doi:10.1002/wrna.4
ACTB	Actin isoform functioning in nuclear architecture, chromatin regulation, and transcription factor mobility.	Miralles F, Visa N. Actin in transcription and transcription regulation. Curr Opin Cell Biol. 2006;18(3):261-266. doi:10.1016/j.ceb.2006.04.009
SMAD5	Signalling effector of BMP/TGF-β pathways that modulates transcription factor networks interacting with AR.	Kretzschmar M, Massagué J. SMADs: mediators and regulators of TGF-beta signaling. Curr Opin Genet Dev. 1998;8(1):103-111. doi:10.1016/s0959-437x(98)80069-5
PIAS1	SUMO E3 ligase modifying AR and other nuclear receptors, modulating transcriptional output and nuclear localization.	Kotaja N, Karvonen U, Jänne OA, Palvimo JJ. PIAS proteins modulate transcription factors by functioning as SUMO-1 ligases. Mol Cell Biol. 2002;22(14):5222-5234. doi:10.1128/MCB.22.14.5222-5234.2002
EP300	Chromatin acetyltransferase and nuclear coactivator required for AR engagement with chromatin.	Goodman RH & Smolik S. **“CBP/p300 in cell growth, transformation, and development.”** Genes Dev. 2000;14:1553–1577. **PMID: 10887150**
SETD1A	Histone methyltransferase (H3K4) promoting transcriptional activation of nuclear receptor target genes.	Lee JH, Skalnik DG. Wdr82 is a C-terminal domain-binding protein that recruits the Setd1A Histone H3-Lys4 methyltransferase complex to transcription start sites of transcribed human genes. Mol Cell Biol. 2008;28(2):609-618. doi:10.1128/MCB.01356-07
HOXD4	Developmental homeobox transcription factor influencing chromatin context and transcriptional programs relevant to AR.	Rijli FM, Chambon P. Genetic interactions of Hox genes in limb development: learning from compound mutants. Curr Opin Genet Dev. 1997;7(4):481-487. doi:10.1016/s0959-437x(97)80074-3
KLF8	Transcription factor controlling cell cycle and transcriptional networks intersecting with AR regulation.	Wang X, Zheng M, Liu G, et al. Krüppel-like factor 8 induces epithelial to mesenchymal transition and epithelial cell invasion. Cancer Res. 2007;67(15):7184-7193. doi:10.1158/0008-5472.CAN-06-4729
DNAJA1	HSP40 co-chaperone required for proper folding and nuclear import of AR; enhances AR stability.	Terada, K., Yomogida, K., Imai, T. et al. A type I DnaJ homolog, DjA1, regulates androgen receptor signaling and spermatogenesis. EMBO J 24, 611–622 (2005). https://doi.org/10.1038/sj.emboj.7600549
PARK7	Parkinson's disease protein PARK7 prevents metabolite and protein damage caused by a glycolytic metabolite.	Heremans IP, Caligiore F, Gerin I, et al. Parkinson's disease protein PARK7 prevents metabolite and protein damage caused by a glycolytic metabolite. Proc Natl Acad Sci U S A. 2022;119(4):e2111338119. doi:10.1073/pnas.2111338119
PRDX3	Mitochondrial peroxiredoxin supporting redox balance and protecting AR-related nuclear processes.	Gomes F, Turano H, Haddad LA, Netto LES. Human mitochondrial peroxiredoxin Prdx3 is dually localized in the intermembrane space and matrix subcompartments. Redox Biol. 2024;78:103436. doi:10.1016/j.redox.2024.103436
PRKCB	Kinase that phosphorylates AR and supports AR nuclear localization; its downregulation suggests impaired AR activation.	PRKCB protein kinase C beta [Homo sapiens (human)] Gene ID: 5579, updated on 25-Nov-2025
ITPR1	ER Ca^{2+}-release channel regulating calcium signals that modulate kinase pathways influencing AR activation cycles.	Foskett JK, White C, Cheung KH, Mak DO. Inositol trisphosphate receptor Ca2+ release channels. Physiol Rev. 2007;87(2):593-658. doi:10.1152/physrev.00035.2006
ITPR2	Inositol 1,4,5-trisphosphate receptor isoform mediating Ca^{2+} signalling linked to nuclear receptor regulation.	Foskett JK, White C, Cheung KH, Mak DO. Inositol trisphosphate receptor Ca2+ release channels. Physiol Rev. 2007;87(2):593-658. doi:10.1152/physrev.00035.2006
RRAS	Small GTPase participating in Ras/MAPK and PI3K signalling pathways upstream of nuclear receptor regulation.	Kwong L, Wozniak MA, Collins AS, Wilson SD, Keely PJ. R-Ras promotes focal adhesion formation through focal adhesion kinase and p130(Cas) by a novel mechanism that differs from integrins. Mol Cell Biol. 2003;23(3):933-949. doi:10.1128/MCB.23.3.933-949.2003
ADCY2	Adenylate cyclase generating cAMP signals influencing PKA–AR signaling cross-talk.	Willoughby D, Cooper DM. Organization and Ca2+ regulation of adenylyl cyclases in cAMP microdomains. Physiol Rev. 2007;87(3):965-1010. doi:10.1152/physrev.00049.2006
INSR	Insulin receptor activating PI3K–AKT pathways that intersect with AR signaling.	White MF. “Insulin signaling in health and disease.” Science. 2003;302:1710–1711. PMID: 14657487
PAX8	Transcription factor modulating developmental and endocrine gene programs; interacts with nuclear signalling networks.	Mansouri A, Hallonet M, Gruss P. Pax genes and their roles in cell differentiation and development. Curr Opin Cell Biol. 1996;8(6):851-857. doi:10.1016/s0955-0674(96)80087-1
FOXP1	AR-modulating forkhead transcription factor; downregulation shifts nuclear receptor specificity.	Takayama K, Horie-Inoue K, Ikeda K, et al. FOXP1 is an androgen-responsive transcription factor that negatively regulates androgen receptor signaling in prostate cancer cells. Biochem Biophys Res Commun. 2008;374(2):388-393. doi:10.1016/j.bbrc.2008.07.056
FOXP2	Forkhead transcription factor affecting nuclear transcriptional balance; upregulation alters AR regulatory circuitry.	Morgan A, Fisher SE, Scheffer I, et al. FOXP2-Related Speech and Language Disorder. 2016 Jun 23 [Updated 2023 Jan 26]. In: Adam MP, Bick S, Mirzaa GM, et al., editors. GeneReviews® [Internet]. Seattle (WA): University of Washington, Seattle; 1993-2025. Available from: https://www.ncbi.nlm.nih.gov/books/NBK368474/
CREB3L3	Stress-responsive ER-bound transcription factor regulating metabolic gene expression; linked to steroid-regulated metabolic responses.	Nakagawa Y, Satoh A, Yabe S, et al. Hepatic CREB3L3 controls whole-body energy homeostasis and improves obesity and diabetes. Endocrinology. 2014;155(12):4706-4719. doi:10.1210/en.2014-1113
DDX5	RNA helicase functioning as a nuclear AR coactivator; regulates AR-dependent transcription, chromatin association, and AR-associated splicing.	Endoh H, Maruyama K, Masuhiro Y, et al. Purification and identification of p68 RNA helicase acting as a transcriptional coactivator specific for the activation function 1 of human estrogen receptor alpha [retracted in: Mol Cell Biol. 2014 Mar;34(5):915. doi: 10.1128/MCB.01458-13.]. Mol Cell Biol. 1999;19(8):5363-5372. doi:10.1128/MCB.19.8.5363
MAP3K5	MAP3K5, also known as apoptosis signal-regulating kinase 1 (ASK1), is a key stress-responsive mitogen-activated protein kinase kinase kinase that integrates a variety of intracellular and extracellular stimuli to regulate cell fate.	Ichijo H, Nishida E, Irie K, et al. Induction of apoptosis by ASK1, a mammalian MAPKKK that activates SAPK/JNK and p38 signaling pathways. Science. 1997;275(5296):90-94. doi:10.1126/science.275.5296.90

System-level meaning	Proteins
Ubiquitin-dependent regulation of AR turnover	RNF6 (up), DLEC1 (down)
Kinase-, calcium-, and cAMP-dependent signalling modulation affecting AR activity	PRKCB (down), ITPR1 (up), ITPR2 (down), RRAS (down), ADCY2 (up), PAX8 (down), INSR (up)
Oxidative stress and chaperone-mediated modulation of AR stability	PARK7 (up), PRDX3 (down), DNAJA1 (up), NCF1 (up)
FOXP transcription factor axis affecting AR chromatin binding	FOXP1 (down), FOXP2 (up), SMAD2 (down)
Core AR transcriptional co-regulator module	EP300 (down), CREB3L3 (down), SETD1A (up)
RNA processing and spliceosome-related AR regulation	DDX5 (down), PIAS1 (up), PRPF38A (up), ELAVL1 (down), ACTB (down), SLU7 (up), SRRM2 (down), SMAD5 (up)
RAS–MAPK modulation of AR signalling	DAXX (up), RASSF1 (up), MAP3K5 (up)

nuclear androgen receptor binding

stringId_A	stringId_B	preferredName_A	ensembl_gene_id_A	log2FoldChange_A	preferredName_B	ensembl_gene_id_B	log2FoldChange_B	score
**9606.ENSP00000266000**	9606.ENSP00000351908	DAXX	ENSG00000204209	0.0638010850766978	MAP3K5	ENSG00000197442	0.460236902928167	0.999
**9606.ENSP00000266000**	9606.ENSP00000349547	DAXX	ENSG00000204209	0.0638010850766978	RASSF1	ENSG00000068028	0.802651209001862	0.948
**9606.ENSP00000078445**	9606.ENSP00000263253	CREB3L3	ENSG00000060566	-0.640357750341971	EP300	ENSG00000100393	-0.0398553990391347	0.912
**9606.ENSP00000262519**	9606.ENSP00000263253	SETD1A	ENSG00000099381	0.212828269329481	EP300	ENSG00000100393	-0.0398553990391347	0.854
**9606.ENSP00000289473**	9606.ENSP00000340278	NCF1	ENSG00000158517	0.0158466096542398	PARK7	ENSG00000116288	0.139644690249654	0.835
**9606.ENSP00000308597**	9606.ENSP00000371000	DLEC1	ENSG00000008226	-0.447945365652523	RNF6	ENSG00000127870	0.0156703603999244	0.756
**9606.ENSP00000298510**	9606.ENSP00000340278	PRDX3	ENSG00000165672	-0.176481521629766	PARK7	ENSG00000116288	0.139644690249654	0.746

score = 0.7
